# *DRD2* co-expression network and a related polygenic index predict imaging, behavioral and clinical phenotypes linked to schizophrenia

**DOI:** 10.1038/tp.2016.253

**Published:** 2017-01-17

**Authors:** G Pergola, P Di Carlo, E D'Ambrosio, B Gelao, L Fazio, M Papalino, A Monda, G Scozia, B Pietrangelo, M Attrotto, J A Apud, Q Chen, V S Mattay, A Rampino, G Caforio, D R Weinberger, G Blasi, A Bertolino

**Affiliations:** 1 Department of Basic Medical Science, Neuroscience and Sense Organs, University of Bari Aldo Moro, Bari, Italy; 2 Lieber Institute for Brain Development, Johns Hopkins Medical Campus, Baltimore, MD, USA; 3 National Institutes of Health, National Institute of Mental Health, Clinical and Translational Neuroscience Branch, NIMH, Bethesda, MD, USA; 4 Departments of Neurology and Radiology, Johns Hopkins School of Medicine, Baltimore, MD, USA; 5 Institute of Psychiatry, Department of Neuroscience, Sense Organs and Locomotive System, Bari University Hospital, Bari, Italy; 6 Departments of Psychiatry, Neurology, Neuroscience and The Mckusick-Nathans Institute of Genomic Medicine, Johns Hopkins School of Medicine, Baltimore, MD, USA

## Abstract

Genetic risk for schizophrenia (SCZ) is determined by many genetic loci whose compound biological effects are difficult to determine. We hypothesized that co-expression pathways of SCZ risk genes are associated with system-level brain function and clinical phenotypes of SCZ. We examined genetic variants related to the dopamine D2 receptor gene *DRD2* co-expression pathway and associated them with working memory (WM) behavior, the related brain activity and treatment response. Using two independent post-mortem prefrontal messenger RNA (mRNA) data sets (total *N*=249), we identified a *DRD2* co-expression pathway enriched for SCZ risk genes. Next, we identified non-coding single-nucleotide polymorphisms (SNPs) associated with co-expression of this pathway. These SNPs were associated with regulatory genetic loci in the dorsolateral prefrontal cortex (*P*<0.05). We summarized their compound effect on co-expression into a Polygenic Co-expression Index (PCI), which predicted *DRD2* pathway co-expression in both mRNA data sets (all *P*<0.05). We associated the PCI with brain activity during WM performance in two independent samples of healthy individuals (total *N*=368) and 29 patients with SCZ who performed the n-back task. Greater predicted *DRD2* pathway prefrontal co-expression was associated with greater prefrontal activity and longer WM reaction times (all corrected *P*<0.05), thus indicating inefficient WM processing. Blind prediction of treatment response to antipsychotics in two independent samples of patients with SCZ suggested better clinical course of patientswith greater PCI (total *N*=87; *P*<0.05). The findings on this *DRD2* co-expression pathway are a proof of concept that gene co-expression can parse SCZ risk genes into biological pathways associated with intermediate phenotypes as well as with clinically meaningful information.

## Introduction

Many genome-wide studies have demonstrated that complex heritable diseases such as schizophrenia (SCZ) are associated with numerous common genetic variants with small effects on susceptibility across heterogeneous populations. Risk variants are not randomly interspersed in the genome, but cluster in selective molecular pathways.^1, 2^ Therefore, molecular pathways may be more informative than any individual molecule or molecular event *per se.*^3^ As genetic variation in risk genes translates into biological risk associated with measurable phenotypes,^4^ identifying common molecular pathways that predict the phenotypes of interest may help elucidating the basis of genetic liability.

Many risk loci for SCZ are associated with the modulation of gene expression.^3, 5^ A cardinal principle of the organization of molecular pathways is that gene expression is co-regulated and pathways are likely co-expressed.^6^ This may be also the case of SCZ genes.^7^ Therefore, risk genes for SCZ may be linked through co-expression pathways.^8, 9^

Here we studied brain-specific gene co-expression as a principle to identify molecular pathways of risk genes and detect further genes related with SCZ as ‘guilty by association’. With respect to SCZ risk, the *DRD2* gene coding for the D2 dopamine receptor is an optimal candidate to investigate the genetic architecture of molecular pathways affected in patients with SCZ. A long-standing hypothesis holds that dopaminergic dysfunctional regulation in subcortical areas and in the prefrontal cortex (PFC) is a key pathophysiological mechanism of SCZ.^10, 11^ For example, working memory (WM) deficits, among the core symptoms in SCZ, are related with alterations of PFC activity.^12, 13, 14, 15, 16, 17, 18, 19^ Moreover, PFC activity during WM is predicted by midbrain dopamine,^20^ striatal dopamine^21^ and by reductions of amphetamine-induced release of prefrontal dopamine binding to D2 receptors in SCZ.^22^ Critically, research by the Psychiatric Genomic Consortium (PGC) supported the notion that the *DRD2* gene coding for the D2 dopaminergic receptor is associated with risk for SCZ.^23^ *DRD2* is not only related to SCZ risk, but genetic variation within this gene has been linked with phenotypes related to SCZ and its treatment.^24, 25, 26, 27^ Consistent with studies showing that D2 dopaminergic receptors are involved in WM,^28^ functional genetic variants in *DRD2* have also been associated with WM performance and related cortical activity in healthy subjects and in patients with SCZ.^21, 27^ For example, a non-coding single-nucleotide polymorphism (SNP) of *DRD2*, rs1076560, modulates alternative splicing of the D2 dopaminergic receptor transcript into two major isoforms (D2 long, D2L, and D2 short, D2S), likely affecting neuronal activity.^27^ However, *DRD2* single-SNP associations explain only a small fraction of risk for SCZ and related phenotypes.^21, 27^

**Figure 1:**
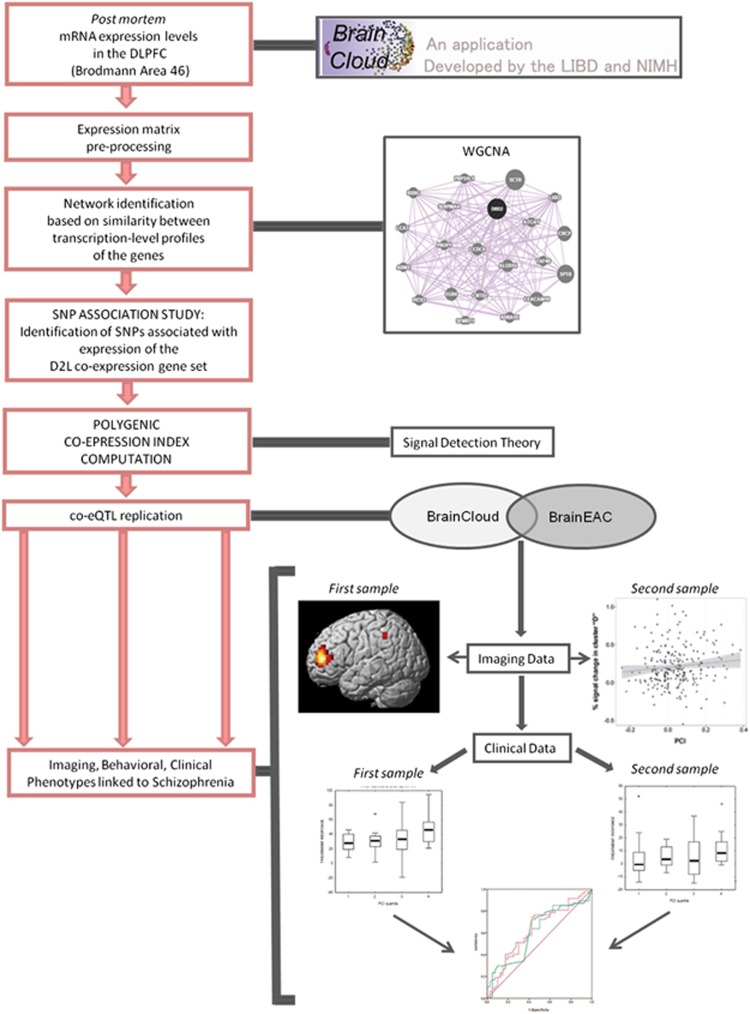
Concept of the study. DLPFC, dorsolateral prefrontal cortex; SNP, single-nucleotide polymorphism.

We investigated the compound association between SNPs approximating *in vivo* the transcription levels of a gene set co-expressed with D2L and phenotypes of relevance to SCZ. We focused on D2L in the PFC because it is mainly found at the post-synaptic terminal,^29^ its expression is altered in the PFC of patients with SCZ,^13^ and we were interested in the modulation of activity of neuronal populations receiving dopaminergic afferents during WM, a mechanism that is altered in SCZ.^22^ Furthermore, D2L is targeted by antipsychotic medications.^29, 30^ To achieve our aim, we performed four consecutive steps (Figure 1). First, we identified the co-expression gene set of D2L from human post-mortem PFC.^31^ Second, we identified the association of independent genetic variants of the genes included in the gene set with expression of the whole gene set. Third, we combined these genetic variants into a Polygenic Co-expression Index (PCI) designed to index the genetic component of gene transcription co-regulation and validated this index in an independent post-mortem data set. Fourth, we associated our PCI with brain activity and behavioral performance during WM, that is, D2L-dependent and key intermediate phenotypes for SCZ,^31^ and response to treatment with antipsychotics which target D2 signaling in SCZ. Based on prior evidence,^22^ we hypothesized that genetic variants associated with greater co-expression of D2L and its gene set would also be associated with greater BOLD signal in the PFC during WM and poorer behavioral performance. In patients with SCZ, we investigated the preservation of the PCI–BOLD signal relationships and assessed the sensitivity and specificity of the PCI as predictor of treatment outcome. Previous findings reported that the T allele of rs1076560, which is associated with a greater D2L/D2S ratio in the PFC,^2^ is also associated with greater symptom improvement in patients with SCZ^15^ Therefore, we hypothesized that greater D2L gene set co-expression in the PFC indexed by the PCI predicted greater clinical response.

## Materials and methods

### Participants

Table 1 summarizes the demographic data of the subjects included in all experiments. After receiving a complete description of the study, all participants in the clinical and the imaging studies provided written informed consent following the guidelines of the Declaration of Helsinki. Protocols and procedures were approved by the ethics committee of the University of Bari and by the institutional review board of the National Institute of Health, Bethesda, MD, USA.

### Network identification

We used the publicly available Braincloud data set^5^ (http://braincloud.jhmi.edu/) for a genome-wide Weighted Genes Co-expression Network Analysis.^32^ The sample included 199 observations (demographics in Table 1). We preprocessed the gene expression matrix to factor out confounding variables, including demographics. The first principal component of the *DRD2* gene set (module eigengene, ME) served to track the simultaneous variation of the whole gene set. We correlated this co-expression measure with *DRD2* expression levels.^32^ To investigate the biological functions that may be subserved by this ensemble of co-expressed genes, we computed gene ontology enrichment analysis using AmiGO2 (http://amigo.geneontology.org/amigo/landing). Finally, we assessed enrichment of the gene set for the loci associated with SCZ risk by the Psychiatric Genomic Consortium (PGC^23^ with a hypergeometric test (SI Materials and Methods).

### SNP association study

We performed a gene set-wide association study of SNPs with the ME. The sample size of our post-mortem data set is probably small for a genetic association study, and co-expression is most likely a phenotypic trait with complex heritability, much like SCZ. Power calculations in genetic association studies have been shown to depend on many factors, including heritability of the trait, the proportion of variance explained by the genotyped SNPs, the total number of SNPs, the proportion of SNPs with no effect on the trait, the total sample size and the *P-*value threshold for SNP selection.^33^ The heritability of expression quantitative trait loci (eQTLs) is extremely variable and appears to be comparable between *cis*- and *trans*-eQTLs (co-eQTLs fall in the latter category^34^). Nevertheless, there is evidence of high replicability of eQTLs both with stringent and more lenient thresholds of significance.^35^ These findings suggest that, beside statistical significance, genetic signals in the study of gene expression may be found beyond the threshold for corrected or nominal significance, as is also the case of complex clinical traits.^36^

Based on these considerations, we first tested for co-eQTLs that would survive stringent Bonferroni correction; then, we employed more permissive statistics for our association to minimize false-negative findings and performed internal cross-validations and independent replication to minimize type I errors. Through this procedure, we aimed at identifying an ensemble of SNPs that, together, predict co-expression. We used eight SNPs associated with the first principal component of gene set co-expression with *P*<0.005 to compute the PCI. As a common method to select SNPs for subsequent inclusion in polygenic scores consists in increasing the number of SNPs until the proportion of variance plateaus,^37^ we also tested whether such an approach selected the same set of eight SNPs with *P* <0.005 (SI Materials and Methods). We interrogated Haploreg v4.1 (http://archive.broadinstitute.org/mammals/haploreg/haploreg.php) to gain information on the possible regulatory functions of these SNPs.^38^ Then, we computed the PCI by assigning a weight to each genotype of each SNP based on the expression profile within different genotypic groups.^39^ The greater the PCI, the greater is the messenger RNA expression level of that individual. We cross-validated the PCI and assessed ethnicity and population stratification effects, as well as age effects (SI Materials and Methods).

We used the publicly available BrainEAC data set^40^ to replicate the association of the PCI with D2L co-expression in the frontal cortex. From this data set, we selected the probes of all genes included in the D2L co-expression pathway and preprocessed the data as above reported. The sample included 50 Caucasian participants.^40^ We computed the PCI of each individual and associated it with the ME using Pearson’s correlation.

#### Imaging study

We recruited 124 healthy unrelated Caucasian adults from the region of Apulia, Italy (demographics in Table 1), for a functional magnetic resonance imaging (fMRI) experiment and genotyped them for the SNPs included in the PCI. We used the n-back task to probe WM.^41^ Stimuli consisted of numbers (1–4) shown in random sequence and displayed at the points of a diamond-shaped box. There was a non-memory-guided control condition (0-back) that required subjects to identify the stimulus currently seen. As memory load increased, the task required the recollection of a stimulus seen one (1-back) or two (2-back) stimuli before, while keeping on encoding incoming stimuli (SI Materials and Methods). We tested the association of the PCI with brain activation using repeated measures analysis of covariance (within-subject factor: LOAD (1-back, 2-back); covariates: age, gender and handedness; whole-brain topological false discovery rate-corrected *α*=0.05; extent threshold=6, that is, >300 mm^3^).

We recruited a second fMRI sample of 244 Caucasian healthy volunteers as part of the NIMH Clinical Brain Disorders Branch ‘Sibling Study’ (Table 1). These participants performed the same 2-back fMRI task described above and were genome-wide genotyped (SI Materials and Methods^42^). The effect of the PCI on BOLD response was tested using robust linear models^43, 44, 45^ with age, gender and handedness as covariates of no interest. We performed one-tailed *t*-tests using as regions of interest the clusters associated with the PCI in the first healthy sample. We used MarsBar (http://marsbar.sourceforge.net/) to extract the percent signal change and corrected for the number of tests (false discovery rate^46^).

For the fMRI study on the clinical cohort, we recruited 29 Caucasian patients with SCID diagnosis of SCZ from the region of Apulia, Italy (Table 1). Genotyping and fMRI protocols matched the procedures followed for the first fMRI healthy sample.

#### Behavioral analyses

In the first healthy fMRI sample, we investigated behavioral performance (accuracy and reaction time) during the WM task in the scanning session (SI Materials and Methods). We analyzed accuracy, that is, percent of correct responses, and reaction times with repeated measures analysis of covariance (within-subject factor: LOAD (1-back, 2-back)) and PCI as a predictor. As we were testing two measures, we set the threshold for significance at *α*=0.025 (Bonferroni correction). In the clinical fMRI sample, we analyzed behavioral performance following the same procedures employed for the first fMRI sample.

#### Pharmacogenetics

The first clinical cohort in the pharmacogenetic study included 47 Caucasian patients with SCZ (Structured Clinical Interview for DSM (SCID) diagnosis)^26^ recruited from the region of Apulia, Italy (Table 1). Treatment response was computed as the difference in the total Positive And Negative Syndrome Scale (PANSS) core between baseline and treatment end.

The second cohort consisted of 40 patients with SCZ with history of inadequate treatment response recruited at the Clinical Brain Disorders Branch SCZ inpatient research unit at the National Institutes of Health Clinical Center, Bethesda, MD, USA.^26^ Treatment response was defined as the difference between symptoms severity at the end of placebo treatment and severity at the end of drug treatment.^15^ The clinical protocols used in the pharmacogenetic study for both samples have been described in detail previously^26^ (see Supplementary Table 2 for clinical data).

We first performed an association study to investigate the direction of the PCI treatment response relationship using Spearman’s Rho; then, we assessed the potency of the PCI as predictor of treatment response in comparison with pharmacological doses and off-medication symptom severity using Receiver Operating Characteristic curves (SI Materials and Methods).

## Results

### Identification of the D2L gene set

We identified a co-expression gene set of 85 genes including the D2L transcript (Supplementary Table 1). Its first principal component (ME) explained 32.5% of the variance. D2L expression levels positively correlated with ME (*R*^2^=0.4). The D2L co-expression pathway was enriched for the ontologies DNA packaging (GO:0006323, corrected *P*-value=0.002), negative regulation of dopamine secretion (GO:0033602, corrected *P*-value=0.004) and response to nicotine (GO:0035094, corrected *P*-value=0.03); notably, some of these results may be affected by our choice of the *DRD2* module as a candidate gene set. Besides *DRD2*, this gene set included three genes associated with SCZ based on PGC2 (*GATAD2A*, *GALNT10* and *ZSCAN23*). The enrichment of the gene set for protein-coding genes located in the genome-wide association study loci associated with SCZ by PGC2^23^ was significant (hypergeometric test, *P*=0.029).

### Identification of co-eQTLs associated with co-expression of the whole D2L module

Eight independent SNPs located in the genes included in the module were associated with co-expression of the whole gene set, that is, the ME (Table 2). The first SNP, rs2486064, survives even very stringent statistics using Bonferroni correction for multiple comparisons (corrected *P*=0.0033). Table 2 shows that these SNPs are not strong predictors of SCZ status in the PGC2 work.^23^ Five out of these eight non-coding SNPs modify regulatory motifs^47^ and two out of eight have been previously recognized as *trans*-eQTLs.^48^ As an ensemble, these SNPs are strongly associated with gene expression regulation in the dorsolateral prefrontal cortex Haploreg v4.1, *P*=0.00049, SI Appendix). We used these SNPs to compute the PCI and to verify its correlation with expression of the whole gene set (as per definition of the PCI; *R*^2^=0.38) and D2L transcriptional levels (*t*_198_=5.8, *R*^2^=0.14, *P*=2.9 × 10^−8^; Supplementary Figure 1). These effects were not affected either by population stratification or by age (Supplementary Table 3; SI Results). Multiple cross-validations performed using different procedures uniformly supported the association between co-expression of the gene set and the PCI (Supplementary Figures 2 and 3).

As in spite of all *in silico* validations it cannot be definitively ruled out that our findings were related with peculiarities of the specific sample analyzed, for example, with ethnicity, we replicated the association between the PCI and the gene set using an independent data set (BrainEAC^40^). BrainEAC included 80 out of the 85 probes of the D2L gene set above identified and was not affected by ethnicity effects because it only included Caucasian subjects. The correlation between the PCI and gene set co-expression replicated in the same direction (Pearson’s *R*=0.23, one-tailed *P*=0.05). The association was strongest for highest quality observations (for example, with RNA Integrity Number (RIN)>6, *R*=0.38, one-tailed *P*=0.028; Supplementary Figure 4). This is relevant because all Braincloud data had RIN>7, thus we found a significant association in spite of non-overlapping probes, of partly degraded messenger RNAs and of ethnicity differences.

### Biological validation of the PCI by means of association with system-level phenotypes: imaging study

**Figure 2:**
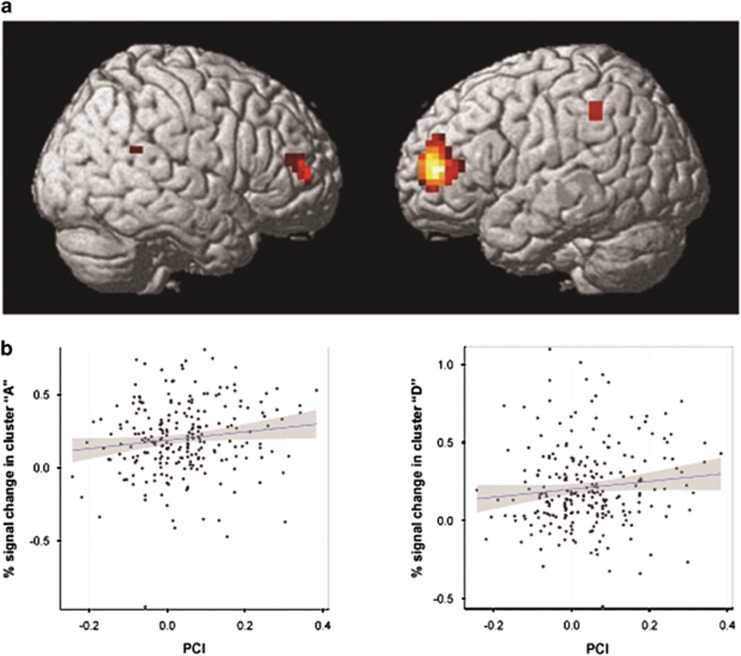
Discovery and replication functional magnetic resonance imaging results. (**a**) First sample. Significant clusters associated with the positive slope of the Polygenic Co-expression Index (PCI) at topological false discovery rate-corrected *q*-value <0.05 (cluster extent=6). (**b**) Second sample. Scatter plots of the % signal change in two clusters with significant positive correlation with the PCI in the second sample. The regression line is blue, 95% confidence intervals for the fit are gray. See Table 3 for the exact statistics and localization of clusters ‘A’ and ‘D’. Left in the figure is left in the brain.

In the first healthy sample, activity in the fronto-parietal WM network correlated positively with the PCI surviving whole-brain peak-level correction for multiple comparisons (topological false discovery rate *q*<0.05; Figure 2 and Table 3). Individuals with greater PCI and greater predicted D2L gene set co-expression levels had greater bilateral activation in the WM brain network during task performance, that is, they were less efficient in processing WM information. There was no significant negative correlation with the PCI or LOAD × PCI interaction on brain activity. Further analyses supporting the robustness of these results are reported in Supplementary Tables 3–4 and Supplementary Figures 5–6.

In the second healthy sample, we used the clusters identified in the first sample to extract the signal change which we associated to the PCI. We found significant correlations between the PCI and percent signal change in the left anterior middle frontal gyrus (BA10), and in the right inferior parietal lobule (BA40; Table 3). Also in patients, brain activity correlated with the PCI (Supplementary Figure 7). Hence, the PCI–BOLD association was preserved in patients with SCZ.

### Behavioral results

In the first healthy fMRI sample, repeated measures analysis of covariance (within-subject factor LOAD (1-back, 2-back)) on WM accuracy and reaction times revealed a significant LOAD × PCI interaction on reaction times surviving Bonferroni correction (F_1,120_=6.9, *P*=0.01). *Post hoc* regressions were non-significant for 1-back (*t*_120_=0.12, adjusted *R*^2^=−0.008, *P*=0.91) but yielded a significant fit for 2-back (Supplementary Figure 6; *t*_120_=2.3, adjusted *R*^2^=0.033, *P*=0.024). Greater PCI was related with longer reaction times at 2-back (Supplementary Figure 8), supporting the imaging findings and the interpretation of reduced efficiency in these individuals. No other significant effects or interactions involved the PCI (SI Results). The same analysis on behavioral data of the clinical fMRI sample yielded no significant effects or interactions (all *P*>0.05).

### Clinical translation of the PCI by means of association with response to treatment with antipsychotics

The correlation between clinical improvement (difference in PANSS total score between baseline and end point) and PCI was positive and significant in the first clinical sample (*N*=47, *ρ*=0.39; *P*=0.007; Supplementary Figure 9). Results replicated in the second clinical sample (PANSS total score, *N*=40, *ρ*=0.27, one-tailed *P*=0.047). Greater PCI was associated with greater clinical response. Then, we pooled the two samples (*N*=87) and tested the blind prediction of treatment response based on the PCI, which was significant (area under the curve (AUC)=0.63, *P*=0.043; Supplementary Figure 10). Similarly, off-medication symptoms (AUC=0.62, *P*=0.048) significantly predicted treatment response, whereas dose adjustment was a marginally significant predictor (AUC=0.61, *P*=0.079).

## Discussion

We believe the present results provide the first proof of concept that the co-expression context of SCZ risk genes, for example, *DRD2*, affects system-level and clinical phenotypes. Although previous studies indexed gene expression based on *cis*-genetic markers,^39, 49^ here we detected co-expression *trans*-eQTLs (co-eQTLs) to predict gene co-expression using a network approach. This data-driven, bias-free procedure identified a molecular pathway of convergence of some genes associated with risk for SCZ. The D2L network recapitulates part of the complex neurobiology of SCZ and other systems level phenotypes, including WM performance and related brain activity.

### Genes co-expressed with D2L

We found that the variability of system-level phenotypes is closely associated with a shared component between co-expressed genes. Indeed, the statistics of the association between the PCI and imaging phenotypes outperform prior reports on the effect of genetic variants associated with *DRD2*.^26, 27^ Moreover, the effect of the PCI survived when we co-varied for *DRD2* rs1076560 genotype (SI Results). Interestingly, our gene set included genes previously associated with biochemical pathways relevant to SCZ, for example, calcium- and cannabinoid-mediated transmission like *CACNA2D4* (ref. 50) and *CNR1*.^51^ The gene set also includes genes not mentioned in the latest PGC2 publication, but associated with SCZ in previous genome-wide association studies, such as *CALHM3*.^52^ Interestingly, one of the set genes associated with SCZ, *GATAD2A*, ranked fourth for intramodular connectivity, hence belonging to a group of highly connected genes (hubs) within the identified network. *GATAD2A* is involved in gene silencing and is associated with histones.^53^ Accordingly, the gene set includes further histone-related genes (*HIST1H1E*, *HIST1H3G* and *HIST2H2AC*) and several of the SNPs included in the PCI are associated with histone functions (SI Appendix; see also the SI Discussion for more information on the SNPs identified). Histone proteins have been highlighted in a recent *trans*-diagnostic gene set analysis of the genetic architecture of psychiatric disorders.^23^

Because of the data-driven nature of the approach, genes associated by prior literature with D2 dopamine receptors, for example, by physical interaction, may end up in different clusters when building a network. Therefore, not all genes relevant to D2L-mediated signaling are included in the gene set studied here. However, a tenet of co-expression analyses is that co-expressing genes are co-regulated in terms of transcription, for example, they may be targeted by the same repressors/enhancers or si/miRNA. It is also important to note that the network we identified is a model of co-expression, and does not necessarily represent gene co-regulation. For example, gene expression patterns vary as a function of many variables, such as age,^5^ ethnicity and cell type. Here we clustered genes based on their expression patterns corrected for confounding variables. The SI Materials and Methods includes additional information on age and ethnicity effects (SI Results).

Because of the relatively small sample size of post-mortem data sets for a genetic association study, we used permissive procedures for co-eQTL detection. Then, we cross-validated the SNP weights and also the SNP selection within Braincloud, we replicated the effects in BrainEAC, and we also cross-validated the SNPs entering the PCI in the fMRI experiment. Together, these validation steps and the findings obtained in multiple independent data sets across multiple biological scales support the idea that the SNPs identified here are valid and modulate the D2L co-expression pathway. It is remarkable that eight common SNPs weighted for their molecular effects on gene co-expression accounted for a sizable proportion of variance in prefrontal activation and significantly predicted treatment outcome in patients with SCZ (SI Discussion).

The SNPs associated with the D2L co-expression gene set that we identified have not been previously reported for their association with *DRD2* or with psychiatric or cognitive phenotypes, except for rs1037791 that has been associated with the openness subscale of the big five personality traits.^54^ However, the first-ranked SNP, which even survived Bonferroni correction for multiple comparisons, is intergenic between two paralog genes, *CHIT1* and *CHI3L1*. Both genes code for stress-induced chitinases and prior evidence associated brain *CHI3L1* expression with SCZ.^55, 56^ Genetic variants in this intergenic region such as rs4950928 have been associated with SCZ.^57, 58^ This SNP is located upstream of *CHI3L1* where also rs2486064 is located (linkage disequilibrium: *r*^2^=0.35, D′=1). This regulatory region likely interacts with specific transcription factors.^57, 58^ *CHI3L1* expression has been proposed to be relevant to SCZ because of its association with the *AKT1*–*GSK3β* pathway,^58^ which is also associated with the cyclic AMP-independent pathway of *DRD2*.^59^ The present evidence suggests a further candidate molecular mechanism of action of genetic variants located in this region in SCZ, that is, regulation of a *DRD2* co-expression gene set.

### The association of the D2L co-expression PCI with prefrontal activity and WM performance

Dopamine D2 receptor signaling is a critical modulator of WM. The present findings suggest that individuals bearing genotypes associated with increased simultaneous expression of the D2L gene set manifest greater PFC activity as well as longer reaction times during WM. These findings suggest that increased expression of the D2L co-expression gene set predisposes to less efficient WM processing, a well-established intermediate risk-associated phenotype for SCZ.^31^ These results are also consistent with a large body of studies demonstrating that pharmacological manipulation of dopamine D2R is associated with WM performance and with prefrontal activity measured with BOLD fMRI.^41, 60^ Critically, they are consistent with a recent study reporting that greater prefrontal D2 PET binding is positively correlated with prefrontal activity during WM.^22^ Finally, these findings are consistent with several reports from our group suggesting that the T allele of rs1076560, associated with greater D2L/D2S ratio compared with the G allele, is also associated with inefficient prefrontal activity during WM.^21, 27, 41^ The present findings extend this earlier work by showing that the co-expression gene set identified by our network analysis is associated with genetic background outside the *DRD2* gene. Such genetic variation is in turn associated with intermediate phenotypes of SCZ.

### The role of D2L and its co-expression gene set in treatment response to antipsychotics in patients with SCZ

D2 dopaminergic receptor is the main target of antipsychotic medications^29, 30^ and previous evidence indicated that T-carriers for rs1076560 benefit more from antipsychotic treatment than other patients with SCZ.^26^ Consistently, here we show that greater predicted expression of a D2L co-expression gene set is associated with greater clinical improvement. We found this association not only in drug-naive/drug-free patients but also in patients with history of inadequate treatment response. The strength of the prediction based on the PCI compared favorably with clinical predictors such as pharmacological dose. Results suggest that SCZ patients with greater genetically determined availability of a main target of antipsychotics are predisposed to better treatment response.

## Conclusions

The present findings suggest that a D2L co-expression gene set enriched for protein-coding genes associated with schizophrenia modulates PFC function during WM and response to D2 antagonist antipsychotic drugs. The genetic variants detected in this study were not associated with diagnosis, but were located in regulatory genetic loci. In other words, genetic variation modulating molecular pathways of SCZ risk genes may recapitulate part of the variance of SCZ-related phenotypes in healthy and clinical populations.

## Figures and Tables

**Table 1:** Demographic data in all studies reported

*Sample name*	*Sample size*	*Female (male) [ratio]*	*Age mean±s.d. years*	*Age range (years)*
Braincloud	199	60 (139) [0.43]	32.3±20	0–78
BrainEAC	50	15 (35) [0.43]	57±19	20–91
First healthy fMRI study	124	54 (70) [0.77]	27.5±6.6	19–48
Second healthy fMRI study	244	132 (112) [1.20]	29.4±8.4	18–55
fMRI patients study	29	3 (26) [0.12]	29.3±7.0	15–42
First clinical study	47	8 (39) [0.21]	28.7±7.0	16–42
Second clinical study	40	11 (29) [0.38]	27.4±6.5	18–40

Abbreviation: fMRI, functional magnetic resonance imaging.

**Table 2:** SNPs associated with the first principal component of D2L gene set expression

*Rank*	*Marker*	*Locus*	*Gene*^a^	*Gene name*	*Uncorrected* P*-value*	*MAF*	*PGC* P*-value*
1	rs2486064	1q32.1	CHIT1	Chitinase 1	5.0 × 10^−6^	0.22	0.088
2	rs6902039	6p22.3	GPLD1	Glycosylphosphatidyl inositol-specific phospholipase D1	4.6 × 10^−4^	0.23	0.48
3	rs851436	2p24.1	OSR1	Odd-skipped related 1	1.0 × 10^−3^	0.48	0.77
4	rs9297283	8q22.2	POP1	Processing of precursor 1, ribonuclease P/MRP subunit	1.0 × 10^−3^	0.20	0.33
5	rs12940715	17q25.1	SDK2	Sidekick cell adhesion molecule 2	1.7 × 10^−3^	0.20	0.050
6	rs1805453	17p13.2	DHX33	DEAH (Asp–Glu–Ala–His) Box Polypeptide 33	2.8 × 10^−3^	0.34	0.054
7	rs11213916	11q22.3	BTG4	B-cell translocation gene 4	3.0 × 10^−3^	0.30	0.50
8	rs1037791	7p21.1	AGR2	Anterior gradient 2	3.2 × 10^−3^	0.31	0.94

Abbreviations: MAF, minor allele frequency in the Braincloud sample; PGC, Psychiatric Genomics Consortium; SNP, single-nucleotide polymorphism.

a The Gene column reports the genes included in the D2L gene set. SNPs fall in a ±100 kbp window from the set genes.

*P*-values refer to the association with diagnosis of schizophrenia.

**Table 3:** Statistics of the association between PCI and brain activity during working memory*

*First healthy fMRI sample*									*Second healthy fMRI sample*		
*Cluster*	*Region/BA*	*MNI (*x, y, z*)*	*Talairach (*x, y, z*)*	*Cluster extent*	Z*-value*	*Uncorrected* P*-value*	*Corrected* P*-value*	*Partial* η^*2*^	t*-value*	*Uncorrected* P*-value*	*Corrected* P*-value*
A	**Left MiFG/10**	**−33, 49, 14**	**−33, 47, 13**	**156**	**4.8**	**<0.001**	**0.002**	**0.13**	**2.6**	**0.0052**	**0.01**
B	Right MiFG/10	31, 46, 14	30, 44, 14	26	3.44	<0.001	0.027	0.08	0.26	0.40	0.40
C	Right STG/13	46, **−**44, 21	45, **−**42, 22	7	3.36	<0.001	0.031	0.07	0.73	0.23	0.31
D	Left IPL/40	**−**59, **−**41, 44	**−**58, **−**38, 41	12	3.32	<0.001	0.033	0.07	**3.1**	**0.0011**	**0.0044**

Abbreviations: BA, Brodmann area; FDR, false discovery rate; fMRI, functional magnetic resonance imaging; IPL, inferior parietal lobule; MiFG, middle frontal gyrus; MNI, Montreal Neurological Institute; STG, superior temporal gyrus.

First study: Displayed clusters survive peak-level topogical FDR-corrected threshold *q*=0.05, cluster extent=6. Results also surviving cluster-level family-wise error (FWE) correction for multiple comparisons are in bold font. Partial *η*^2^ refers to the effect size of the association of brain activity with the Polygenic Co-expression Index (PCI).

Second study: Results surviving FDR correction for multiple comparisons (number of tests=4, *α*=0.05) are in bold font.

*White fields refer to the first healthy fMRI sample, grey fields to the second healthy fMRI sample study.
