# Supplementary material for: DRD2 co-expression network and a related polygenic index predict imaging, behavioral and clinical phenotypes linked to schizophrenia
Source: Transl Psychiatry. 2017 Jan 17;7(1):e1006–. doi: 10.1038/tp.2016.253 (PMC5545721; doi:10.1038/tp.2016.253)
Supplement: Supplementary Information [file tp2016253x1.pdf]

SUPPLEMENTARY INFORMATION

***DRD2* Co-expression Network and a related Polygenic Index predict Imaging, Behavioral, and Clinical Phenotypes linked to Schizophrenia**

INVENTORY OF SUPPLEMENTARY INFORMATION [SI]

1. SI MATERIALS AND METHODS

WGCNA

Expression matrix pre-processing

Network identification

SNP ASSOCIATION STUDY

POLYGENIC CO-EXPRESSION INDEX COMPUTATION

co-eQTL replication

Association of the module eigengene and of D2L expression with genomic principal components

Association of the Polygenic Co-expression Index with age

IMAGING STUDY

Participants

Genotyping

Working memory task

fMRI data acquisition and analysis

Association of the Polygenic Co-expression Index with brain activity and behavior during working memory performance

Imaging replication in healthy subjects

Participants

fMRI data acquisition and analysis

Association of the Polygenic Co-expression Index (PCI) with BOLD response during working memory performance

Imaging study in patients with schizophrenia

PHARMACOGENETIC STUDY

Participants and clinical protocols

Statistical procedures

2. SI RESULTS

WGCNA

SNP ASSOCIATION STUDY AND CO-EXPRESSION POLYGENIC INDEX COMPUTATION

Association of the Polygenic Co-expression Index with genomic principal components

Association of the Polygenic Co-expression Index with age

IMAGING STUDY

Supplementary fMRI analyses in healthy individuals

Association between PCI and brain activity during working memory in patients with schizophrenia

Complete statistics of the behavioral analysis reported in the main text

PHARMACOGENETIC STUDY

3. SI DISCUSSION

Considerations on the pharmacogenetics studies reported

4. SI FIGURE LEGENDS

5. SI REFERENCES

6. SI TABLES

7. SI APPENDIX

1. SI MATERIALS AND METHODS

WGCNA

Braincloud includes *post mortem* mRNA expression levels of healthy subjects in the DLPFC (Brodmann Area 46) obtained with oligonucleotide microarray. We selected post-natal samples based on ethnicity and RNA integrity (RIN). Only observations with RNA integrity (RIN) ≥ 7.0 were included[1]; furthermore, the final sample only included Caucasian and African American subjects because of the small sample size of Hispanic and Asian groups (Hispanic N=6; Asian N=4).

Expression matrix pre-processing

Braincloud includes 30 176 probes. We included three types of probes: hHC (human constitutive exonic), hHA (human alternative exonic) and hHR (human mRNA). We excluded control probes (hCX and hCT), human ESTs (hHE) and human other (hHO; see Colantuoni et al.[2]), for further details). Moreover, we excluded unnamed genes (e.g., cORF), except for protein-coding genes located in the loci associated with schizophrenia by the PCG consortium[3]. For genes with multiple probes highly correlated with each other (Bonferroni-corrected $\alpha < 0.01$, i.e. Pearson's $r = 0.36$), we selected the ones with the greatest variance[4].

Meta-data were available in Braincloud for each subject, i.e., demographical variables (age, sex, ethnicity) and sample quality features (RIN, pH, post-mortem interval). Since confounding variables may affect expression estimates[1,5], we factored out their effects from each gene's expression by means of a stepwise linear model. The Akaike Information Criterion served to select the best set of predictors for each individual gene. The model included the main effect of the six variables above mentioned and the age-sex, age-ethnicity, sex-ethnicity and the age-sex-ethnicity interaction terms. Continuous variables that significantly deviated from the normal distribution in

our sample (Shapiro-Wilk test) underwent rank-based inverse normal transformation following Blom procedure[6].

Finally, each column of the table, i.e., gene expression, was transformed to reduce the impact of outliers and deviation from normality. Thus, we obtained a 199 × 23 636 matrix of Blom-transformed expression residuals reflecting for each subject transcription levels of all genes relative to the entire sample considered. These variables were used for network identification.

Network identification

WGCNA results in topological representation of the relationship between genes which can be used to define clusters of co-expressed genes (gene sets). Network identification is based on similarity between transcription-level profiles of the genes included across individuals. We used functions provided by WGCNA R package to build a Weighted Co-expression Network. First, we verified scale-free topology for any $1 < \beta < 20$ and we chose the lowest value of β that satisfied the criterion ($R^2 > 0.8$ [(7)]). The scale-free topology criterion was satisfied for $\beta = 4$ ($R^2 = 0.86$). The adjacency matrix was calculated by raising the correlation matrix to the selected β as determined by soft thresholding. Minimum gene set size was 40 genes and minimum height for merging gene sets was 0.05.

SNP ASSOCIATION STUDY

We focused our attention on the gene set that included the probe designed to identify the mRNA that is translated into the D2L. This transcript is one of the two products of alternative splicing involving exon 6 of *DRD2*. D2L is coded by all exons, whereas the short isoform (D2S) does not include exon 6. The probe we identified matched precisely exon 6 of *DRD2*. We computed enrichment of the gene set for genes located in the loci identified by the Psychiatric Genomic

Consortium (PGC) by using a hypergeometric model. We obtained a list of the protein-coding genes encompassing 500 kbp up- and downstream to the SNPs identified by PGC. We considered the genes in this list as hits for the hypergeometric test.

Braincloud includes for each subject the genotypes of 654 333 SNPs spanning the whole genome. We selected for further analyses those SNPs that were also available in an independent dataset of participants recruited by our group (there were 360 119 SNPs available). Moreover, SNPs significantly deviating from Hardy-Weinberg equilibrium ($\alpha = 0.003$) or with a Minor Allele Frequencies (MAF) < 0.1 were not included because the sample size was too limited to investigate rare variants. We used SnpVariationSuite (SVS; GoldenHelix, Bozeman, Montana) to map genes encompassed in the gene set into RefSeq Genes 63 UCSC build NCBI36[8]. We selected 2 046 SNPs falling into a window of 100kbp up- and down-stream each gene in the co-expression module. We evaluated pair-wise R^2 between markers within the same chromosome. We considered two SNPs independent when $R^2 < 0.1$[(3)]. We then performed a priority LD pruning by iteratively discarding the SNP with the weaker association (lower F-value) with the *ME.* We used this procedure to enrich our selection for relevant variants (for further applications of a similar procedure see http://prioritypruner.sourceforge.net/documentation.html). The final selection included 658 SNPs with low residual interdependence. We ran a *genotypic model* in SVS that performs one-way ANOVAs separately for each SNP, selecting those which survived at $\alpha = 0.005$ (uncorrected). This procedure does not allow, per se, the generalization of the findings to unseen subjects. Instead, we addressed the generalizability of this SNPs with specific cross-validation procedures and replication in an independent post mortem dataset (see below).

Nine independent SNPs were found associated with the *ME* and were thus selected for the computation of the PCI. We pooled the minor homozygous and the heterozygous samples whenever the sample size of the minor homozygous group was smaller than 10 to avoid biasing statistics and double-checked whether the association was still significant. We checked if *ME* and each allelic

population complied with the normal distribution through Shapiro-Wilk tests. One of the SNPs (rs6504631) was excluded because the association with *ME* after pooling minor homozygous with heterozygous subjects was no longer significant.

POLYGENIC CO-EXPRESSION INDEX COMPUTATION

The computation of the PCI is based on Signal Detection Theory. We used the Discriminability Index D' to quantify the magnitude of the differences in the *ME* between the three genotypic populations of each of the eight SNPs selected. For each SNP, we used the major allele homozygote population (MH) as reference group. The criterion for D' computation was set at the average *ME* of the MH sample. Thus, D' is a measure of how *ME* discriminated the heterozygous and the minor homozygous samples from the MH group. By this definition, D' of the MH group for each SNP is 0. Positive D' implies greater co-expression levels compared to the MH population and *vice versa*. The procedure to compute the PCI has been described in detail by Pergola et al.[9].

After having defined D' for each SNP, we defined the PCI of each participant as the arithmetic mean of D' of the selected SNPs. The greater the PCI, the greater the mRNA co-expression level of that individual.

We performed leave-one-out and k-out cross-validation of the weights of the SNPs in the PCI. These analyses tested whether the eight SNPs included in the PCI are subject to biases caused by influential observations. We defined 7 training sets of different sizes (N_{train} = 40, 60, 80, 100, 120, 140, 160). For each training set we performed 10 000 random permutations and computed the PCI in the out-of-train observations, i.e., the test set. Finally, we assessed the association between the PCI in the test set and the *ME* for each permutation of each training set. Furthermore, we performed a K-fold cross-validation in which we kept all samples independent as a further test of the robustness of the results. This procedure rules out that the cross-validation results are driven by a subgroup of the subjects which, as an ensemble, bias the results.

We performed an ANCOVA with ethnicity and PCI as predictors and the *ME* as dependent variable to assess whether the association between PCI and *ME* differed among ethnicity groups. Moreover, we computed the Pearson's r between the PCI and the *ME* in the two groups separately.

Finally, we asked whether our priority pruning biased SNP selection. In principle, this procedure yields a risk of overfitting because the seed for LD pruning is not random, but based on the target variable. The rationale is to keep the most important genetic loci and make SNPs statistically independent, which is a requirement of the PCI computation. To validate the SNP selection, we performed three analyses: i) we computed a leave-one out cross validation which, at each iteration, selected the first eight SNPs (i.e., as many as in the PCI) and associated the resulting PCI with the module eigengene in the left-out subject; ii) we computed 100 random-seed LD pruning and associated the resulting SNPs with the ME; then, we linked each SNP with the closest module gene and obtained a ranking of the genes based on their most significant SNP. We correlated these 100 rankings with the ranking obtained by our priority pruning and compared this population of correlations with that obtained through 100 random gene lists (null distribution). A large difference between the correlations obtained and the null distribution would suggest that our priority pruning does not bias the relationship of the genes with the module eigengene as assessed by the co-eQTL analysis. We assessed the difference using Cohen's d. iii) we evaluated the inclusion of increasing numbers of SNPs in the PCI. The rationale of this analysis is to select the SNPs that together explain the largest possible proportion of variance. Through this procedure, the PCI should be enriched for true positive markers[10]. In particular, we computed a series of PCIs following the ranking of the priority pruning. So, for instance, the first ranked SNP was included in the first PCI; the first two SNPs were included in the second PCI and so on until the 100th SNP. Critically, as in point i), we calculated the PCIs through a leave-one-out cross-validation. Therefore, for each individual we computed the PCI based on the SNP weights derived from the other 198 subjects. This procedure made the test set (i.e., the left out subject) independent of the training set

(i.e., the 198 remaining subjects). Then, we correlated each of the 100 cross-validated PCI with the *ME* (first principal component of gene expression). Notably, to compute the *ME* we did not use the left out subject, but obtained the *ME* using the loadings of the first principal components in the training sample. Then, we evaluated and plotted the correlations. Following this analysis, we computed the percent change of the correlations in the PCI series. In particular, we computed a series of percent correlation changes between the second and the first PCI, the third and the second, and so on. We defined the percent correlation change as $(r_{j+1} - r_j)/r_j$, where r represents the Pearson's correlation between the PCI and the *ME*. We plotted the percent correlation change. All of these analyses were performed using custom-written scripts in the software R 3.0.2 64-bit.

co-eQTL replication

We assessed the overlap between the BrainEAC and the Braincloud probes by comparing the nucleotide sequences (see Supplementary Table 1 for details). Of the 80 probes which were present both in Braincloud and BrainEAC, 37 probes were fully overlapping based on their nucleotide sequence, 28 had partial overlap with the Braincloud probes, 15 showed no overlap. For the latter genes we used the gene-level expression estimates in BrainEAC. BrainEAC differs from Braincloud as far as RIN is concerned. Data were available for 127 healthy subjects from *post mortem* Frontal Cortex brain tissue; we removed 4 outliers for pH (iterative Grubbs test, all $p < .05$) and all observations with RIN ≤ 5 in the Frontal cortex. This decision was taken as a trade-off between comparability across datasets (only samples with RIN ≥ 7 were included in the analysis on Braincloud data) and sample size. The final sample included 50 healthy Caucasians. Data were then preprocessed as for the discovery co-eQTL study and we extracted the module eigengene. Finally, we computed the PCI of each individual and associated it with the *ME* using Pearson's correlation to replicate the co-eQTL results. Since our a priori hypothesis was that the direction of the

correlation would be the same as in the discovery sample we used one-tailed probabilities and set our $\alpha = .05$.

Supplementary Table 1 about here

Association of the module eigengene and of D2L expression with genomic principal components

We used the R package SNPRelate to compute genomic principal components (GPCs). Braincloud includes for each subject the genotypes of 654 333 SNPs spanning the whole genome. We pruned markers by linkage disequilibrium using $r^2 \leq 0.9$ to alleviate LD bias[11]. We extracted 10 PCs and tested the Spearman correlation with the *ME* and with D2L expression.

Association of the Polygenic Co-expression Index with age

Gene expression profiles are susceptible to changes along development and aging. In this study we kept all post-natal samples in the analysis to obtain greater statistical power. We tested whether our preprocessing pipeline was effective at removing age effects by correlating the expression of the eight genes reported in Table 2 in the main text with age. Then, to ensure that the PCI predicted co-expression in the age range included in our samples, we assessed the PCI/co-expression correlation in samples with restricted age range. As shown in Table 1 in the main text, the samples we studied with fMRI, behavioral and clinical measures included participants between 15 and 55 years old, thus we restricted the Braincloud and BrainEAC samples to this interval.

IMAGING STUDY

Participants

All subjects were evaluated with the Structured Clinical Interview for the Diagnostic and Statistical Manual of Mental Disorders 4th Edition, to exclude any psychiatric disorder. All

participants in the imaging study were genome-wide genotyped using microarray technology. Along with the absence of psychiatric conditions, other exclusion criteria were represented by familial risk for psychiatric disorders (first-degree relatives of patients were excluded), history of drug or alcohol abuse, active drug use in the previous year, head trauma with loss of consciousness, presence of metal implants on participants' body, and any relevant medical condition. Moreover, data affected by scanning artifacts or showing excessive movement during the scanning sessions were not further analyzed, leading to exclusion of the participant. Participants who performed so poorly in the cognitive task that their accuracies could not be discriminated from chance level were excluded. The final sample included 124 healthy, unrelated, Caucasian participants. Participants took part in a separate session aimed to collect socio-demographic and neuropsychological information by means of semi-structured interviews. We used the Edinburgh Handedness Inventory[12] to assess hand preference.

Genotyping

Participants in the imaging study underwent blood withdrawal for subsequent DNA extraction from peripheral blood mononuclear cells. To this aim, approximately 20ml of fresh blood was obtained through a conventional venous blood collection with 10ml EDTA Vacutainer Venous Blood Collection Glass Tubes (Vacutainer ®). Approximately 200 ng DNA was used for genotyping analysis. DNA was concentrated at 50ng/µl (diluted in 10 mM Tris/1mM EDTA) with a Nanodrop Spectrophotometer (ND-1000). We used Illumina HumanHap550K/610Quad Bead Chips (San Diego, California) to genotype our sample. Briefly, each sample was whole-genome amplified, fragmented, precipitated and resuspended in appropriate concentrations of hybridization buffer. Denatured samples were hybridized on prepared Illumina Human550K/610-Quad Bead Chips. After hybridization, the Bead Chip oligonucleotides were extended by a single labeled base, which was detected by fluorescence imaging with an Illumina Bead Array Reader. Normalized bead

intensity data obtained for each sample were loaded into the Illumina GenomeStudio (Illumina, v.2010.1) with cluster position files provided by Illumina, and fluorescence intensities were converted into SNP genotypes. After genotypes were called and the pedigree file was assembled, we removed SNPs showing minor allele frequency <1%, genotype missing rate >5%, or deviation from Hardy-Weinberg equilibrium (p<0.0001). Individuals were also removed if their overall genotyping rate was below 97%. Sample duplications and cryptic relatedness were ruled out through identity-by-state (IBS) analysis of genotype data.

Working memory task

The N-Back task consisted of two runs each with two conditions. Participants did not exit the scanner during the breaks. The first block featured 0-back and 1-back, the second featured 0-back and 2-back. The stimuli were arranged in a block design, consisting of eight 30-s blocks: four blocks of the control condition alternating with four blocks of each WM. Each block began with task instructions (2 s) and included 14 task trial (duration: 0.5 s, inter-trial interval: 1.5 s). Stimuli were presented via a back-projection system and behavioral responses were recorded through an optic fiber response box which allowed assessment of accuracy (percentage of correct responses) and reaction time for each trial. Numbers appeared at a fixed location, which was highlighted by a circle within the diamond-shaped box. Participants responded with their right hand with a four-button MRI-compatible pad within the scanner.

fMRI data acquisition and analysis

Blood oxygen level-dependent (BOLD) signal was recorded by a GE Signa 3T scanner (General Electric, Milwaukee, WI), equipped with a standard quadrature head coil. A gradient-echo planar imaging sequence, (repetition time, 2000 ms; echo time, 28 ms; 20 interleaved axial slices; thickness, 4 mm; gap, 1 mm; voxel size, 3.75 × 3.75 × 5 mm; flip angle, 90°; field of view, 24 cm;

matrix, 64 x 64) was used to acquire 120 volumes while subjects performed the WM task. The first four scans were discarded to allow for equilibration effect. We used the Matlab (Mathworks, Natick, Massachusetts, USA) toolbox Statistical Parametric Mapping 8 (SPM8; http://www.fil.ion.ucl.ac.uk/spm) to analyze the fMRI data using standard procedures.

We realigned the images to correct for motion artifacts. Images of each subject were pre-processed using the Realign and Unwarp function (SPM8), in order to compensate for non-linear signal distortions induced by head motion. Movement parameters were extracted to exclude subjects with excessive head motion (2 mm of translation or 2° of rotation). Realigned images were resliced to a 3.75 mm isotropic voxel size and spatially normalized into a standard space (Montreal Neurological Institute) by using a 12 parameter affine model.

Then, we smoothed the scans (12 mm full-width at half-maximum isotropic). Effects of condition at individual subject level were evaluated producing a *t* statistical map for the contrasts of interest (1-back versus 0-back and 2-back versus 0-back). Individual contrast images were used for group-level analyses. Finally, we used the ArtRepair Software[13]. (https://www.nitrc.org/projects/art_repair/) Outlier Detection algorithm to identify participants with scans displaying activity which deviated from the remaining sample. Three subjects (i.e., 2.4% of the data) were excluded in this step.

Accuracy during the scan, i.e., percentage of correct responses, was evaluated to exclude those participants whose performance was not statistically different from chance level and thus they were likely to not have been optimally engaged on the task during the experiment. We computed asymptotic binomial [B(n=54, p=0.25)] confidence (CI) interval at 90% confidence level (CI_{90} = 0.15 – 0.35). Accuracy cut-off was set at 35% for the 2-back task, namely the upper limit of the CI. Six participants performing poorer than 35% were thus excluded. No participant performed below 35% at 1-back. Further analyses were conducted on this sample encompassing 124 healthy participants.

Effects of condition at individual subject level were evaluated using a box car model convolved with the hemodynamic response function at each voxel, and a high-pass filter of 128 sec. Linear contrasts were computed producing a t statistical map for the 1- and 2-Back conditions, assuming the 0-Back condition as baseline. In order to further control for movement and acquisition artifacts, we calculated a bad volume regressor (BVR), using the Bad Volume function included in ArtRepair. For each subject, volumes with artifacts, detected based on scan-to-scan motion > 1mm/TR (>1 degree) and outliers relative to the global mean signal (> 5 standard deviations from global mean) were identified and included in BVR. In addition, a 24-parameter autoregressive model (Friston-24) was calculated, including current and past position parameters, along with the square of each parameter. Both BVR and Friston-24 were included as regressors in the individual first-level statistic.

Association of the Polygenic Co-expression Index with brain activity and behavior during working memory performance

Individual contrast images were used for regression analysis at the group level. We used a repeated measures ANCOVA design with LOAD as within-subjects factor and the genetic score as a regressor of interest. We also modeled the factor gender and included age and score on Edinburgh Inventory as covariates of no interest. We tested the main effect of the genetic score and the interaction of the genetic score with LOAD. We masked results selecting the voxels in which the activity was higher during working memory than the baseline. To this end, we computed one-sample t-tests on the whole sample of participants to discover brain regions in which activity was significantly greater during both 1-back and 2-back conditions compared to 0-back baseline (whole-brain $\alpha=0.05$ FWE-corrected, null conjunction analysis). MNI coordinates of statistical maxima of activation were converted to conform to the standard space of Talairach using the Nonlinear Yale MNI to Talairach Conversion Algorithm (http://noodle.med.yale.edu/~papad/mni2tal). Anatomical

localizations were determined using the Talairach Daemon software (http://www.talairach.org/daemon.html).

We asked whether imaging results were robustly associated with the SNPs we found or whether they were driven by a few epistatic SNP interactions. Therefore we cross-validated the SNPs of the PCI for their effect on imaging phenotypes. We obtained 8 new PCIs each including 7 SNPs weighted by association with the *ME* after exclusion of 1 SNP at each cycle. We then tested the effect of these PCIs on imaging phenotypes. Additionally, we modeled the effects of DRD2 rs1076560 genotype to rule out that the effect of the PCI was mediated by other genetic factors.

Finally, we investigated behavioral performance (accuracy and reaction time) during the WM task in the scanning session. We removed from this analysis two participants with RT<150 ms either in the 1-back or 2-back conditions because such timing could only be achieved by means of preemptive button presses. The final sample for the behavioral analysis included 122 participants. In the second healthy fMRI sample, participants scoring below 70% in the 2-back task were excluded by protocol. Thus, the behavioral variability in this sample was not comparable with that of the first sample, and we did not replicate the behavioral analysis.

Imaging replication in healthy subjects

Participants. Exclusion criteria included history of psychiatric and neurologic disorders and familial risk for psychiatric disorders (none of the subjects had a first-degree relative with a schizophrenia spectrum disorder or received psychotropic pharmacological treatment), history of head trauma, and history of alcohol or drug abuse. All participants had WAIS IQ≥80. All participants provided written informed consent for a protocol approved by the NIMH Institutional Review Board. Standard methods to extract DNA from white blood cells with the Puregene purification kit (Gentra Systems; Minneapolis, MN, USA) were used. Genotypes for the eight SNPs selected for the computation of the PCI were genotyped using several different Illumina BeadChips

(550K/610K/660K/2.5M), which were designed, manufactured and completed by Illumina (San Diego, CA, USA).

fMRI data acquisition and analysis. BOLD fMRI data were acquired on a 3T GE Signa Scanner (Milwaukee, WI) using a gradient-echo echo planar imaging sequence (TR=2000 ms, TE=30 ms, flip angle=90°, field of view=24 cm, matrix=64x64, 24 6mm thick slices). Individual linear contrast images of the 2-back > 0-back conditions entered in a second-level group analysis. Since 1-back assessments were not available for all subjects we focused on 2-back. The fMRI images were processed following standard procedures in SPM8 (http://www.fil.ion.ucl.ac.uk/spm). The fMRI images were first co-registered to high-resolution anatomical images and analyzed using Statistical Parametric Mapping 8 (SPM8; http://www.fil.ion.ucl.ac.uk/spm). The data were corrected for head motion artifacts, excluded if motion exceeded 2 mm in translation or 1.5° in rotation (with motion parameters used as covariates of no interest in first level analysis), spatially normalized to a $3 \times 3 \times 3$ mm^3 voxel size into a standard stereotactic space (MNI template) using affine and nonlinear transformation, and then smoothed with an 8mm full-width at half-maximum Gaussian filter. The processed images were analyzed in a two-level procedure. At the first level, separate general linear models were specified for each subject by modeling the alternating task conditions as a box car reference vector that was convolved with the SPM8 standard hemodynamic response function at each voxel. Residual movement was added as a nuisance variable. Data quality was further assessed based on time series signal-to-noise ratio, signal variance, and artifacts like ghosting at each stage of initial analysis. Second-level results were masked for activity at 2-back.

Association of the Polygenic Co-expression Index (PCI) with BOLD response during working memory performance. The effect of the PCI on BOLD response was tested using robust regression. The model included activation estimates in the clusters obtained through the analysis in

the first healthy sample with the same nuisance variables. The PCI was the independent variable. We tested the positive regression slope following the results of the main experiment. To this aim, we used a ROI approach with the clusters positively associated with the PCI in the first healthy sample and corrected for multiple comparisons by means of FDR[14] (four tests corresponding to the four clusters detected). We extracted parameter estimates from these ROIs and considered results significant at a statistical threshold of FDR-corrected $\alpha < .05$ (one-tailed).

Imaging study in patients with schizophrenia

All patients had pre-morbid T.I.B. IQ ≥ 90 (mean ± standard deviation (SD): 106 ±7.0; range 92.0–119.2) and were on stable antipsychotic treatment (mean of Gardner equivalents dose ± SD: 680 ± 272; range: 150–1500). We used PANSS to evaluate symptoms severity (mean ± SD: 61.3 ± 20.5; range: 15–104). We report results obtained with the same model used for healthy controls and also with a model in which symptoms severity and equivalents of chlorpromazine were added as nuisance variables in the general linear model.

PHARMACOGENETIC STUDY

Participants and clinical protocols

Supplementary Table 2 reports clinical data of the two samples of patients with schizophrenia. In the first clinical sample, patients had not received any psychotropic medication, including benzodiazepines, antidepressants, or mood stabilizers, for at least 1 week (1 month for patients receiving depot medication prior to study inclusion). All patients received olanzapine monotherapy for 8 weeks (mean olanzapine dose ± SD: 20.1±7.3 mg). Symptoms were assessed at study entry (day 0) and at day 56 with the Positive And Negative Syndrome Scale (PANSS) by only one trained psychiatrist (GC), who was blind to genotypes (see Blasi et al.[15] for further details). We obtained the genotypes of the eight SNPs of the PCI from blood samples of the patients using

TaqMan® SNP genotyping. DNA was isolated from peripheral blood samples through a QIAamp DNA Blood Maxi Kit (Qiagen, Venlo, NE). About 10 ml of fresh blood were used to this aim. We used about 20 ng of DNA through an allele-specific TaqMan® SNP Genotyping Assay (Life Technologies, Carlsbad, CA, USA) according to the manufacturer's instructions as reported in Pergola et al.[9].

The second cohort consisted of 40 patients with schizophrenia with history of inadequate treatment response. All these patients were Caucasians of European ancestry and were diagnosed by structured clinical interview with chronic schizophrenia using DSM-IV criteria. All individuals volunteered to participate in a double-blind, placebo-controlled cross-over study with standard doses of atypical antipsychotics[16]. The sequence of placebo and antipsychotic treatment was randomized across patients and each lasted 1-4 weeks. DNA samples were genotyped using IlluminaHumanHap550K/610Quad Bead Chips, according to the manufacturer's protocol. After QC procedures[17], 495 089 high-quality autosomal SNPs were available for analysis.

Supplementary Table 2 about here

Statistical procedures

We assessed treatment improvement variables statistical distribution with Shapiro-Wilk test, which turned out significant, i.e., the variables were not normally distributed (first cohort: $W= 0.96$, $p = 0.07$; second cohort: $W=0.9$, $p < 0.05$). In the association study, we used Spearman's Rho correlation index to associate PANSS total scores and PCI. The PCI of the two cohorts was computed based on genotypes assessed as above reported. In the second clinical cohort, based on the results from the first cohort, we tested to what extent large PCI values predicted greater improvement using Spearman's Rho correlation index (one-tailed probabilities).

In the predictive model, we categorized patients in responders and non-responders. We used 20% symptom improvement as a cut-off [18, 19]. We tested the PCI, the treatment dose, and off-medication symptom severity to predict membership of individual patients to response category. We used ROC curves to estimate sensitivity and specificity and compute the area under the curve (AUC) as an index of the difference between the predictors and chance level (non-parametric test, SPSS). Since sub-sampling the cohorts in the prediction study substantially reduced statistical power, we computed ROC curves on a pooled sample including both cohorts. We marginalized the effect of cohort by identifying variables showing significant changes between the first and the second cohort by means of t-tests and then computed the residuals of these variables using one-way ANOVAs.

2. SI RESULTS

WGCNA

We detected 67 sets of co-expressed genes. 2097 genes out of the 23636 were not clustered in any gene set. We computed the module eigengene (*ME*), i.e., the first principal component, of all genes' expression in each gene set. The median gene set size was 170 (range: 46 – 2 452) and the median variance explained by the MEs was 36.2% (range: 24.0% – 62.4%).

SNP ASSOCIATION STUDY AND CO-EXPRESSION POLYGENIC INDEX COMPUTATION

We pooled minor allele carriers for rs6504631 and rs12940715 (minor homozygous respective sample sizes were n=4 and n=3) and we excluded rs6504631 (p-value > 0.05 after pooling subjects). The first principal component and each allelic subpopulation complied with the normal distribution (Shapiro-Wilk test for normality, all p-values > 0.05). By definition of the PCI, participants with greater PCI scores also had greater co-expression levels. Notably, the linear fit between the PCI and the leave-one-out cross-validated PCI was nearly perfect ($t_{198} = 116$, $R^2 = 0.99$, $p = 3.2\times10^{-183}$; Supplementary Figure 2), suggesting that the association between expression of the gene set and the PCI was not driven by a few influential observations. The leave-one-out cross-validated PCI was associated with expression of the gene set (*ME*, $t_{198} = -9.4$, $R^2 = 0.31$, $p = 1.6\times10^{17}$; Supplementary Figure 2) and D2L transcriptional levels in the left-out subjects ($t_{198} = -5.2$, $R^2 = 0.12$, p-value $= 4.5\times10^{-7}$). In the leave-one-out cross validation the weights of the SNPs were computed on N-1 subjects and were applied to the Nth subject. Therefore, this measure reflects the power of the PCI to approximate D2L gene set co-expression levels in subjects independent of the training dataset. Leave-k-out cross-validation showed that cross-validated PCI was still associated to the *ME* when the training set was about half (N = 100) of the whole sample (R^2 mean

± SD of 10 000 random permutations = 0.28 ± 0.05, see Supplementary Figure 2). K-fold cross-validation supported these results, with PCI explaining on average 32% of *ME* variance (Supplementary Figure 2).

The *ME* was not associated with ethnicity (p = 0.9), and the PCI by ethnicity interaction was not significant (p = 0.9). When tested independently, the association between the *ME* and the PCI was comparable between Caucasian (r = 0.69) and African-American individuals (r = 0.53).

We tested the robustness of the SNP selection with three analyses: i. we cross-validated the SNP selection (keeping the original priority pruning) and tested the association between the *ME* and the PCI computed using the first eight SNPs for each cycle of the cross-validation. We found a strong correlation between the two variables (R^2 = .38, Supplementary Figure 1); ii. we ranked the genes by their most associated gene and compared the ranking with that resulting from 100 random-seed LD pruning. The rankings correlated positively (median r = .35) and were extremely different from the null distribution (Cohen's d = 2.5); iii., we investigated the correlations between the *ME* and the PCI as a function of the number of SNPs included in the polygenic index. Supplementary Figure 3a represents the correlation of a series of PCIs with the *ME* and with *DRD2* expression. Each point indicates the Pearson's correlation coefficient for a cross validated PCI including a given number of SNPs (represented on the x-axis and varying between 1 and 100). With few SNPs, the correlation increases steeply with the inclusion of more markers, but plateaus around 20 SNPs, i.e., there is no effective information increase. This is more evident in Supplementary Figure 3b, which plots the percent correlation variation as a function of the number of SNPs included in the PCI. Here, the difference between 8 and 9 SNPs is the last variation > 5%. Therefore, including in the PCI a number of SNPs greater than 9 causes no relevant increase in the association between the PCI and the *ME*. This is exactly the number of SNPs selected for the PCI (one of them was excluded because expression was not normally distributed within all genotypic populations).

These validation steps, together with the findings on the functional role of the SNPs reported in the SI Appendix, support the idea that the PCI we used, which included 8 SNPs, is not biased by overfitting. In further support of these results, Supplementary Figure 4 represents the replication obtained in BrainEAC with variable RNA quality cut-offs. The monotonic increase in the variance explained by the PCI suggests that with increasing quality of the observations the relationship between the PCI and the principal component of D2L expression gene set increased.

Supplementary Figure 1 about here

Supplementary Figure 2 about here

Supplementary Figure 3 about here

Supplementary Figure 4 about here

Association of the Polygenic Co-expression Index with genomic principal components

Supplementary Table 3 shows the variance explained by each PCs extracted, Spearman's Rho and correlation p-values with the *ME* and D2L expression. We did not find a significant association between the PCs and either variable.

Supplementary Table 3 about here

Association of the Polygenic Co-expression Index with age

After preprocessing, none of the eight module genes proximal to the SNPs in the PCI was significantly associated with age (all $p > .35$). We investigated whether our PCI predicted the *ME* even when we restricted age range to the [15,55] years interval. We chose this interval because it is the age interval represented in the fMRI, behavioral, and clinical studies. We found that the R^2 of

the association was basically unaffected (Braincloud: all samples, R^2 = .38, restricted age range, R^2 = .35; BrainEAC: all samples with RIN > 6, R^2 = .14, restricted age range R^2 = .17).

IMAGING STUDY

Supplementary fMRI analyses in healthy individuals

In the fMRI analysis reported in the main text we adopted a within-subject design to associate the PCI with WM activity. To rule out potential bias introduced by specific statistical models, we additionally computed the average between 1-back and 2-back activations, correlated this activity with the PCI and reported the activations at the same statistical threshold of the within-subject analysis. Results are largely overlapping (Supplementary Figure 5), for example, here the "A" cluster reported in Table 3 has cluster extent = 76, Z = 4.05, and Bonferroni-corrected p = .047. This result supports the robustness of the findings.

Supplementary Figure 5 about here

In previous studies, we have reported the role of a genetic variant of *DRD2* (rs1076560) in schizophrenia. Here, we tested the independence of the genetic variants investigated in these studies from *DRD2* rs1076560. Thus, we performed an additional imaging analysis in the first healthy sample in which we included *DRD2* rs1076560 genotype as an additional factor. Results of the PCI on brain activity were substantially unaffected. Importantly, there was no interaction between the PCI and *DRD2* rs1076560. Results are reported at the same statistical threshold of the main PCI analysis in the first healthy sample (Supplementary Figure 6).

Supplementary Figure 6 about here

Finally, we computed eight additional PCIs by removing one SNP per time and tested the effect of these cross-validated PCIs on brain activity (results in Supplementary Table 4). Results are consistent with those of the main PCI, showing that the imaging findings are not driven but just one SNP.

Supplementary Table 4 about here

Association between PCI and brain activity during working memory in patients with schizophrenia

Supplementary Figure 7 reports PCI effects on brain activity during WM in patients with schizophrenia. In this sample, prefrontal as well as parietal activity appear robustly associated with PCI. Results are reported at the same statistical threshold of the main PCI analysis in the first healthy sample.

Supplementary Figure 7 about here

In addition, to test the independence of this result from symptomatology and medical treatment, we computed a model in which we included Gardner equivalents and PANSS total score as regressors. Results are largely overlapping, describing a robust association of PCI with prefrontal (x, y, z= 1, 30, 59; k=109; Z=4.37; x, y ,z= 61, 16, 6; k=44; Z=3.92) and parietal (x, y, z= 38, -56, 59; k=100; Z=4.30; x, y, z= -40, -48, 51; k=42; Z=3.57) clusters.

Complete statistics of the behavioral analysis reported in the main text

We performed repeated measures ANCOVAs (within-subjects factor LOAD [1-back, 2-back]) on WM accuracy, reaction times, and on an efficiency index (accuracy over reaction times).

The latter analysis is reported for exploratory purposes. The analysis on accuracy yielded a significant main effect of LOAD ($F_{1,120}$=43, p<.001). There was no significant effect of PCI ($F_{1,120}$=0.38, p=.54) and no significant LOAD×PCI interaction ($F_{1,120}$=.11, p=.74). The analysis on reaction times yielded a significant LOAD×PCI interaction, surviving Bonferroni correction (two tests [accuracy and reaction times]; α = .025; $F_{1,120}$=6.9, p=.01). Supplementary Figure 8 plots the relationship between reaction time at 2-back and PCI. The main effects of LOAD ($F_{1,120}$=2.5, p=.12) and PCI ($F_{1,120}$=1.9, p=.18) were not significant. Post-hoc regressions computed to resolve the significant interaction were non-significant for 1-back (t_{120}=.12, adjusted R^2=-.008, p=.91) but yielded a significant fit for 2-back (t_{120}=2.3, adjusted R^2=.033, p=.024). Greater D2L PCI was related with longer reaction times at 2-back. The analysis on efficiency yielded a significant LOAD×PCI interaction, ($F_{1,120}$=4.4, p=.037). The main effects of LOAD ($F_{1,120}$=.34, p=.56) and PCI ($F_{1,120}$=2.8, p=.13) were not significant. Post-hoc regressions computed to resolve the significant interaction were non-significant for 1-back (t_{120}=.40, adjusted R^2=-.007, p=.69) but yielded a significant fit for 2-back (t_{120}=2.3, adjusted R^2=.035, p=.023). Greater D2L PCI was related with lower efficiency at 2-back.

Supplementary Figure 8 about here

PHARMACOGENETIC STUDY

We found that the cohorts differed significantly for treatment dose (in chlorpromazine equivalents; t_{84} = 5.0, p < .001) and off-medication symptoms (t_{84} = 8.6, p < .001). Therefore, we marginalized these variables by the factor *cohort*. Supplementary Figures 8 and 9 show the pharmacogenetics results.

Supplementary Figure 9 about here

Supplementary Figure 10 about here

3. SI DISCUSSION

Beside rs2486064, another SNP included in our PCI is related to the GSK3β pathway: rs851436 is located downstream of *OSR1*, which codes for a transcription factor that suppresses the expression of cytoplasmic β-catenin[20]. β-catenin has been involved in mechanisms of signaling transduction within the D2R cAMP-independent pathway[21]. Also interesting in a neurogenetic perspective is the Sdk2 neuronal adhesion molecule in which rs12940715 is located. In particular, Sdk2 binds proteins involved in recruitment of transmembrane molecules, such as Post-Synaptic Density proteins including PSD-95, which binds neurotransmitter receptors. Notably, the post-synaptic density has been considered a critical cellular district for the pathogenesis of schizophrenia[22].

In summary, the co-eQTLs identified in the present study are located in genetic regions associated with cAMP-independent D2R signaling, synaptic function, response to stress, and learning. We successfully replicated the findings in independent datasets, which suggests that these co-eQTLs play a critical role in determining inter-individual variability associated with *DRD2*-dependent phenotypes. Furthermore, imaging results were not affected when we cross-validated SNPs for the computation of the PCI, suggesting that the eight SNPs detected independently modulate molecular and imaging phenotypes.

Considerations on the pharmacogenetics studies reported

Medication compliance and drug abuse may play a major role in clinical outcome and/or brain activity, and may share genetic risk factors with schizophrenia. To partially control for medication compliance, in the clinical protocol of the discovery study we included multiple intermediate clinical assessments at days 7, 14, and 28 of treatment. On the other hand, the clinical

protocol of the NIH sample was performed while patients were admitted to the hospital. Therefore, in both samples medication compliance is less likely to exert a major confounding role. We also collected information on drug abuse in patients and controls by means of semi-structured interviews, although we have no biochemical evidence of abstinence from drugs available.

Treatment response was generally lower in the replication dataset for several reasons. First, these patients entered the protocol following history of inadequate treatment response, whereas the first clinical included patients who were drug-free or drug-naïve at baseline. Second, treatment duration in the second clinical sample varied between 1 and 6 weeks, while it was always 8 weeks in the first. Third, antipsychotics varied across patients in the second clinical sample, whereas all patients in the first clinical sample were treated with olanzapine in monotherapy. The discrepancy between datasets adds support to the conclusion that the PCI is associated with treatment response, regardless of details of the protocol.

4. SI FIGURES LEGENDS

Supplementary Figure 1- Results of the co-eQTL study

In all panels, the solid line represents the regression fit and the dotted lines represent 95% confidence intervals for the regression.

panel a: Scatterplot of the relationship between the co-expression of the whole gene-set (Module Eigengene, *ME*) and D2L expression.

panel b: Scatterplot of the *ME* as a function of the Polygenic Co-expression Index (PCI).

panel c: Scatterplot of D2L expression as a function of the PCI.

Supplementary Figure 2- Cross-validation of the PCI

panel a: Leave-one-out cross-validated PCI on the X-axis, full-sample PCI on the Y-axis.

panel b: Fit between the leave-one-out cross-validated PCI and the *ME*.

panel c: K-fold cross-validation. The dataset was split in 10 random subsamples. In this non-exhaustive method each observation was taken into account only once. Each dot represents a single subsample. The plot shows R^2 values of the fit between the PCI and the *ME*. The average R^2 is close to the median estimate obtained through leave-k-out cross validation (panel d).

panel d: Leave-k-out cross-validation. Each box represents 10 000 permutations. The PCI was computed based on data from different training samples (size indicated on the X-axis). The association with the *ME* was permuted to obtain median and dispersion estimates for the boxplot. R^2 values increase with increasing training information, but remain stable with smaller training samples. Abbreviations: PCI, Polygenic Co-expression Index.

Supplementary Figure 3- Correlation analysis of the cross-validated PCI

a. The scatterplot illustrates the correlation between a series of PCIs computed with variable numbers of SNPs and two dependent variables (the *ME* and *DRD2* expression). b. The plot displays

the percent variation of the correlation between the *ME* and a series of PCIs computed with variable numbers of SNPs. For the sake of clarity the percent change is limited to the first 25 PCIs (the information increase is negligible for more inclusive PCIs). To the right of the ninth PCI there is no increase > 5%.

Supplementary Figure 4- Variation of explained variance based on data quality in BrainEAC

The X-axis represents the minimum RNA integrity (RIN) of samples included. The Y-axis represents the R^2 of the correlation between the Module Eigengene (*ME*) and the Polygenic Co-expression Index (PCI). The plot shows that the association between the *ME* and the PCI steadily increases with increasing RIN, i.e., with increasing data quality. At RIN > 3: N=94, R= .17, p= .047. The correlation increased at RIN > 4: N = 77, R = .20, p = .040. See the main text for statistics at other cut-offs.

Supplementary Figure 5- First healthy fMRI sample. Effect of the PCI in a between-subjects model

Significant clusters associated with the positive slope of the Polygenic Co-expression Index (PCI) in a factorial model with the between-subjects factor gender and age and handedness as covariates of no interest. Results are displayed at the same statistical threshold of the repeated measures analysis reported in the main text. Left in the figure is left in the brain.

Supplementary Figure 6- First healthy fMRI sample. Effect of the PCI co-varied for *DRD2* rs1076560 genotypes

Significant clusters associated with the positive slope of the Polygenic Co-expression Index (PCI) in a repeated measures ANCOVA model with the within-subjects factor LOAD, between-subjects factors gender and *DRD2* rs1076560 genotypes (GG; T carrier), and age and handedness as

covariates of no interest. Results are reported at the same statistical threshold of the main PCI analysis in the first sample. Left in the figure is left in the brain.

Supplementary Figure 7- Clinical fMRI sample. Association between PCI and brain activity during WM in patients with schizophrenia

Significant clusters associated with the positive slope of the Polygenic Co-expression Index (PCI) in the same model reported for the healthy controls in the first sample, in a sample of patients with schizophrenia. Results are reported at the same statistical threshold of the main PCI analysis in the first sample. Left in the figure is left in the brain.

Supplementary Figure 8- First healthy fMRI sample. Fit between PCI and Reaction Time at 2-back during WM

The solid line represents the linear trend. Abbreviations: PCI, Polygenic Co-expression Index.

Supplementary Figure 9- Scatterplots of the fits between PCI and treatment response

The Y-axis reports treatment response values computed as the difference in total PANSS score between the no-drug and drug conditions. Scatterplots: the X-axis reports raw values of the Polygenic Co-expression Index (PCI). The solid lines represent linear regression trend lines. Boxplots: the X-axis reports the quartile of the PCI from lower (quartile 1) to higher values (quartile 4). Spearman's Rho correlation is significant for both samples. Left panel: first clinical sample. Right panel: second clinical sample.

Supplementary Figure 10- Prediction of treatment response based on the PCI, treatment dose, and off-medication symptom severity

ROC curves in the pooled sample of patients with schizophrenia. The central diagonal line represents chance level. Here, the following legend applies: red line: PCI; green line: treatment dose; blue line: off-medication symptoms; purple line: reference line. Abbreviations: PCI, Polygenic Co-expression Index; ROC, receiver operating characteristic.

5. SI REFERENCES

1. Schroeder A, Mueller O, Stocker S, Salowsky R, Leiber M, Gassmann M, *et al.* The RIN: an RNA integrity number for assigning integrity values to RNA measurements. *BMC Mol Biol* 2006;**7**:3.

2. Colantuoni C, Lipska BK, Ye T, Hyde TM, Tao R, Leek JT, *et al.* Temporal dynamics and genetic control of transcription in the human prefrontal cortex. *Nature* 2011;**478**(7370):519-23.

3. Consortium SWGotPG. Biological insights from 108 schizophrenia-associated genetic loci. *Nature* 2014;**511**(7510):421-7.

4. Roussos P, Katsel P, Davis KL, Siever LJ, Haroutunian V. A system-level transcriptomic analysis of schizophrenia using postmortem brain tissue samples. *Arch Gen Psychiatry* 2012;**69**(12):1205-13.

5. Iwamoto K, Bundo M, Ueda J, Kato T. Expression of ribosomal subunit genes increased coordinately with postmortem interval in human brain. *Mol Psychiatry* 2006;**11**(12):1067-9.

6. Beasley TM, Erickson S, Allison DB. Rank-based inverse normal transformations are increasingly used, but are they merited? *Behav Genet* 2009;**39**(5):580-95.

7. Zhang B, Horvath S. A general framework for weighted gene co-expression network analysis. *Stat Appl Genet Mol Biol* 2005;**4**:Article17.

8. Rhead B, Karolchik D, Kuhn RM, Hinrichs AS, Zweig AS, Fujita PA, *et al*. The UCSC Genome Browser database: update 2010. *Nucleic Acids Res* 2010;**38**(Database issue):D613-9.

9. Pergola G, Di Carlo P, Andriola I, Gelao B, Torretta S, Attrotto MT, *et al.* Combined effect of genetic variants in the GluN2B coding gene (GRIN2B) on prefrontal function during working memory performance. *Psychol Med* 2015:1-16.

10. Maher BS. Polygenic Scores in Epidemiology: Risk Prediction, Etiology, and Clinical Utility. *Curr Epidemiol Rep* 2015;**2**(4):239-44.

11. Loh PR, Tucker G, Bulik-Sullivan BK, Vilhjalmsson BJ, Finucane HK, Salem RM, *et al.* Efficient Bayesian mixed-model analysis increases association power in large cohorts. *Nat Genet* 2015;**47**(3):284-90.

12. Oldfield RC. The assessment and analysis of handedness: the Edinburgh inventory. Neuropsychologia 1971;**9**(1):97-113.

13. Mazaika PK W-GS, Reiss AL. Artifact Repair for fMRI data from High Motion Clinical Subjects. Annual Meeting og the Organization for Human Brain Mapping, Chicago, 2007.

14. Benjamini Y, Krieger AM, Yekutieli D. Adaptive linear step-up procedures that control the false discovery rate. *Biometrika* 2006;**93**(3):491-507.

15. Blasi G, Selvaggi P, Fazio L, Antonucci LA, Taurisano P, Masellis R, *et al.* Variation in Dopamine D2 and Serotonin 5-HT2A Receptor Genes is Associated with Working Memory Processing and Response to Treatment with Antipsychotics. *Neuropsychopharmacology* 2015;**40**(7):1600-8.

16. Apud JA, Zhang F, Decot H, Bigos KL, Weinberger DR. Genetic variation in KCNH2 associated with expression in the brain of a unique hERG isoform modulates treatment response in patients with schizophrenia. *Am J Psychiatry* 2012;**169**(7):725-34.

17. Dickinson D, Straub RE, Trampush JW, Gao Y, Feng N, Xie B, *et al.* Differential effects of common variants in SCN2A on general cognitive ability, brain physiology, and messenger RNA expression in schizophrenia cases and control individuals. *JAMA psychiatry* 2014;**71**(6):647-56.

18. Correll CU, Kishimoto T, Nielsen J, Kane JM. Quantifying clinical relevance in the treatment of schizophrenia. *Clin Ther* 2011;**33**(12):B16-39.

19. Kane J, Honigfeld G, Singer J, Meltzer H. Clozapine for the treatment-resistant schizophrenic. A double-blind comparison with chlorpromazine. *Arch Gen Psychiatry* 1988;**45**(9):789-96.

20. Otani K, Dong Y, Li X, Lu J, Zhang N, Xu L, *et al.* Odd-skipped related 1 is a novel tumour suppressor gene and a potential prognostic biomarker in gastric cancer. *J Pathol* 2014;**234**(3):302-15.

21. Beaulieu JM, Gainetdinov RR, Caron MG. The Akt-GSK-3 signaling cascade in the actions of dopamine. *Trends Pharmacol Sci* 2007;**28**(4):166-72.

22. Focking M, Lopez LM, English JA, Dicker P, Wolff A, Brindley E, et al. Proteomic and genomic evidence implicates the postsynaptic density in schizophrenia. *Mol Psychiatry* 2015;**20**(4):424-32.

6. SI TABLES

Supplementary Table 1.Genes of the D2L gene set and match with BrainEAC.

OligoID	Gene	Probe	kTotal	kWithin	kOut	kDiff	KWithin_scaled	KTotal_scaled
hHA034464	IGSF1	4021811	12.65456915	3.240113376	9.414455774	-6.174342398	1	0.017147694
hHA034560	TTN	2589741	11.42777714	2.906967666	8.52080947	-5.613841804	0.897180848	0.015485318
hHC022740	CLDN4	t3007960	27.18653434	2.852773841	24.33376049	-21.48098665	0.880454944	0.036839371
hHA039264	GATAD2A	3825755	19.82223941	2.819066022	17.00317339	-14.18410736	0.87005166	0.026860313
hHA033312	CHIA	t2351763	14.84847467	2.589927214	12.25854746	-9.668620246	0.79933228	0.020120566
hHC025044	SDK2	3770116	36.49585122	2.493919312	34.00193191	-31.50801259	0.769701249	0.049454049
hHR025236	OR2S2	3205020	11.18905117	2.466598052	8.72245312	-6.255855067	0.761269056	0.01516183
hHA039456	NEURL4	3743686	14.22811587	2.435825366	11.79229051	-9.356465142	0.751771646	0.019279943
hHR028896	DEFB108B		31.12107164	2.415115978	28.70595567	-26.29083969	0.745380083	0.042170903
hHA034272	MAP4	2673022	18.82160096	2.290084678	16.53151628	-14.2414316	0.706791526	0.025504389
hHA034368	ARSB	2863991	10.97742594	2.261872955	8.715552989	-6.453680034	0.698084509	0.014875065
hHA040704	DAZAP1	3815860	18.04073548	2.192443615	15.84829187	-13.65584825	0.67665645	0.02444627
hHA036180	ING1	3501472	46.92208853	2.183411562	44.73867697	-42.55526541	0.673868877	0.063582221
hHC020928	PTPN21	3575305	15.96497736	2.124175647	13.84080171	-11.71662606	0.655586827	0.021633494
hHA034944	RBM6	2622369	29.15528517	2.052361585	27.10292358	-25.050562	0.633422769	0.039507146
hHC031860	FLJ35390	2999701	15.07901802	2.048859697	13.03015833	-10.98129863	0.632341977	0.020432966
hHC019296	HSD3B2	2354449	12.08243984	2.036502699	10.04593714	-8.009434437	0.628528222	0.016372425
hHR019572	PRM3	3680252	11.08934469	2.02999533	9.059349357	-7.029354027	0.626519845	0.015026722
hHC016212	RHO	2641782	12.03194789	1.99683183	10.03511606	-8.038284227	0.616284555	0.016304005
hHC016320	ACTRT2	2316917	23.57957938	1.941835582	21.6377438	-19.69590822	0.599310998	0.031951733
hHA036672	GALNT10	2836589	7.676908275	1.938759576	5.738148699	-3.799389122	0.598361647	0.010402667
hHA035988	CNR1	2963865	18.58085758	1.919638074	16.6612195	-14.74158143	0.592460155	0.025178167
hHC016224	GLI1	3418142	7.872563201	1.91523157	5.957331631	-4.042100061	0.591100171	0.010667791
hHA035508	USH2A	2455771	11.89097108	1.869528254	10.02144283	-8.151914576	0.576994703	0.016112973
hHA038304	SYNE2	3539780	7.905478221	1.835610111	6.06986811	-4.234257999	0.566526506	0.010712393
hHA035904	DNAH9	3710610	6.28823015	1.833861492	4.454368659	-2.620507167	0.565986828	0.008520926
hHC022944	BTG4	t3390949	6.790689289	1.821073393	4.969615896	-3.148542502	0.562040022	0.009201788
hHA040404	JPH2	3906791	8.290062662	1.812701313	6.477361349	-4.664660036	0.559456137	0.011233528
hHA035616	DRD2	3391660	9.324910505	1.78065351	7.544256995	-5.763603485	0.549565186	0.012635808
hHA033396	TNXB	t2949622	18.71843561	1.758332441	16.96010317	-15.20177073	0.542676208	0.025364594
hHA038868	HS6ST2	t4022183	10.20541729	1.757217266	8.448200027	-6.690982761	0.54233203	0.013828947
hHR018036	ZSCAN23	t2947348	10.70277695	1.750437092	8.952339855	-7.201902763	0.540239457	0.014502899
hHC016500	LHX9	2373713	9.833472379	1.742571326	8.090901053	-6.348329727	0.537811837	0.01332494
hHC019776	NCAPG	2720279	14.69914851	1.688365547	13.01078296	-11.32241741	0.521082243	0.019918221
hHC021396	NAT8B	2559639	7.601852414	1.687344161	5.914508253	-4.227164092	0.520767012	0.010300962
hHR015060	HIST1H3G	2946370	9.538219437	1.572735699	7.965483737	-6.392748038	0.485395268	0.012924855

hHC016032	CALHM3	3304798	18.75498212	1.56428362	17.1906985	-15.62641488	0.482786692	0.025414116
hHC022368	NTRK1	2361790	21.50127204	1.534741406	19.96653063	-18.43178923	0.473669044	0.029135503
hHA039552	TTN	2589534	6.383884061	1.476872824	4.907011237	-3.430138413	0.455808996	0.008650543
hHA036576	TSPAN17	2842712	22.34299933	1.396427574	20.94657175	-19.55014418	0.430981084	0.030276093
hHC026784	KRTAP9-3	3721256	54.73639757	1.347993998	53.38840357	-52.04040957	0.416032972	0.074171075
hHR020904	LBX2	2560179	14.30172744	1.324562267	12.97716517	-11.65260291	0.40880121	0.019379691
hHA036768	NCAPH2	t3950872	14.9911103	1.321733822	13.66937648	-12.34764266	0.407928263	0.020313846
hHA034644	TTN	2589683	9.609833126	1.256914066	8.35291906	-7.096004994	0.387922866	0.013021896
hHC021300	AGR2	t3039791	5.967740641	1.230874861	4.73686578	-3.505990919	0.379886355	0.008086644
hHR014304	IL31	3475500	15.32798281	1.229434166	14.09854864	-12.86911448	0.379441712	0.020770328
hHC022560	BSND	2337399	7.006712782	1.208240221	5.798472561	-4.59023234	0.3729006	0.009494513
hHC025152	TIGD1	2603900	14.76577505	1.17261003	13.59316502	-12.42055499	0.361904012	0.020008504
hHA040020	LTBP1	t2476510	14.79293307	1.112423742	13.68050933	-12.56808559	0.343328647	0.020045304
hHA035604	PTPN7	2451184	20.10725435	1.106378862	19.00087549	-17.89449663	0.341463009	0.027246526
hHC013440	DHX33	3742728	11.49653062	1.06438018	10.43215044	-9.367770265	0.328500906	0.015578483
hHC030624	AMAC1L2		13.27933513	1.044660915	12.23467421	-11.1900133	0.322414926	0.017994289
hHC020532	CPLP	3820308	5.865702074	1.016877895	4.848824179	-3.831946284	0.31384022	0.007948375
hHA034656	EFCAB6	3963086	15.70244082	0.997597201	14.70484362	-13.70724641	0.307889597	0.021277741
hHC028608	PCBD2	2829615	25.24478916	0.969087267	24.2757019	-23.30661463	0.299090542	0.034208191
hHA034164	RNF128	3986265	9.505540838	0.942612945	8.562927892	-7.620314947	0.290919741	0.012880573
hHR016704	HIST1H1E	t2899171	5.043667411	0.915383799	4.128283611	-3.212899812	0.282515978	0.006834469
hHC022728	CHIT1	2451620	23.93977015	0.838201139	23.10156901	-22.26336787	0.258695003	0.032439813
hHC019667	ALDH3A1	t3748957	10.14912922	0.819624218	9.329505002	-8.509880784	0.252961586	0.013752674
hHR014975	ACR	3951122	8.747026183	0.811612273	7.93541391	-7.123801636	0.25048885	0.011852741
hHR019368	SSTR5	3643566	20.9984474	0.794616515	20.20383089	-19.40921437	0.245243429	0.028454145
hHA035796	DHX9	2371006	4.673911037	0.774850404	3.899060633	-3.124210229	0.239142991	0.006333427
hHR031476	TAS2R42		7.673022498	0.773693428	6.89932907	-6.125635642	0.238785912	0.010397402
hHA039900	BTN3A1	2899373	17.97047092	0.740990985	17.22947994	-16.48848895	0.228692919	0.024351057
hHA038100	WDR4	3933823	13.94452746	0.714338424	13.23018904	-12.51585061	0.220467108	0.018895664
hHC003732	LRRC19	3202227	3.311120188	0.693451497	2.617668691	-1.924217193	0.214020751	0.004486765
hHC011016	TNMD	3984459	10.82347674	0.671258093	10.15221865	-9.480960559	0.207171174	0.014666455
hHC011315	PPP3R2	3218211	11.88955922	0.665967858	11.22359136	-10.5576235	0.205538443	0.01611106
hHC009216	GLB1L	2600041	12.49718369	0.635925058	11.86125863	-11.22533357	0.196266298	0.016934427
hHA035892	GPLD1	2945551	5.592717157	0.62205255	4.970664607	-4.348612057	0.19198481	0.007578464
hHC019763	PLEKHA2	3094960	6.69856546	0.560502465	6.138062995	-5.57756053	0.172988535	0.009076955
hHC023424	OSR1	2542453	6.359834236	0.548618075	5.811216161	-5.262598086	0.169320641	0.008617954
hHR026976	SNORD45A	2342635	5.495609728	0.499002812	4.996606915	-4.497604103	0.154007825	0.007446878
hHA036072	GALNT10	2836607	7.985815281	0.493348542	7.492466739	-6.999118197	0.15226274	0.010821255
hHR029088	hCG_1651160		5.125501167	0.491777844	4.633723324	-4.14194548	0.151777974	0.006945359
hHC012192	POP1	3108702	17.23586045	0.48905363	16.74680682	-16.25775319	0.150937197	0.023355616
hHC020232	RHAG	2956514	4.096578323	0.476096648	3.620481674	-3.144385026	0.146938268	0.005551107
hHA037139	SLC28A1	3606016	10.24603543	0.474565306	9.771470122	-9.296904816	0.146465648	0.013883987

hHC017172	CES3	t3665029	7.004813281	0.450060188	6.554753093	-6.104692905	0.138902605	0.009491939
hHC001511	MED26	t3854032	15.94882099	0.395293392	15.5535276	-15.15823421	0.121999864	0.021611601
hHC011688	DDX4	2810045	4.73899827	0.37829557	4.3607027	-3.98240713	0.116753807	0.006421625
hHC019871	DLX4	t3726114	3.762241809	0.314649473	3.447592336	-3.132942863	0.097110637	0.005098061
hHC018911	TDRD12	3829116	5.366707487	0.274634639	5.092072848	-4.817438209	0.084760812	0.007272208
hHC016284	CACNA2D4	t3440066	3.957822076	0.254090232	3.703731844	-3.449641612	0.078420167	0.005363084
hHR027648	HIST2H2AC		4.800015392	0.235972732	4.56404266	-4.328069928	0.072828542	0.006504306

The first column reports the probe name in Braincloud. The second column reports the corresponding gene name. The third column contains the probe numbers of BrainEAC. Here, the following legend applies: green: the nucleotide sequence of the Braincloud probe completely overlaps with BrainEAC's exon-specific probe; orange: partial overlap; black: no overlap between the probes (total gene expression in BrainEAC was used in this case); red text: genes not found in BrainEAC. Probes are listed following their ranking in scaled connectivity within the gene set (KWithinScaled).

The fourth column reports whole-network connectivity for each gene (kTotal). The kTotal of the i-th gene is defined as the sum of the adjacency matrix values a_{ij} where the j-th genes encompass all network genes. The fifth column reports intra-modular connectivity (kWithin). The kWithin of the i-th gene is defined as the sum of the adjacency matrix values a_{ij} where the j-th genes encompass D2L gene-set genes. The sixth and seventh columns report extra-modular connectivity (kOut), that is merely kTotal minus kWithin and the difference between kWithin and kOut (kDiff).

The eighth and ninth columns report scaled values of kWithin and kTotal (i.e., $kWithin_i/kWithin_{MAX}$, and $kTotal_i/kTotal_{MAX}$).

Supplementary Table 2. Clinical data of the samples

Sample Name	Premorbid IQ (WRAT)	Length of illness (years)	Chlorpromazine equivalents	PANSS score at baseline	Drug-free period (months)
First clinical study	104.4±7.7	5.09± 6.08	604±231	*Total:* 105.8±21.8<br>*Positive*: 24.0±6.5<br>*Negative:*27.7±9.6<br>*General:*54.0±12.0	10.8±9.8
	**Premorbid IQ (WRAT)**	**Length of illness (years)**	**Chlorpromazine equivalents**	**PANSS score at baseline (drug)**	**PANSS score at baseline (placebo)**
Second clinical study	91.0±14	7.1±6.1	564±267	*Total:* 63±15<br>*Positive:* 14.3±4.4<br>*Negative*: 17±5.8<br>*General*: 32±7.9	*Total:* 62±16<br>*Positive:*14±3.9<br>*Negative:*16±6.5<br>*General:*31±7.8

Supplementary Table 3. Spearman correlation between genomic principal components, module eigengene, and D2L expression in Braincloud.

Genomic PCs	Variance Explained (%)	*ME*	D2L expression
PC1	9.52	-0.105 (.141)	-0.105 (.138)
PC2	0.95	0.034 (.635)	0.062 (.385)
PC3	0.62	0.022 (.758)	-0.041 (.562)
PC4	0.55	0.060 (.340)	0.068 (.343)
PC5	0.54	-0.095 (.183)	-0.070 (.326)
PC6	0.53	-0.045 (.528)	0.046 (.519)
PC7	0.53	0.121 (.089)	0.071 (.319)
PC8	0.53	-0.036 (.617)	-0.099 (.163)
PC9	0.52	0.021 (.767)	0.070 (.323)
PC10	0.52	0.045 (.527)	0.119 (.094)

The first column reports genomic PCs. The second column reports the percent variance explained by PCs. Columns 2-4 display Spearman's Rho and the related p-value in brackets. Abbreviation: PCA, principal component analysis; PC principal component; D2L, Dopamine Receptor 2 long isoform.

Supplementary Table 4. Cross-validation of SNPs in the PCI and fit with brain activity during WM.

Polygenic Co-expression Index	BA	MNI coordinates	Cluster-level			Peak-level		
**Excluded SNP**		**(x, y, z)**	k_E	p_{uncorr}	$p_{FWE\text{-}corr}$	**Z**	p_{uncorr}	$p_{FDR\text{-}corr}$
**rs1037791**	Left BA46	-37 49 10	244	0.02	0.09	4.8	<0.001	0.002
	Left BA40	-59 -41 44	185	0.04	0.17	4.1	<0.001	0.005
	Right BA10	34 49 6	32	0.37	0.81	3.34	<0.001	0.018
		34 -7 36	27	0.41	0.84	3.28	0.001	0.02
**rs11213916**	Left BA46	-37 46 10	206	0.02	0.08	4.91	<0.001	0.002
	Left BA40	-56 -41 44	131	0.06	0.2	4.04	<0.001	0.004
**rs1805453**	Left BA10	-37 49 6	88	0.06	0.07	4.32	<0.001	0.01
		-59 -44 44	39	0.2	0.21	3.8	<0.001	0.02
**rs12940715**	Left BA46	-37 49 10	130	0.04	0.08	4.68	<0.001	0.005
	Left BA39	-52 -44 32	118	0.05	0.1	3.99	<0.001	0.009
	Right BA10	34 49 6	31	0.3	0.46	3.50	<0.001	0.018
**rs9297283**	Left BA10	-33 46 10	549	0.007	0.05	4.98	<0.001	0.001
	Left BA40	-56 -41 44	445	0.01	0.1	4.43	<0.001	0.001
	Right BA10	34 49 6	73	0.27	0.9	3.63	<0.001	0.007
		34 -7 36	63	0.3	0.9	3.38	<0.001	0.01
**rs851436**	Left BA10	-37 49 6	235	0.02	0.1	5.16	<0.001	0.001
		-59 -44 48	213	0.03	0.1	4.63	<0.001	0.001
**rs6902039**	Left BA39	-63 -44 36	49	0.3	0.8	4.24	<0.001	0.03
	Left BA10	-37 53 2	190	0.05	0.2	4.03	<0.001	0.03
**rs2486064**	Left BA10	-37 49 6	176	0.03	0.12	4.28	<0.001	0.017
	Left BA40	-56 -41 44	125	0.07	0.2	4.01	<0.001	0.017

We excluded one at a time each SNP from the computation of the PCI (see first column) and computed statistics of the fit of the resulting Polygenic Co-expression Index (PCI) with fMRI activation. Data were thresholded at topological FDR-corrected $q < .05$ to allow unbiased comparison of cluster extents.

7. SI APPENDIX

This appendix reports the outputs of the Haploreg v. 4.1 software (http://www.broadinstitute.org/mammals/haploreg/haploreg.php) regarding the function of the eight SNPs included in the PCI and the SNPs in complete LD with them.
