# Supplementary material for: DRD2 co-expression network and a related polygenic index predict imaging, behavioral and clinical phenotypes linked to schizophrenia
Source: Transl Psychiatry. 2017 Jan 17;7(1):e1006–. doi: 10.1038/tp.2016.253 (PMC5545721; doi:10.1038/tp.2016.253)
Supplement: Supplementary Appendix [file tp2016253x2.pdf]

HaploReg v4.1

HaploReg is a tool for exploring annotations of the noncoding genome at variants on haplotype blocks, such as candidate regulatory SNPs at disease-associated loci. Using LD information from the 1000 Genomes Project, linked SNPs and small indels can be visualized along with chromatin state and protein binding annotation from the Roadmap Epigenomics and ENCODE projects, sequence conservation across mammals, the effect of SNPs on regulatory motifs, and the effect of SNPs on expression from eQTL studies HaploReg is designed for researchers developing mechanistic hypotheses of the impact of non-coding variants on clinical phenotypes and normal variation.

Update 2015.11.05: Version 4.1 GWAS and eQTL have been updated; a simpler pruning strategy is applied when combining GWAS; and links out to other NHGRI/EBI GWAS h and GRASP QTL hits are provided.

Update 2015.09.15: Version 4.0 now includes many recent eQTL results including the GTEx pilot, four different options for defining enhancers using Roadmap Epigenomics dat and a complete set of source files for download and local analysis. Older versions available: v3, v2, v1.

- Build Query
- Set Options
- Documentation

Build Query

Use one of the three methods below to enter a set of variants. If an r² threshold is specified (see the Set Options tab), results for each variant will be shown in a separate table along with other variants in LD. If r² is set to NA, only queried variants will be shown, together in one table.

Query (comma-delimited list of rsIDs OR a single region as chrN:start-end): rs2486064,rs6902039,rs8!

or, upload a text file (one refSNP ID per line): Scegli file Nessun file selezionato

or, select a GWAS:

Set Options

LD threshold, r² (select NA to only show query variants): 1.0

1000G Phase 1 population for LD calculation: AFR AMR ASN EUR

Source for epigenomes: ChromHMM (Core 15-state model)

Mammalian conservation algorithm: GERP SiPhy-omega both

Show position relative to: GENCODE genes RefSeq genes both

Condense lists in table longer than: 3

Condense indel oligos longer than: 6

Output mode: HTML Text

Documentation

For details on data sources and methods along with usage examples, see the full documentation (opens in a pop-up window.)

The HaploReg database and web interface were produced by Luke Ward in the Kellis Lab at MIT. HaploReg is hosted by the Broad Institute.

To cite HaploReg, please refer to our publication in Nucleic Acids Research: HaploReg: a resource for exploring chromatin states, conservation, and regulatory motif alterations within sets of genetically linked variants. (PMID:22064851).

The underlying data are available in the following directory.

Contact: luke@lukeward.net

Invia

Query SNP: rs2486064 and variants with r^2 >= 1

chr	pos (hg38)	LD (r²)	LD (D')	variant	Ref	Alt	AFR freq	AMR freq	ASN freq	EUR freq	SiPhy cons	Promoter histone marks	Enhancer histone marks	DNAse	Proteins bound	Motifs changed	NHGRI/EBI GWAS hits	GRASP QTL hits	Selected eQTL hits	GENCODE genes	dbSNP func annot
1	203199636	1	1	rs2486064	T	C	0.97	0.72	0.66	0.60		FAT, MUS, BONE	14 tissues						3 hits	13kb 5' of CHI3L1	

Query SNP: rs6902039 and variants with r^2 >= 1

chr	pos (hg38)	LD (r²)	LD (D')	variant	Ref	Alt	AFR freq	AMR freq	ASN freq	EUR freq	SiPhy cons	Promoter histone marks	Enhancer histone marks	DNAse	Proteins bound	Motifs changed	NHGRI/EBI GWAS hits	GRASP QTL hits	Selected eQTL hits	GENCODE genes	dbSNP func annot
6	24583953	1	1	rs6902039	T	C	0.10	0.24	0.03	0.35		7 tissues	17 tissues	19 tissues	KAP1,CEBPB	5 altered motifs				KIAA0319	intronic

Query SNP: rs851436 and variants with r^2 >= 1

chr	pos (hg38)	LD (r²)	LD (D')	variant	Ref	Alt	AFR freq	AMR freq	ASN freq	EUR freq	SiPhy cons	Promoter histone marks	Enhancer histone marks	DNAse	Proteins bound	Motifs changed	NHGRI/EBI GWAS hits	GRASP QTL hits	Selected eQTL hits	GENCODE genes	dbSNP func annot
2	19283340	1	1	rs851436	C	A	0.62	0.35	0.40	0.33			ESDR			6 altered motifs		1 hit		AC092594.1	
2	19283774	1	1	rs851434	C	G	0.62	0.35	0.40	0.33						9 altered motifs				AC092594.1	
2	19284033	1	1	rs851433	T	C	0.62	0.35	0.44	0.33			BRST			4 altered motifs				AC092594.1	

Query SNP: rs9297283 and variants with r^2 >= 1

Promoter	Enhancer	GRASP	Selected	dbSNP

chr	pos (hg38)	LD (r²)	LD (D')	variant	Ref	Alt	AFR freq	AMR freq	ASN freq	EUR freq	SiPhy cons	histone marks	histone marks	DNAse	Proteins bound	Motifs changed	NHGRI/EBI GWAS hits	QTL hits	eQTL hits	GENCODE genes	func annot
8	98154668	1	1	rs9297283	G	T	0.28	0.23	0.18	0.18		BRN	10 tissues	IPSC,MUS						POP1	intronic

Query SNP: rs12940715 and variants with r^2 >= 1

chr	pos (hg38)	LD (r²)	LD (D')	variant	Ref	Alt	AFR freq	AMR freq	ASN freq	EUR freq	SiPhy cons	Promoter histone marks	Enhancer histone marks	DNAse	Proteins bound	Motifs changed	NHGRI/EBI GWAS hits	GRASP QTL hits	Selected eQTL hits	GENCODE genes	dbSNP func annot
17	73481592	1	1	rs12940715	C	T	0.01	0.13	0.05	0.20			7 tissues			5 altered motifs				SDK2	intronic

Query SNP: rs1805453 and variants with r^2 >= 1

chr	pos (hg38)	LD (r²)	LD (D')	variant	Ref	Alt	AFR freq	AMR freq	ASN freq	EUR freq	SiPhy cons	Promoter histone marks	Enhancer histone marks	DNAse	Proteins bound	Motifs changed	NHGRI/EBI GWAS hits	GRASP QTL hits	Selected eQTL hits	GENCODE genes	dbSNP func annot
17	5478882	1	1	rs3888575	C	T	0.55	0.13	0.30	0.19				BLD		HNF4,SREBP,Zbtb3				DERL2	intronic
17	5482078	1	1	rs1805453	C	A	0.55	0.13	0.30	0.19			SKIN, PANC			CEBPB,SIX5,TCF12		6 hits	3 hits	DERL2	intronic

Query SNP: rs11213916 and variants with r^2 >= 1

chr	pos (hg38)	LD (r²)	LD (D')	variant	Ref	Alt	AFR freq	AMR freq	ASN freq	EUR freq	SiPhy cons	Promoter histone marks	Enhancer histone marks	DNAse	Proteins bound	Motifs changed	NHGRI/EBI GWAS hits	GRASP QTL hits	Selected eQTL hits	GENCODE genes	dbSNP func annot
11	111468254	1	1	rs11213916	C	T	0.26	0.43	0.26	0.34										BTG4	intronic

Query SNP: rs1037791 and variants with r^2 >= 1

chr	pos (hg38)	LD (r²)	LD (D')	variant	Ref	Alt	AFR freq	AMR freq	ASN freq	EUR freq	SiPhy cons	Promoter histone marks	Enhancer histone marks	DNAse	Proteins bound	Motifs changed	NHGRI/EBI GWAS hits	GRASP QTL hits	Selected eQTL hits	GENCODE genes	dbSNP func annot
7	16785037	1	1	rs1037791	G	A	0.63	0.77	0.72	0.74						5 altered motifs				500bp 3' of TSPAN13	

Query SNP enhancer summary:

NOTE: Background for GWAS SNPs is still based on Haploreg v4.0 and will be updated to reflect v4.1

Cell	Observed	Expected (all SNPs)	Expected (GWAS SNPs)	Binomial p (all SNPs)	Binomial p (GWAS SNPs)
E017 LNG.IMR90 (IMR90 fetal lung fibroblasts Cell Line)	3	0.4	0.7	**0.003999**	**0.022367**
E002 ESC.WA7 (ES-WA7 Cells)	0	0.1	0.2	1	1
E008 ESC.H9 (H9 Cells)	0	0.1	0.2	1	1
E001 ESC.I3 (ES-I3 Cells)	2	0.3	0.5	**0.035358**	0.082347
E015 ESC.HUES6 (HUES6 Cells)	1	0.3	0.5	0.275086	0.388852
E014 ESC.HUES48 (HUES48 Cells)	0	0.3	0.4	1	1
E016 ESC.HUES64 (HUES64 Cells)	1	0.3	0.5	0.250216	0.372271
E003 ESC.H1 (H1 Cells)	0	0.3	0.5	1	1
E024 ESC.4STAR (ES-UCSF4 Cells)	0	0.3	0.6	1	1
E020 IPSC.20B (iPS-20b Cells)	1	0.2	0.3	0.209214	0.29869
E019 IPSC.18 (iPS-18 Cells)	1	0.3	0.4	0.260015	0.360331
E018 IPSC.15b (iPS-15b Cells)	1	0.3	0.4	0.248262	0.368313
E021 IPSC.DF.6.9 (iPS DF 6.9 Cells)	0	0.1	0.3	1	1
E022 IPSC.DF.19.11 (iPS DF 19.11 Cells)	0	0.3	0.6	1	1
E007 ESDR.H1.NEUR.PROG (H1 Derived Neuronal Progenitor Cultured Cells)	1	0.2	0.3	0.146828	0.274409
E009 ESDR.H9.NEUR.PROG (H9 Derived Neuronal Progenitor Cultured Cells)	0	0.2	0.4	1	1
E010 ESDR.H9.NEUR (H9 Derived Neuron Cultured Cells)	0	0.3	0.5	1	1
E013 ESDR.CD56.MESO (hESC Derived CD56+ Mesoderm Cultured Cells)	1	0.3	0.5	0.228141	0.402218
E012 ESDR.CD56.ECTO (hESC Derived CD56+ Ectoderm Cultured Cells)	0	0.2	0.4	1	1
E011 ESDR.CD184.ENDO (hESC Derived CD184+ Endoderm Cultured Cells)	1	0.3	0.5	0.228739	0.389815
E004 ESDR.H1.BMP4.MESO (H1 BMP4 Derived Mesendoderm Cultured Cells)	0	0.1	0.2	1	1
E005 ESDR.H1.BMP4.TROP (H1 BMP4 Derived Trophoblast Cultured Cells)	1	0.3	0.6	0.253534	0.447888
E006 ESDR.H1.MSC (H1 Derived Mesenchymal Stem Cells)	2	0.3	0.6	**0.036303**	0.106024
E062 BLD.PER.MONUC.PC (Primary mononuclear cells from�peripheral�blood)	0	0.1	0.2	1	1
E034 BLD.CD3.PPC (Primary T cells from�peripheral�blood)	0	0.3	0.5	1	1
E045 BLD.CD4.CD25I.CD127.TMEMPC (Primary T cells effector/memory enriched from peripheral blood)	0	0.1	0.2	1	1
E033 BLD.CD3.CPC (Primary T cells from cord blood)	0	0.2	0.3	1	1
E044 BLD.CD4.CD25.CD127M.TREGPC (Primary T regulatory cells from�peripheral�blood)	0	0.2	0.3	1	1
E043 BLD.CD4.CD25M.TPC (Primary T helper cells from�peripheral�blood)	1	0.3	0.4	0.239989	0.36533
E039 BLD.CD4.CD25M.CD45RA.NPC (Primary T helper naive cells from�peripheral�blood)	0	0.2	0.4	1	1
E041 BLD.CD4.CD25M.IL17M.PL.TPC (Primary T helper cells PMA-I stimulated)	0	0.3	0.4	1	1
E042 BLD.CD4.CD25M.IL17P.PL.TPC (Primary T helper 17 cells PMA-I stimulated)	0	0.2	0.4	1	1
E040 BLD.CD4.CD25M.CD45RO.MPC (Primary T helper memory cells from peripheral blood 1)	1	0.2	0.4	0.207716	0.330653
E037 BLD.CD4.MPC (Primary T helper memory cells from peripheral blood 2)	0	0.3	0.4	1	1
E048 BLD.CD8.MPC (Primary T CD8+ memory cells from peripheral blood)	0	0.2	0.4	1	1
E038 BLD.CD4.NPC (Primary T helper naive cells from peripheral blood)	0	0.2	0.4	1	1
E047 BLD.CD8.NPC (Primary T CD8+ naive cells from peripheral blood)	0	0.2	0.4	1	1
E029 BLD.CD14.PC (Primary monocytes from�peripheral�blood)	1	0.3	0.6	0.298844	0.472105

E031 BLD.CD19.CPC (Primary B cells from cord blood)	0	0.2	0.4	1	1
E035 BLD.CD34.PC (Primary hematopoietic stem cells)	1	0.2	0.4	0.210746	0.357316
E051 BLD.MOB.CD34.PC.M (Primary hematopoietic stem cells G-CSF-mobilized Male)	1	0.3	0.5	0.277044	0.431812
E050 BLD.MOB.CD34.PC.F (Primary hematopoietic stem cells G-CSF-mobilized Female)	2	0.3	0.6	**0.040694**	0.112723
E036 BLD.CD34.CC (Primary hematopoietic stem cells short term culture)	0	0.3	0.6	1	1
E032 BLD.CD19.PPC (Primary B cells from peripheral blood)	0	0.3	0.5	1	1
E046 BLD.CD56.PC (Primary Natural Killer cells from�peripheral�blood)	0	0.3	0.5	1	1
E030 BLD.CD15.PC (Primary neutrophils from�peripheral�blood)	1	0.2	0.4	0.213082	0.331696
E026 STRM.MRW.MSC (Bone Marrow Derived Cultured Mesenchymal Stem Cells)	3	0.3	0.5	**0.002133**	**0.010849**
E049 STRM.CHON.MRW.DR.MSC (Mesenchymal Stem Cell Derived Chondrocyte Cultured Cells)	2	0.3	0.6	**0.04112**	0.124991
E025 FAT.ADIP.DR.MSC (Adipose Derived Mesenchymal Stem Cell Cultured Cells)	1	0.5	0.8	0.374504	0.550445
E023 FAT.MSC.DR.ADIP (Mesenchymal Stem Cell Derived Adipocyte Cultured Cells)	1	0.3	0.6	0.294216	0.44877
E052 MUS.SAT (Muscle Satellite Cultured Cells)	3	0.3	0.6	**0.002985**	**0.014321**
E055 SKIN.PEN.FRSK.FIB.01 (Foreskin Fibroblast Primary Cells skin01)	2	0.3	0.6	**0.043344**	0.124493
E056 SKIN.PEN.FRSK.FIB.02 (Foreskin Fibroblast Primary Cells skin02)	1	0.2	0.5	0.213829	0.375225
E059 SKIN.PEN.FRSK.MEL.01 (Foreskin Melanocyte Primary Cells skin01)	0	0.2	0.4	1	1
E061 SKIN.PEN.FRSK.MEL.03 (Foreskin Melanocyte Primary Cells skin03)	1	0.3	0.5	0.296436	0.428184
E057 SKIN.PEN.FRSK.KER.02 (Foreskin Keratinocyte Primary Cells skin02)	0	0.3	0.6	1	1
E058 SKIN.PEN.FRSK.KER.03 (Foreskin Keratinocyte Primary Cells skin03)	2	0.3	0.6	**0.039705**	0.102724
E028 BRST.HMEC.35 (Breast variant Human Mammary Epithelial Cells (vHMEC))	2	0.3	0.6	**0.043393**	0.120038
E027 BRST.MYO (Breast Myoepithelial Primary Cells)	2	0.4	0.8	0.058615	0.175169
E054 BRN.GANGEM.DR.NRSPHR (Ganglion Eminence derived primary cultured neurospheres)	1	0.2	0.3	0.196433	0.293235
E053 BRN.CRTX.DR.NRSPHR (Cortex derived primary cultured neurospheres)	1	0.3	0.5	0.268608	0.385954
E112 THYM (Thymus)	0	0.1	0.3	1	1
E093 THYM.FET (Fetal Thymus)	0	0.3	0.6	1	1
E071 BRN.HIPP.MID (Brain Hippocampus Middle)	3	0.3	0.5	**0.002021**	**0.01007**
E074 BRN.SUB.NIG (Brain Substantia Nigra)	3	0.2	0.5	**0.001476**	**0.008312**
E068 BRN.ANT.CAUD (Brain Anterior Caudate)	1	0.2	0.4	0.208005	0.346151
E069 BRN.CING.GYR (Brain Cingulate Gyrus)	3	0.2	0.4	**0.001217**	**0.006176**
E072 BRN.INF.TMP (Brain Inferior Temporal Lobe)	3	0.2	0.4	**0.001038**	**0.004728**
E067 BRN.ANG.GYR (Brain Angular Gyrus)	3	0.2	0.3	**0.000704**	**0.003558**
E073 BRN.DL.PRFRNTL.CRTX (Brain Dorsolateral Prefrontal Cortex)	3	0.2	0.3	**0.000491**	**0.00347**
E070 BRN.GRM.MTRX (Brain Germinal Matrix)	1	0.2	0.3	0.172888	0.266531
E082 BRN.FET.F (Fetal Brain Female)	2	0.1	0.2	**0.007933**	**0.019857**
E081 BRN.FET.M (Fetal Brain Male)	1	0.3	0.4	0.245341	0.330653
E063 FAT.ADIP.NUC (Adipose Nuclei)	2	0.3	0.6	**0.036529**	0.123499
E100 MUS.PSOAS (Psoas Muscle)	1	0.2	0.3	0.157684	0.259719
E108 MUS.SKLT.F (Skeletal Muscle Female)	1	0.3	0.6	0.274796	0.452283
E107 MUS.SKLT.M (Skeletal Muscle Male)	0	0.3	0.6	1	1
E089 MUS.TRNK.FET (Fetal Muscle Trunk)	1	0.3	0.6	0.295804	0.475488
E090 MUS.LEG.FET (Fetal Muscle Leg)	2	0.4	0.8	0.069929	0.203368
E083 HRT.FET (Fetal Heart)	2	0.4	0.7	0.060215	0.146919
E104 HRT.ATR.R (Right Atrium)	1	0.2	0.4	0.185358	0.327515
E095 HRT.VENT.L (Left Ventricle)	1	0.3	0.5	0.243184	0.405989
E105 HRT.VNT.R (Right Ventricle)	2	0.2	0.4	**0.018403**	0.060445
E065 VAS.AOR (Aorta)	0	0.1	0.2	1	1
E078 GI.DUO.SM.MUS (Duodenum Smooth Muscle)	0	0.2	0.3	1	1
E076 GI.CLN.SM.MUS (Colon Smooth Muscle)	2	0.2	0.5	**0.023991**	0.070883
E103 GI.RECT.SM.MUS (Rectal Smooth Muscle)	0	0.2	0.3	1	1
E111 GI.STMC.MUS (Stomach Smooth Muscle)	1	0.2	0.3	0.168758	0.279991
E092 GI.STMC.FET (Fetal Stomach)	1	0.3	0.6	0.253995	0.437215
E085 GI.S.INT.FET (Fetal Intestine Small)	1	0.3	0.5	0.26658	0.426363
E084 GI.L.INT.FET (Fetal Intestine Large)	0	0.3	0.5	1	1
E109 GI.S.INT (Small Intestine)	0	0.1	0.2	1	1
E106 GI.CLN.SIG (Sigmoid Colon)	0	0.2	0.3	1	1
E075 GI.CLN.MUC (Colonic Mucosa)	1	0.1	0.2	0.098256	0.188489
E101 GI.RECT.MUC.29 (Rectal Mucosa Donor 29)	1	0.1	0.3	0.123452	0.231912
E102 GI.RECT.MUC.31 (Rectal Mucosa Donor 31)	1	0.2	0.4	0.189173	0.332738
E110 GI.STMC.MUC (Stomach Mucosa)	1	0.3	0.5	0.249078	0.394612
E077 GI.DUO.MUC (Duodenum Mucosa)	0	0.2	0.4	1	1
E079 GI.ESO (Esophagus)	1	0.2	0.3	0.155213	0.285536
E094 GI.STMC.GAST (Gastric)	0	0.2	0.3	1	1
E099 PLCNT.AMN (Placenta Amnion)	0	0.2	0.4	1	1
E086 KID.FET (Fetal Kidney)	1	0.1	0.3	0.127869	0.236609
E088 LNG.FET (Fetal Lung)	2	0.3	0.6	**0.038113**	0.112723
E097 OVRY (Ovary)	0	0.2	0.4	1	1
E087 PANC.ISLT (Pancreatic Islets)	0	0.1	0.2	1	1
E080 ADRL.GLND.FET (Fetal Adrenal Gland)	2	0.4	0.7	0.054219	0.159029
E091 PLCNT.FET (Placenta)	1	0.4	0.7	0.312715	0.530926
E066 LIV.ADLT (Liver)	1	0.3	0.6	0.270443	0.435419
E098 PANC (Pancreas)	3	0.2	0.4	**0.001513**	**0.006494**
E096 LNG (Lung)	1	0.2	0.4	0.180606	0.326466
E113 SPLN (Spleen)	1	0.3	0.5	0.225455	0.406929
E114 LNG.A549.ETOH002.CNCR (A549 EtOH 0.02pct Lung Carcinoma Cell Line)	1	0.2	0.4	0.220325	0.368313

E115 BLD.DND41.CNCR (Dnd41 TCell Leukemia Cell Line)	0	0.2	0.3	1	1
E116 BLD.GM12878 (GM12878 Lymphoblastoid Cells)	0	0.2	0.4	1	1
E117 CRVX.HELAS3.CNCR (HeLa-S3 Cervical Carcinoma Cell Line)	0	0.2	0.4	1	1
E118 LIV.HEPG2.CNCR (HepG2 Hepatocellular Carcinoma Cell Line)	1	0.4	0.7	0.334884	0.497016
E119 BRST.HMEC (HMEC Mammary Epithelial Primary Cells)	1	0.3	0.6	0.288989	0.452283
E120 MUS.HSMM (HSMM Skeletal Muscle Myoblasts Cells)	3	0.3	0.5	**0.001608**	**0.011571**
E121 MUS.HSMMT (HSMM cell derived Skeletal Muscle Myotubes Cells)	1	0.3	0.5	0.227948	0.378168
E122 VAS.HUVEC (HUVEC Umbilical Vein Endothelial Primary Cells)	0	0.3	0.4	1	1
E123 BLD.K562.CNCR (K562 Leukemia Cells)	0	0.3	0.4	1	1
E124 BLD.CD14.MONO (Monocytes-CD14+ RO01746 Primary Cells)	2	0.2	0.4	**0.021233**	0.055462
E125 BRN.NHA (NH-A Astrocytes Primary Cells)	2	0.2	0.5	**0.022671**	0.071711
E126 SKIN.NHDFAD (NHDF-Ad Adult Dermal Fibroblast Primary Cells)	2	0.3	0.6	**0.041868**	0.110316
E127 SKIN.NHEK (NHEK-Epidermal Keratinocyte Primary Cells)	1	0.3	0.5	0.271009	0.412539
E128 LNG.NHLF (NHLF Lung Fibroblast Primary Cells)	1	0.2	0.4	0.204556	0.358322
E129 BONE.OSTEO (Osteoblast Primary Cells)	2	0.3	0.5	**0.034159**	0.090766

Detail view for rs2486064

Link to dbSNP entry

Link to Ensembl Variation entry

Sequence facts

chr	pos (hg19)	chr	pos (hg38)	Reference	Alternate	1000 Genomes Phase 1 Frequencies				Sequence constraint		dbSNP functional annotation
						AFR	AMR	ASN	EUR	by GERP	by SiPhy	
chr1	203168764	chr1	203199636	T	C	0.97	0.72	0.66	0.6	No	No	none

Closest annotated gene					
Source	Distance	Direction	ID/Link	Common name	Description
GENCODE	5'	12886	ENSG00000133048.8	CHI3L1	chitinase 3-like 1 (cartilage glycoprotein-39) [Source:HGNC Symbol;Acc:1932]
RefSeq	5'	12841	NM_001276	CHI3L1	chitinase 3-like 1 (cartilage glycoprotein-39) [Source:HGNC Symbol;Acc:1932]

Regulatory chromatin states from DNAse and histone ChIP-Seq (Roadmap Epigenomics Consortium, 2015)

(Black = missing data)

Epigenome ID (EID)	Group	Mnemonic	Description	Chromatin states (Core 15-state model)	Chromatin states (25-state model using 12 imputed marks)	H3K4me1	H3K4me3	H3K27ac	H3K9ac	DNas
E017	IMR90	LNG.IMR90	IMR90 fetal lung fibroblasts Cell Line	7_Enh	13_EnhA1	H3K4me1_Enh		H3K27ac_Enh	H3K9ac_Pro	
E002	ESC	ESC.WA7	ES-WA7 Cells							
E008	ESC	ESC.H9	H9 Cells							
E001	ESC	ESC.I3	ES-I3 Cells							
E015	ESC	ESC.HUES6	HUES6 Cells							
E014	ESC	ESC.HUES48	HUES48 Cells							
E016	ESC	ESC.HUES64	HUES64 Cells							
E003	ESC	ESC.H1	H1 Cells							
E024	ESC	ESC.4STAR	ES-UCSF4 Cells			H3K4me1_Enh				
E020	iPSC	IPSC.20B	iPS-20b Cells							
E019	iPSC	IPSC.18	iPS-18 Cells							
E018	iPSC	IPSC.15b	iPS-15b Cells							
E021	iPSC	IPSC.DF.6.9	iPS DF 6.9 Cells							
E022	iPSC	IPSC.DF.19.11	iPS DF 19.11 Cells		17_EnhW2	H3K4me1_Enh				
E007	ES-deriv	ESDR.H1.NEUR.PROG	H1 Derived Neuronal Progenitor Cultured Cells							
E009	ES-deriv	ESDR.H9.NEUR.PROG	H9 Derived Neuronal Progenitor Cultured Cells							
E010	ES-deriv	ESDR.H9.NEUR	H9 Derived Neuron Cultured Cells							
E013	ES-deriv	ESDR.CD56.MESO	hESC Derived CD56+ Mesoderm Cultured Cells	7_Enh	15_EnhAF	H3K4me1_Enh				
E012	ES-deriv	ESDR.CD56.ECTO	hESC Derived CD56+ Ectoderm Cultured Cells							
E011	ES-deriv	ESDR.CD184.ENDO	hESC Derived CD184+ Endoderm Cultured Cells							
E004	ES-deriv	ESDR.H1.BMP4.MESO	H1 BMP4 Derived Mesendoderm Cultured Cells							
E005	ES-deriv	ESDR.H1.BMP4.TROP	H1 BMP4 Derived Trophoblast Cultured Cells		15_EnhAF	H3K4me1_Enh				
E006	ES-deriv	ESDR.H1.MSC	H1 Derived Mesenchymal Stem Cells		15_EnhAF	H3K4me1_Enh				
E062	Blood & T-cell	BLD.PER.MONUC.PC	Primary mononuclear cells from�peripheral�blood		17_EnhW2	H3K4me1_Enh				
E034	Blood & T-cell	BLD.CD3.PPC	Primary T cells from�peripheral�blood							
E045	Blood & T-cell	BLD.CD4.CD25I.CD127.TMEMPC	Primary T cells effector/memory enriched from peripheral blood							
E033	Blood & T-cell	BLD.CD3.CPC	Primary T cells from cord blood							
E044	Blood & T-cell	BLD.CD4.CD25.CD127M.TREGPC	Primary T regulatory cells from�peripheral�blood							
E043	Blood & T-cell	BLD.CD4.CD25M.TPC	Primary T helper cells from�peripheral�blood							
E039	Blood & T-cell	BLD.CD4.CD25M.CD45RA.NPC	Primary T helper naive cells from�peripheral�blood							
E041	Blood & T-cell	BLD.CD4.CD25M.IL17M.PL.TPC	Primary T helper cells PMA-I stimulated							
E042	Blood & T-cell	BLD.CD4.CD25M.IL17P.PL.TPC	Primary T helper 17 cells PMA-I stimulated							
E040	Blood & T-cell	BLD.CD4.CD25M.CD45RO.MPC	Primary T helper memory cells from peripheral blood 1							
E037	Blood & T-cell	BLD.CD4.MPC	Primary T helper memory cells from peripheral blood 2							

E048	Blood & T-cell	BLD.CD8.MPC	Primary T CD8+ memory cells from peripheral blood							
E038	Blood & T-cell	BLD.CD4.NPC	Primary T helper naive cells from peripheral blood							
E047	Blood & T-cell	BLD.CD8.NPC	Primary T CD8+ naive cells from peripheral blood							
E029	HSC & B-cell	BLD.CD14.PC	Primary monocytes from�peripheral�blood	7_Enh	15_EnhAF	H3K4me1_Enh				
E031	HSC & B-cell	BLD.CD19.CPC	Primary B cells from cord blood		17_EnhW2					
E035	HSC & B-cell	BLD.CD34.PC	Primary hematopoietic stem cells		17_EnhW2					
E051	HSC & B-cell	BLD.MOB.CD34.PC.M	Primary hematopoietic stem cells G-CSF-mobilized Male							
E050	HSC & B-cell	BLD.MOB.CD34.PC.F	Primary hematopoietic stem cells G-CSF-mobilized Female							
E036	HSC & B-cell	BLD.CD34.CC	Primary hematopoietic stem cells short term culture		17_EnhW2	H3K4me1_Enh				
E032	HSC & B-cell	BLD.CD19.PPC	Primary B cells from peripheral blood							
E046	HSC & B-cell	BLD.CD56.PC	Primary Natural Killer cells from�peripheral�blood							
E030	HSC & B-cell	BLD.CD15.PC	Primary neutrophils from�peripheral�blood	7_Enh	13_EnhA1	H3K4me1_Enh				
E026	Mesench	STRM.MRW.MSC	Bone Marrow Derived Cultured Mesenchymal Stem Cells	7_Enh	13_EnhA1	H3K4me1_Enh	H3K4me3_Pro	H3K27ac_Enh	H3K9ac_Pro	
E049	Mesench	STRM.CHON.MRW.DR.MSC	Mesenchymal Stem Cell Derived Chondrocyte Cultured Cells	7_Enh	13_EnhA1	H3K4me1_Enh	H3K4me3_Pro	H3K27ac_Enh	H3K9ac_Pro	
E025	Mesench	FAT.ADIP.DR.MSC	Adipose Derived Mesenchymal Stem Cell Cultured Cells	2_TssAFlnk	13_EnhA1	H3K4me1_Enh	H3K4me3_Pro		H3K9ac_Pro	
E023	Mesench	FAT.MSC.DR.ADIP	Mesenchymal Stem Cell Derived Adipocyte Cultured Cells	7_Enh	13_EnhA1	H3K4me1_Enh	H3K4me3_Pro		H3K9ac_Pro	
E052	Myosat	MUS.SAT	Muscle Satellite Cultured Cells	7_Enh	14_EnhA2	H3K4me1_Enh				
E055	Epithelial	SKIN.PEN.FRSK.FIB.01	Foreskin Fibroblast Primary Cells skin01	12_EnhBiv	15_EnhAF	H3K4me1_Enh				
E056	Epithelial	SKIN.PEN.FRSK.FIB.02	Foreskin Fibroblast Primary Cells skin02		15_EnhAF	H3K4me1_Enh		H3K27ac_Enh		
E059	Epithelial	SKIN.PEN.FRSK.MEL.01	Foreskin Melanocyte Primary Cells skin01							
E061	Epithelial	SKIN.PEN.FRSK.MEL.03	Foreskin Melanocyte Primary Cells skin03							
E057	Epithelial	SKIN.PEN.FRSK.KER.02	Foreskin Keratinocyte Primary Cells skin02		17_EnhW2	H3K4me1_Enh				
E058	Epithelial	SKIN.PEN.FRSK.KER.03	Foreskin Keratinocyte Primary Cells skin03		17_EnhW2					
E028	Epithelial	BRST.HMEC.35	Breast variant Human Mammary Epithelial Cells (vHMEC)	7_Enh	15_EnhAF	H3K4me1_Enh				
E027	Epithelial	BRST.MYO	Breast Myoepithelial Primary Cells	7_Enh	15_EnhAF	H3K4me1_Enh				
E054	Neurosph	BRN.GANGEM.DR.NRSPHR	Ganglion Eminence derived primary cultured neurospheres		18_EnhAc	H3K4me1_Enh				
E053	Neurosph	BRN.CRTX.DR.NRSPHR	Cortex derived primary cultured neurospheres			H3K4me1_Enh				
E112	Thymus	THYM	Thymus		17_EnhW2					
E093	Thymus	THYM.FET	Fetal Thymus							
E071	Brain	BRN.HIPP.MID	Brain Hippocampus Middle	7_Enh	14_EnhA2	H3K4me1_Enh	H3K4me3_Pro	H3K27ac_Enh		
E074	Brain	BRN.SUB.NIG	Brain Substantia Nigra	7_Enh	15_EnhAF	H3K4me1_Enh		H3K27ac_Enh		
E068	Brain	BRN.ANT.CAUD	Brain Anterior Caudate	7_Enh	14_EnhA2	H3K4me1_Enh	H3K4me3_Pro	H3K27ac_Enh	H3K9ac_Pro	
E069	Brain	BRN.CING.GYR	Brain Cingulate Gyrus	7_Enh	14_EnhA2	H3K4me1_Enh		H3K27ac_Enh	H3K9ac_Pro	
E072	Brain	BRN.INF.TMP	Brain Inferior Temporal Lobe	7_Enh	15_EnhAF	H3K4me1_Enh		H3K27ac_Enh		
E067	Brain	BRN.ANG.GYR	Brain Angular Gyrus	7_Enh	15_EnhAF	H3K4me1_Enh		H3K27ac_Enh		
E073	Brain	BRN.DL.PRFRNTL.CRTX	Brain_Dorsolateral_Prefrontal_Cortex	7_Enh	14_EnhA2	H3K4me1_Enh		H3K27ac_Enh	H3K9ac_Pro	
E070	Brain	BRN.GRM.MTRX	Brain Germinal Matrix		15_EnhAF					
E082	Brain	BRN.FET.F	Fetal Brain Female							
E081	Brain	BRN.FET.M	Fetal Brain Male		17_EnhW2					
E063	Adipose	FAT.ADIP.NUC	Adipose Nuclei	7_Enh	14_EnhA2	H3K4me1_Enh	H3K4me3_Pro	H3K27ac_Enh		
E100	Muscle	MUS.PSOAS	Psoas Muscle	7_Enh	15_EnhAF	H3K4me1_Enh		H3K27ac_Enh		
E108	Muscle	MUS.SKLT.F	Skeletal Muscle Female	2_TssAFlnk	13_EnhA1	H3K4me1_Enh	H3K4me3_Pro	H3K27ac_Enh	H3K9ac_Pro	
E107	Muscle	MUS.SKLT.M	Skeletal Muscle Male	2_TssAFlnk	13_EnhA1	H3K4me1_Enh	H3K4me3_Pro		H3K9ac_Pro	
E089	Muscle	MUS.TRNK.FET	Fetal Muscle Trunk	12_EnhBiv	17_EnhW2	H3K4me1_Enh				
E090	Muscle	MUS.LEG.FET	Fetal Muscle Leg	7_Enh	17_EnhW2	H3K4me1_Enh				
E083	Heart	HRT.FET	Fetal Heart		15_EnhAF	H3K4me1_Enh				
E104	Heart	HRT.ATR.R	Right Atrium	7_Enh	15_EnhAF	H3K4me1_Enh		H3K27ac_Enh		
E095	Heart	HRT.VENT.L	Left Ventricle		15_EnhAF	H3K4me1_Enh		H3K27ac_Enh		
E105	Heart	HRT.VNT.R	Right Ventricle	7_Enh	15_EnhAF	H3K4me1_Enh		H3K27ac_Enh		
E065	Heart	VAS.AOR	Aorta		15_EnhAF	H3K4me1_Enh				
E078	Sm. Muscle	GI.DUO.SM.MUS	Duodenum Smooth Muscle		14_EnhA2	H3K4me1_Enh		H3K27ac_Enh		
E076	Sm. Muscle	GI.CLN.SM.MUS	Colon Smooth Muscle	7_Enh	14_EnhA2	H3K4me1_Enh	H3K4me3_Pro			
E103	Sm. Muscle	GI.RECT.SM.MUS	Rectal Smooth Muscle		17_EnhW2	H3K4me1_Enh				
E111	Sm. Muscle	GI.STMC.MUS	Stomach Smooth Muscle	7_Enh	15_EnhAF	H3K4me1_Enh		H3K27ac_Enh		
E092	Digestive	GI.STMC.FET	Fetal Stomach	7_Enh	17_EnhW2	H3K4me1_Enh				
E085	Digestive	GI.S.INT.FET	Fetal Intestine Small							
E084	Digestive	GI.L.INT.FET	Fetal Intestine Large							
E109	Digestive	GI.S.INT	Small Intestine		15_EnhAF	H3K4me1_Enh		H3K27ac_Enh		
E106	Digestive	GI.CLN.SIG	Sigmoid Colon		17_EnhW2			H3K27ac_Enh		

E075	Digestive	GI.CLN.MUC	Colonic Mucosa	12_EnhBiv	15_EnhAF	H3K4me1_Enh		H3K27ac_Enh		
E101	Digestive	GI.RECT.MUC.29	Rectal Mucosa Donor 29		17_EnhW2	H3K4me1_Enh		H3K27ac_Enh		
E102	Digestive	GI.RECT.MUC.31	Rectal Mucosa Donor 31		17_EnhW2					
E110	Digestive	GI.STMC.MUC	Stomach Mucosa		17_EnhW2	H3K4me1_Enh			H3K9ac_Pro	
E077	Digestive	GI.DUO.MUC	Duodenum Mucosa		17_EnhW2					
E079	Digestive	GI.ESO	Esophagus	7_Enh	15_EnhAF	H3K4me1_Enh		H3K27ac_Enh		
E094	Digestive	GI.STMC.GAST	Gastric		14_EnhA2	H3K4me1_Enh	H3K4me3_Pro	H3K27ac_Enh		
E099	Other	PLCNT.AMN	Placenta Amnion		17_EnhW2	H3K4me1_Enh		H3K27ac_Enh		
E086	Other	KID.FET	Fetal Kidney		15_EnhAF					
E088	Other	LNG.FET	Fetal Lung	7_Enh	14_EnhA2	H3K4me1_Enh	H3K4me3_Pro			
E097	Other	OVRY	Ovary		15_EnhAF					
E087	Other	PANC.ISLT	Pancreatic Islets		15_EnhAF					
E080	Other	ADRL.GLND.FET	Fetal Adrenal Gland							
E091	Other	PLCNT.FET	Placenta	7_Enh	17_EnhW2	H3K4me1_Enh		H3K27ac_Enh		
E066	Other	LIV.ADLT	Liver		15_EnhAF	H3K4me1_Enh				
E098	Other	PANC	Pancreas	7_Enh	15_EnhAF	H3K4me1_Enh				
E096	Other	LNG	Lung	7_Enh	15_EnhAF	H3K4me1_Enh		H3K27ac_Enh		
E113	Other	SPLN	Spleen	7_Enh	15_EnhAF	H3K4me1_Enh		H3K27ac_Enh		
E114	ENCODE2012	LNG.A549.ETOH002.CNCR	A549 EtOH 0.02pct Lung Carcinoma Cell Line		15_EnhAF					
E115	ENCODE2012	BLD.DND41.CNCR	Dnd41 TCell Leukemia Cell Line							
E116	ENCODE2012	BLD.GM12878	GM12878 Lymphoblastoid Cells							
E117	ENCODE2012	CRVX.HELAS3.CNCR	HeLa-S3 Cervical Carcinoma Cell Line		14_EnhA2	H3K4me1_Enh				
E118	ENCODE2012	LIV.HEPG2.CNCR	HepG2 Hepatocellular Carcinoma Cell Line		15_EnhAF	H3K4me1_Enh				
E119	ENCODE2012	BRST.HMEC	HMEC Mammary Epithelial Primary Cells	7_Enh	15_EnhAF	H3K4me1_Enh				
E120	ENCODE2012	MUS.HSMM	HSMM Skeletal Muscle Myoblasts Cells	7_Enh	15_EnhAF	H3K4me1_Enh		H3K27ac_Enh		
E121	ENCODE2012	MUS.HSMMT	HSMM cell derived Skeletal Muscle Myotubes Cells	7_Enh	13_EnhA1	H3K4me1_Enh	H3K4me3_Pro	H3K27ac_Enh	H3K9ac_Pro	
E122	ENCODE2012	VAS.HUVEC	HUVEC Umbilical Vein Endothelial Primary Cells		15_EnhAF					
E123	ENCODE2012	BLD.K562.CNCR	K562 Leukemia Cells							
E124	ENCODE2012	BLD.CD14.MONO	Monocytes-CD14+ RO01746 Primary Cells	7_Enh	14_EnhA2	H3K4me1_Enh	H3K4me3_Pro	H3K27ac_Enh	H3K9ac_Pro	
E125	ENCODE2012	BRN.NHA	NH-A Astrocytes Primary Cells		15_EnhAF	H3K4me1_Enh				
E126	ENCODE2012	SKIN.NHDFAD	NHDF-Ad Adult Dermal Fibroblast Primary Cells	7_Enh	14_EnhA2	H3K4me1_Enh		H3K27ac_Enh		
E127	ENCODE2012	SKIN.NHEK	NHEK-Epidermal Keratinocyte Primary Cells	7_Enh	15_EnhAF	H3K4me1_Enh				
E128	ENCODE2012	LNG.NHLF	NHLF Lung Fibroblast Primary Cells	7_Enh	14_EnhA2	H3K4me1_Enh		H3K27ac_Enh		
E129	ENCODE2012	BONE.OSTEO	Osteoblast Primary Cells	2_TssAFlnk	13_EnhA1	H3K4me1_Enh	H3K4me3_Pro	H3K27ac_Enh		

Hits from selected eQTL studies

Study ID	Paper Title	PMID	Tissue	Correlated gene	p-value
GTEx2015_v6	The Genotype-Tissue Expression (GTEx) pilot analysis: Multitissue gene regulation in humans	25954001	Whole_Blood	CHI3L1	8.35628893846662e-08
GTEx2015_v6	The Genotype-Tissue Expression (GTEx) pilot analysis: Multitissue gene regulation in humans	25954001	Whole_Blood	MYBPH	9.44541899463964e-06
Westra2013	Systematic identification of trans eQTLs as putative drivers of known disease associations	24013639	Whole_Blood	MYBPH	2.2675801166291686E-112

Detail view for rs6902039

Link to dbSNP entry

Link to Ensembl Variation entry

Sequence facts

chr	pos (hg19)	chr	pos (hg38)	Reference	Alternate	1000 Genomes Phase 1 Frequencies				Sequence constraint		dbSNP functional annotation
						AFR	AMR	ASN	EUR	by GERP	by SiPhy	
chr6	24584181	chr6	24583953	T	C	0.1	0.24	0.03	0.35	No	No	intronic

Closest annotated gene					
Source	Distance	Direction	ID/Link	Common name	Description
GENCODE	NA	Within gene	ENSG00000137261.9	KIAA0319	KIAA0319 [Source:HGNC Symbol;Acc:21580]
RefSeq	NA	Within gene	NM_001168375	KIAA0319	KIAA0319 [Source:HGNC Symbol;Acc:21580]

Regulatory chromatin states from DNAse and histone ChIP-Seq (Roadmap Epigenomics Consortium, 2015)

(Black = missing data)

Epigenome ID (EID)	Group	Mnemonic	Description	Chromatin states (Core 15-state model)	Chromatin states (25-state model using 12 imputed marks)	H3K4me1	H3K4me3	H3K27ac	H3K9ac	DNas
E017	IMR90	LNG.IMR90	IMR90 fetal lung fibroblasts Cell Line	7_Enh	19_DNase	H3K4me1_Enh				
E002	ESC	ESC.WA7	ES-WA7 Cells		17_EnhW2					
E008	ESC	ESC.H9	H9 Cells		17_EnhW2	H3K4me1_Enh				
E001	ESC	ESC.I3	ES-I3 Cells	7_Enh	16_EnhW1	H3K4me1_Enh	H3K4me3_Pro		H3K9ac_Pro	
E015	ESC	ESC.HUES6	HUES6 Cells	7_Enh	17_EnhW2	H3K4me1_Enh				
E014	ESC	ESC.HUES48	HUES48 Cells		17_EnhW2	H3K4me1_Enh				
E016	ESC	ESC.HUES64	HUES64 Cells	7_Enh	19_DNase	H3K4me1_Enh				
E003	ESC	ESC.H1	H1 Cells		17_EnhW2	H3K4me1_Enh			H3K9ac_Pro	DNas
E024	ESC	ESC.4STAR	ES-UCSF4 Cells		19_DNase	H3K4me1_Enh				
E020	iPSC	IPSC.20B	iPS-20b Cells	7_Enh	17_EnhW2	H3K4me1_Enh				
E019	iPSC	IPSC.18	iPS-18 Cells	7_Enh	16_EnhW1	H3K4me1_Enh			H3K9ac_Pro	
E018	iPSC	IPSC.15b	iPS-15b Cells	7_Enh	16_EnhW1	H3K4me1_Enh				
E021	iPSC	IPSC.DF.6.9	iPS DF 6.9 Cells							
E022	iPSC	IPSC.DF.19.11	iPS DF 19.11 Cells							
E007	ES-deriv	ESDR.H1.NEUR.PROG	H1 Derived Neuronal Progenitor Cultured Cells		19_DNase					
E009	ES-deriv	ESDR.H9.NEUR.PROG	H9 Derived Neuronal Progenitor Cultured Cells		17_EnhW2					
E010	ES-deriv	ESDR.H9.NEUR	H9 Derived Neuron Cultured Cells		16_EnhW1	H3K4me1_Enh				
E013	ES-deriv	ESDR.CD56.MESO	hESC Derived CD56+ Mesoderm Cultured Cells		19_DNase					
E012	ES-deriv	ESDR.CD56.ECTO	hESC Derived CD56+ Ectoderm Cultured Cells							
E011	ES-deriv	ESDR.CD184.ENDO	hESC Derived CD184+ Endoderm Cultured Cells	7_Enh	16_EnhW1	H3K4me1_Enh	H3K4me3_Pro			
E004	ES-deriv	ESDR.H1.BMP4.MESO	H1 BMP4 Derived Mesendoderm Cultured Cells		16_EnhW1	H3K4me1_Enh		H3K27ac_Enh	H3K9ac_Pro	
E005	ES-deriv	ESDR.H1.BMP4.TROP	H1 BMP4 Derived Trophoblast Cultured Cells		19_DNase					
E006	ES-deriv	ESDR.H1.MSC	H1 Derived Mesenchymal Stem Cells		17_EnhW2					DNas
E062	Blood & T-cell	BLD.PER.MONUC.PC	Primary mononuclear cells from�peripheral�blood		19_DNase				H3K9ac_Pro	
E034	Blood & T-cell	BLD.CD3.PPC	Primary T cells from�peripheral�blood							
E045	Blood & T-cell	BLD.CD4.CD25I.CD127.TMEMPC	Primary T cells effector/memory enriched from peripheral blood			H3K4me1_Enh				
E033	Blood & T-cell	BLD.CD3.CPC	Primary T cells from cord blood		17_EnhW2					
E044	Blood & T-cell	BLD.CD4.CD25.CD127M.TREGPC	Primary T regulatory cells from�peripheral�blood		17_EnhW2					
E043	Blood & T-cell	BLD.CD4.CD25M.TPC	Primary T helper cells from�peripheral�blood	7_Enh						
E039	Blood & T-cell	BLD.CD4.CD25M.CD45RA.NPC	Primary T helper naive cells from�peripheral�blood							
E041	Blood & T-cell	BLD.CD4.CD25M.IL17M.PL.TPC	Primary T helper cells PMA-I stimulated			H3K4me1_Enh				
E042	Blood & T-cell	BLD.CD4.CD25M.IL17P.PL.TPC	Primary T helper 17 cells PMA-I stimulated		16_EnhW1					
E040	Blood & T-cell	BLD.CD4.CD25M.CD45RO.MPC	Primary T helper memory cells from peripheral blood 1	7_Enh	22_PromP	H3K4me1_Enh				
E037	Blood & T-cell	BLD.CD4.MPC	Primary T helper memory cells from peripheral blood 2							

E048	Blood & T-cell	BLD.CD8.MPC	Primary T CD8+ memory cells from peripheral blood							
E038	Blood & T-cell	BLD.CD4.NPC	Primary T helper naive cells from peripheral blood			H3K4me1_Enh			H3K9ac_Pro	
E047	Blood & T-cell	BLD.CD8.NPC	Primary T CD8+ naive cells from peripheral blood							
E029	HSC & B-cell	BLD.CD14.PC	Primary monocytes from�peripheral�blood							
E031	HSC & B-cell	BLD.CD19.CPC	Primary B cells from cord blood		17_EnhW2					
E035	HSC & B-cell	BLD.CD34.PC	Primary hematopoietic stem cells		17_EnhW2	H3K4me1_Enh				
E051	HSC & B-cell	BLD.MOB.CD34.PC.M	Primary hematopoietic stem cells G-CSF-mobilized Male	2_TssAFlnk	16_EnhW1	H3K4me1_Enh	H3K4me3_Pro			
E050	HSC & B-cell	BLD.MOB.CD34.PC.F	Primary hematopoietic stem cells G-CSF-mobilized Female	7_Enh	16_EnhW1	H3K4me1_Enh				
E036	HSC & B-cell	BLD.CD34.CC	Primary hematopoietic stem cells short term culture		17_EnhW2					
E032	HSC & B-cell	BLD.CD19.PPC	Primary B cells from peripheral blood			H3K4me1_Enh				
E046	HSC & B-cell	BLD.CD56.PC	Primary Natural Killer cells from�peripheral�blood							
E030	HSC & B-cell	BLD.CD15.PC	Primary neutrophils from�peripheral�blood		17_EnhW2					
E026	Mesench	STRM.MRW.MSC	Bone Marrow Derived Cultured Mesenchymal Stem Cells	7_Enh	16_EnhW1	H3K4me1_Enh				
E049	Mesench	STRM.CHON.MRW.DR.MSC	Mesenchymal Stem Cell Derived Chondrocyte Cultured Cells	2_TssAFlnk	16_EnhW1	H3K4me1_Enh	H3K4me3_Pro		H3K9ac_Pro	
E025	Mesench	FAT.ADIP.DR.MSC	Adipose Derived Mesenchymal Stem Cell Cultured Cells	2_TssAFlnk	16_EnhW1	H3K4me1_Enh	H3K4me3_Pro		H3K9ac_Pro	
E023	Mesench	FAT.MSC.DR.ADIP	Mesenchymal Stem Cell Derived Adipocyte Cultured Cells	2_TssAFlnk	2_PromU	H3K4me1_Enh	H3K4me3_Pro		H3K9ac_Pro	
E052	Myosat	MUS.SAT	Muscle Satellite Cultured Cells	7_Enh	16_EnhW1	H3K4me1_Enh				
E055	Epithelial	SKIN.PEN.FRSK.FIB.01	Foreskin Fibroblast Primary Cells skin01		16_EnhW1	H3K4me1_Enh				DNas
E056	Epithelial	SKIN.PEN.FRSK.FIB.02	Foreskin Fibroblast Primary Cells skin02		19_DNase	H3K4me1_Enh				DNas
E059	Epithelial	SKIN.PEN.FRSK.MEL.01	Foreskin Melanocyte Primary Cells skin01		16_EnhW1	H3K4me1_Enh	H3K4me3_Pro			DNas
E061	Epithelial	SKIN.PEN.FRSK.MEL.03	Foreskin Melanocyte Primary Cells skin03	7_Enh	16_EnhW1	H3K4me1_Enh	H3K4me3_Pro	H3K27ac_Enh		
E057	Epithelial	SKIN.PEN.FRSK.KER.02	Foreskin Keratinocyte Primary Cells skin02		16_EnhW1	H3K4me1_Enh				DNas
E058	Epithelial	SKIN.PEN.FRSK.KER.03	Foreskin Keratinocyte Primary Cells skin03	7_Enh	16_EnhW1	H3K4me1_Enh	H3K4me3_Pro			
E028	Epithelial	BRST.HMEC.35	Breast variant Human Mammary Epithelial Cells (vHMEC)	7_Enh	16_EnhW1	H3K4me1_Enh				DNas
E027	Epithelial	BRST.MYO	Breast Myoepithelial Primary Cells		19_DNase	H3K4me1_Enh			H3K9ac_Pro	
E054	Neurosph	BRN.GANGEM.DR.NRSPHR	Ganglion Eminence derived primary cultured neurospheres	7_Enh	16_EnhW1	H3K4me1_Enh	H3K4me3_Pro			
E053	Neurosph	BRN.CRTX.DR.NRSPHR	Cortex derived primary cultured neurospheres	7_Enh	16_EnhW1	H3K4me1_Enh				
E112	Thymus	THYM	Thymus							
E093	Thymus	THYM.FET	Fetal Thymus							
E071	Brain	BRN.HIPP.MID	Brain Hippocampus Middle	7_Enh	14_EnhA2	H3K4me1_Enh	H3K4me3_Pro			
E074	Brain	BRN.SUB.NIG	Brain Substantia Nigra	7_Enh	16_EnhW1	H3K4me1_Enh	H3K4me3_Pro	H3K27ac_Enh	H3K9ac_Pro	
E068	Brain	BRN.ANT.CAUD	Brain Anterior Caudate	2_TssAFlnk	13_EnhA1	H3K4me1_Enh	H3K4me3_Pro		H3K9ac_Pro	
E069	Brain	BRN.CING.GYR	Brain Cingulate Gyrus	7_Enh	2_PromU	H3K4me1_Enh	H3K4me3_Pro	H3K27ac_Enh	H3K9ac_Pro	
E072	Brain	BRN.INF.TMP	Brain Inferior Temporal Lobe		16_EnhW1	H3K4me1_Enh	H3K4me3_Pro	H3K27ac_Enh	H3K9ac_Pro	
E067	Brain	BRN.ANG.GYR	Brain Angular Gyrus	7_Enh	16_EnhW1	H3K4me1_Enh	H3K4me3_Pro	H3K27ac_Enh	H3K9ac_Pro	
E073	Brain	BRN.DL.PRFRNTL.CRTX	Brain_Dorsolateral_Prefrontal_Cortex		16_EnhW1	H3K4me1_Enh	H3K4me3_Pro		H3K9ac_Pro	
E070	Brain	BRN.GRM.MTRX	Brain Germinal Matrix	7_Enh	16_EnhW1	H3K4me1_Enh				
E082	Brain	BRN.FET.F	Fetal Brain Female	7_Enh	16_EnhW1	H3K4me1_Enh				
E081	Brain	BRN.FET.M	Fetal Brain Male		16_EnhW1					
E063	Adipose	FAT.ADIP.NUC	Adipose Nuclei	7_Enh	16_EnhW1	H3K4me1_Enh		H3K27ac_Enh		
E100	Muscle	MUS.PSOAS	Psoas Muscle		19_DNase	H3K4me1_Enh				DNas
E108	Muscle	MUS.SKLT.F	Skeletal Muscle Female	7_Enh	16_EnhW1				H3K9ac_Pro	
E107	Muscle	MUS.SKLT.M	Skeletal Muscle Male		16_EnhW1				H3K9ac_Pro	
E089	Muscle	MUS.TRNK.FET	Fetal Muscle Trunk							
E090	Muscle	MUS.LEG.FET	Fetal Muscle Leg		19_DNase					DNas
E083	Heart	HRT.FET	Fetal Heart	7_Enh	14_EnhA2	H3K4me1_Enh	H3K4me3_Pro		H3K9ac_Pro	DNas
E104	Heart	HRT.ATR.R	Right Atrium		19_DNase					
E095	Heart	HRT.VENT.L	Left Ventricle		19_DNase			H3K27ac_Enh		
E105	Heart	HRT.VNT.R	Right Ventricle		16_EnhW1					
E065	Heart	VAS.AOR	Aorta		19_DNase					
E078	Sm. Muscle	GI.DUO.SM.MUS	Duodenum Smooth Muscle		16_EnhW1		H3K4me3_Pro			
E076	Sm. Muscle	GI.CLN.SM.MUS	Colon Smooth Muscle	7_Enh	16_EnhW1	H3K4me1_Enh	H3K4me3_Pro		H3K9ac_Pro	
E103	Sm. Muscle	GI.RECT.SM.MUS	Rectal Smooth Muscle	2_TssAFlnk	16_EnhW1	H3K4me1_Enh	H3K4me3_Pro			
E111	Sm. Muscle	GI.STMC.MUS	Stomach Smooth Muscle		16_EnhW1	H3K4me1_Enh	H3K4me3_Pro		H3K9ac_Pro	
E092	Digestive	GI.STMC.FET	Fetal Stomach		16_EnhW1	H3K4me1_Enh		H3K27ac_Enh		DNas
E085	Digestive	GI.S.INT.FET	Fetal Intestine Small	7_Enh	14_EnhA2	H3K4me1_Enh	H3K4me3_Pro	H3K27ac_Enh		DNas
E084	Digestive	GI.L.INT.FET	Fetal Intestine Large		13_EnhA1	H3K4me1_Enh		H3K27ac_Enh		DNas
E109	Digestive	GI.S.INT	Small Intestine		19_DNase			H3K27ac_Enh		
E106	Digestive	GI.CLN.SIG	Sigmoid Colon		19_DNase					
E075	Digestive	GI.CLN.MUC	Colonic Mucosa		19_DNase	H3K4me1_Enh				

E101	Digestive	GI.RECT.MUC.29	Rectal Mucosa Donor 29	7_Enh	16_EnhW1	H3K4me1_Enh	H3K4me3_Pro			
E102	Digestive	GI.RECT.MUC.31	Rectal Mucosa Donor 31	7_Enh	14_EnhA2	H3K4me1_Enh			H3K9ac_Pro	
E110	Digestive	GI.STMC.MUC	Stomach Mucosa	7_Enh	14_EnhA2	H3K4me1_Enh			H3K9ac_Pro	
E077	Digestive	GI.DUO.MUC	Duodenum Mucosa	2_TssAFlnk	14_EnhA2	H3K4me1_Enh	H3K4me3_Pro		H3K9ac_Pro	
E079	Digestive	GI.ESO	Esophagus		19_DNase					
E094	Digestive	GI.STMC.GAST	Gastric		19_DNase					
E099	Other	PLCNT.AMN	Placenta Amnion		19_DNase					
E086	Other	KID.FET	Fetal Kidney	7_Enh	16_EnhW1	H3K4me1_Enh				
E088	Other	LNG.FET	Fetal Lung	7_Enh	13_EnhA1	H3K4me1_Enh	H3K4me3_Pro		H3K9ac_Pro	
E097	Other	OVRY	Ovary		19_DNase	H3K4me1_Enh				
E087	Other	PANC.ISLT	Pancreatic Islets	1_TssA	16_EnhW1	H3K4me1_Enh	H3K4me3_Pro	H3K27ac_Enh		
E080	Other	ADRL.GLND.FET	Fetal Adrenal Gland	7_Enh	19_DNase	H3K4me1_Enh				
E091	Other	PLCNT.FET	Placenta		19_DNase					
E066	Other	LIV.ADLT	Liver	7_Enh	16_EnhW1	H3K4me1_Enh				
E098	Other	PANC	Pancreas		19_DNase			H3K27ac_Enh		DNas
E096	Other	LNG	Lung		19_DNase					
E113	Other	SPLN	Spleen		19_DNase					
E114	ENCODE2012	LNG.A549.ETOH002.CNCR	A549 EtOH 0.02pct Lung Carcinoma Cell Line	7_Enh	16_EnhW1	H3K4me1_Enh				
E115	ENCODE2012	BLD.DND41.CNCR	Dnd41 TCell Leukemia Cell Line							
E116	ENCODE2012	BLD.GM12878	GM12878 Lymphoblastoid Cells							
E117	ENCODE2012	CRVX.HELAS3.CNCR	HeLa-S3 Cervical Carcinoma Cell Line	2_TssAFlnk	13_EnhA1	H3K4me1_Enh	H3K4me3_Pro			DNas
E118	ENCODE2012	LIV.HEPG2.CNCR	HepG2 Hepatocellular Carcinoma Cell Line	12_EnhBiv	18_EnhAc					
E119	ENCODE2012	BRST.HMEC	HMEC Mammary Epithelial Primary Cells		16_EnhW1					
E120	ENCODE2012	MUS.HSMM	HSMM Skeletal Muscle Myoblasts Cells	7_Enh	16_EnhW1	H3K4me1_Enh				DNas
E121	ENCODE2012	MUS.HSMMT	HSMM cell derived Skeletal Muscle Myotubes Cells		16_EnhW1	H3K4me1_Enh				DNas
E122	ENCODE2012	VAS.HUVEC	HUVEC Umbilical Vein Endothelial Primary Cells		16_EnhW1	H3K4me1_Enh				
E123	ENCODE2012	BLD.K562.CNCR	K562 Leukemia Cells		17_EnhW2					
E124	ENCODE2012	BLD.CD14.MONO	Monocytes-CD14+ RO01746 Primary Cells	7_Enh		H3K4me1_Enh				
E125	ENCODE2012	BRN.NHA	NH-A Astrocytes Primary Cells	7_Enh	16_EnhW1	H3K4me1_Enh	H3K4me3_Pro		H3K9ac_Pro	DNas
E126	ENCODE2012	SKIN.NHDFAD	NHDF-Ad Adult Dermal Fibroblast Primary Cells	7_Enh	16_EnhW1	H3K4me1_Enh				
E127	ENCODE2012	SKIN.NHEK	NHEK-Epidermal Keratinocyte Primary Cells		16_EnhW1	H3K4me1_Enh				
E128	ENCODE2012	LNG.NHLF	NHLF Lung Fibroblast Primary Cells		22_PromP	H3K4me1_Enh				DNas
E129	ENCODE2012	BONE.OSTEO	Osteoblast Primary Cells	7_Enh	16_EnhW1	H3K4me1_Enh				

Proteins bound in ChIP-Seq experiments (ENCODE Project Consortium, 2011)

Cell ID	Protein
HEK293(b)	KAP1
HeLa-S3	CEBPB
U2OS	KAP1

Regulatory motifs altered

Position Weight Matrix ID (Library from Kheradpour and Kellis, 2013)	Strand	Ref	Alt	Match on:<br>Ref: CTGTGACAAAAATAATGATTCAAATTGACTGAGCAACGTATTGAGTGACAAGAGTTGCG<br>Alt: CTGTGACAAAAATAATGATTCAAATTGACCGAGCAACGTATTGAGTGACAAGAGTTGCG
AP-1_disc4	-	6.2	-5.7	TYTCWNWWTKAST
Foxm1	+	12.3	0.4	ARHWDSWBT
MIF-1	-	3.3	5.3	SMNGTTRCYWWGSAMCNN
Maf_disc1	-	6.6	-5.4	TGASTCAKCA
Maf_known1	+	11.5	8.2	HTGMCTCMRSM
Pou2f2_known2	+	13.7	14.1	DHDNDNDWATKYWWAWWHWWHSW

Detail view for rs851436

Link to dbSNP entry

Link to Ensembl Variation entry

Sequence facts

chr	pos (hg19)	chr	pos (hg38)	Reference	Alternate	1000 Genomes Phase 1 Frequencies				Sequence constraint		dbSNP functional annotation
						AFR	AMR	ASN	EUR	by GERP	by SiPhy	
chr2	19483101	chr2	19283340	C	A	0.62	0.35	0.4	0.33	No	No	none

Closest annotated gene					
Source	Distance	Direction	ID/Link	Common name	Description
GENCODE	NA	Within gene	ENSG00000236204.1	AC092594.1	
RefSeq	5'	65087	NR_039914	MIR4757	microRNA 4757 [Source:HGNC Symbol;Acc:41746]

Regulatory chromatin states from DNAse and histone ChIP-Seq (Roadmap Epigenomics Consortium, 2015)

(Black = missing data)

Epigenome ID (EID)	Group	Mnemonic	Description	Chromatin states (Core 15-state model)	Chromatin states (25-state model using 12 imputed marks)	H3K4me1	H3K4me3	H3K27ac	H3K9ac	DNase
E017	IMR90	LNG.IMR90	IMR90 fetal lung fibroblasts Cell Line							
E002	ESC	ESC.WA7	ES-WA7 Cells							
E008	ESC	ESC.H9	H9 Cells							
E001	ESC	ESC.I3	ES-I3 Cells							
E015	ESC	ESC.HUES6	HUES6 Cells							
E014	ESC	ESC.HUES48	HUES48 Cells							
E016	ESC	ESC.HUES64	HUES64 Cells							
E003	ESC	ESC.H1	H1 Cells							
E024	ESC	ESC.4STAR	ES-UCSF4 Cells							
E020	iPSC	IPSC.20B	iPS-20b Cells							
E019	iPSC	IPSC.18	iPS-18 Cells							
E018	iPSC	IPSC.15b	iPS-15b Cells							
E021	iPSC	IPSC.DF.6.9	iPS DF 6.9 Cells							
E022	iPSC	IPSC.DF.19.11	iPS DF 19.11 Cells							
E007	ES-deriv	ESDR.H1.NEUR.PROG	H1 Derived Neuronal Progenitor Cultured Cells							
E009	ES-deriv	ESDR.H9.NEUR.PROG	H9 Derived Neuronal Progenitor Cultured Cells							
E010	ES-deriv	ESDR.H9.NEUR	H9 Derived Neuron Cultured Cells							
E013	ES-deriv	ESDR.CD56.MESO	hESC Derived CD56+ Mesoderm Cultured Cells							
E012	ES-deriv	ESDR.CD56.ECTO	hESC Derived CD56+ Ectoderm Cultured Cells							
E011	ES-deriv	ESDR.CD184.ENDO	hESC Derived CD184+ Endoderm Cultured Cells							
E004	ES-deriv	ESDR.H1.BMP4.MESO	H1 BMP4 Derived Mesendoderm Cultured Cells							
E005	ES-deriv	ESDR.H1.BMP4.TROP	H1 BMP4 Derived Trophoblast Cultured Cells	7_Enh		H3K4me1_Enh	H3K4me3_Pro	H3K27ac_Enh		
E006	ES-deriv	ESDR.H1.MSC	H1 Derived Mesenchymal Stem Cells							
E062	Blood & T-cell	BLD.PER.MONUC.PC	Primary mononuclear cells from�peripheral�blood							
E034	Blood & T-cell	BLD.CD3.PPC	Primary T cells from�peripheral�blood							
E045	Blood & T-cell	BLD.CD4.CD25I.CD127.TMEMPC	Primary T cells effector/memory enriched from peripheral blood							
E033	Blood & T-cell	BLD.CD3.CPC	Primary T cells from cord blood							
E044	Blood & T-cell	BLD.CD4.CD25.CD127M.TREGPC	Primary T regulatory cells from�peripheral�blood							
E043	Blood & T-cell	BLD.CD4.CD25M.TPC	Primary T helper cells from�peripheral�blood							
E039	Blood & T-cell	BLD.CD4.CD25M.CD45RA.NPC	Primary T helper naive cells from�peripheral�blood							
E041	Blood & T-cell	BLD.CD4.CD25M.IL17M.PL.TPC	Primary T helper cells PMA-I stimulated							
E042	Blood & T-cell	BLD.CD4.CD25M.IL17P.PL.TPC	Primary T helper 17 cells PMA-I stimulated							
E040	Blood & T-cell	BLD.CD4.CD25M.CD45RO.MPC	Primary T helper memory cells from peripheral blood 1							
E037	Blood & T-cell	BLD.CD4.MPC	Primary T helper memory cells from							

			peripheral blood 2							
E048	Blood & T-cell	BLD.CD8.MPC	Primary T CD8+ memory cells from peripheral blood							
E038	Blood & T-cell	BLD.CD4.NPC	Primary T helper naive cells from peripheral blood							
E047	Blood & T-cell	BLD.CD8.NPC	Primary T CD8+ naive cells from peripheral blood							
E029	HSC & B-cell	BLD.CD14.PC	Primary monocytes from�peripheral�blood							
E031	HSC & B-cell	BLD.CD19.CPC	Primary B cells from cord blood							
E035	HSC & B-cell	BLD.CD34.PC	Primary hematopoietic stem cells							
E051	HSC & B-cell	BLD.MOB.CD34.PC.M	Primary hematopoietic stem cells G-CSF-mobilized Male							
E050	HSC & B-cell	BLD.MOB.CD34.PC.F	Primary hematopoietic stem cells G-CSF-mobilized Female							
E036	HSC & B-cell	BLD.CD34.CC	Primary hematopoietic stem cells short term culture							
E032	HSC & B-cell	BLD.CD19.PPC	Primary B cells from peripheral blood							
E046	HSC & B-cell	BLD.CD56.PC	Primary Natural Killer cells from�peripheral�blood							
E030	HSC & B-cell	BLD.CD15.PC	Primary neutrophils from�peripheral�blood							
E026	Mesench	STRM.MRW.MSC	Bone Marrow Derived Cultured Mesenchymal Stem Cells							
E049	Mesench	STRM.CHON.MRW.DR.MSC	Mesenchymal Stem Cell Derived Chondrocyte Cultured Cells							
E025	Mesench	FAT.ADIP.DR.MSC	Adipose Derived Mesenchymal Stem Cell Cultured Cells							
E023	Mesench	FAT.MSC.DR.ADIP	Mesenchymal Stem Cell Derived Adipocyte Cultured Cells							
E052	Myosat	MUS.SAT	Muscle Satellite Cultured Cells							
E055	Epithelial	SKIN.PEN.FRSK.FIB.01	Foreskin Fibroblast Primary Cells skin01							
E056	Epithelial	SKIN.PEN.FRSK.FIB.02	Foreskin Fibroblast Primary Cells skin02							
E059	Epithelial	SKIN.PEN.FRSK.MEL.01	Foreskin Melanocyte Primary Cells skin01							
E061	Epithelial	SKIN.PEN.FRSK.MEL.03	Foreskin Melanocyte Primary Cells skin03							
E057	Epithelial	SKIN.PEN.FRSK.KER.02	Foreskin Keratinocyte Primary Cells skin02							
E058	Epithelial	SKIN.PEN.FRSK.KER.03	Foreskin Keratinocyte Primary Cells skin03							
E028	Epithelial	BRST.HMEC.35	Breast variant Human Mammary Epithelial Cells (vHMEC)							
E027	Epithelial	BRST.MYO	Breast Myoepithelial Primary Cells			H3K4me1_Enh				
E054	Neurosph	BRN.GANGEM.DR.NRSPHR	Ganglion Eminence derived primary cultured neurospheres							
E053	Neurosph	BRN.CRTX.DR.NRSPHR	Cortex derived primary cultured neurospheres							
E112	Thymus	THYM	Thymus							
E093	Thymus	THYM.FET	Fetal Thymus							
E071	Brain	BRN.HIPP.MID	Brain Hippocampus Middle							
E074	Brain	BRN.SUB.NIG	Brain Substantia Nigra							
E068	Brain	BRN.ANT.CAUD	Brain Anterior Caudate							
E069	Brain	BRN.CING.GYR	Brain Cingulate Gyrus							
E072	Brain	BRN.INF.TMP	Brain Inferior Temporal Lobe							
E067	Brain	BRN.ANG.GYR	Brain Angular Gyrus							
E073	Brain	BRN.DL.PRFRNTL.CRTX	Brain_Dorsolateral_Prefrontal_Cortex							
E070	Brain	BRN.GRM.MTRX	Brain Germinal Matrix							
E082	Brain	BRN.FET.F	Fetal Brain Female							
E081	Brain	BRN.FET.M	Fetal Brain Male							
E063	Adipose	FAT.ADIP.NUC	Adipose Nuclei							
E100	Muscle	MUS.PSOAS	Psoas Muscle							
E108	Muscle	MUS.SKLT.F	Skeletal Muscle Female							
E107	Muscle	MUS.SKLT.M	Skeletal Muscle Male							
E089	Muscle	MUS.TRNK.FET	Fetal Muscle Trunk							
E090	Muscle	MUS.LEG.FET	Fetal Muscle Leg							
E083	Heart	HRT.FET	Fetal Heart							
E104	Heart	HRT.ATR.R	Right Atrium							
E095	Heart	HRT.VENT.L	Left Ventricle							
E105	Heart	HRT.VNT.R	Right Ventricle							
E065	Heart	VAS.AOR	Aorta							
E078	Sm. Muscle	GI.DUO.SM.MUS	Duodenum Smooth Muscle							
E076	Sm. Muscle	GI.CLN.SM.MUS	Colon Smooth Muscle							
E103	Sm. Muscle	GI.RECT.SM.MUS	Rectal Smooth Muscle							
E111	Sm. Muscle	GI.STMC.MUS	Stomach Smooth Muscle							
E092	Digestive	GI.STMC.FET	Fetal Stomach							
E085	Digestive	GI.S.INT.FET	Fetal Intestine Small							
E084	Digestive	GI.L.INT.FET	Fetal Intestine Large							
E109	Digestive	GI.S.INT	Small Intestine							

E106	Digestive	GI.CLN.SIG	Sigmoid Colon							
E075	Digestive	GI.CLN.MUC	Colonic Mucosa							
E101	Digestive	GI.RECT.MUC.29	Rectal Mucosa Donor 29							
E102	Digestive	GI.RECT.MUC.31	Rectal Mucosa Donor 31							
E110	Digestive	GI.STMC.MUC	Stomach Mucosa							
E077	Digestive	GI.DUO.MUC	Duodenum Mucosa							
E079	Digestive	GI.ESO	Esophagus							
E094	Digestive	GI.STMC.GAST	Gastric							
E099	Other	PLCNT.AMN	Placenta Amnion			H3K4me1_Enh				
E086	Other	KID.FET	Fetal Kidney							
E088	Other	LNG.FET	Fetal Lung							
E097	Other	OVRY	Ovary							
E087	Other	PANC.ISLT	Pancreatic Islets							
E080	Other	ADRL.GLND.FET	Fetal Adrenal Gland							
E091	Other	PLCNT.FET	Placenta							
E066	Other	LIV.ADLT	Liver							
E098	Other	PANC	Pancreas							
E096	Other	LNG	Lung							
E113	Other	SPLN	Spleen							
E114	ENCODE2012	LNG.A549.ETOH002.CNCR	A549 EtOH 0.02pct Lung Carcinoma Cell Line							
E115	ENCODE2012	BLD.DND41.CNCR	Dnd41 TCell Leukemia Cell Line							
E116	ENCODE2012	BLD.GM12878	GM12878 Lymphoblastoid Cells							
E117	ENCODE2012	CRVX.HELAS3.CNCR	HeLa-S3 Cervical Carcinoma Cell Line							
E118	ENCODE2012	LIV.HEPG2.CNCR	HepG2 Hepatocellular Carcinoma Cell Line							
E119	ENCODE2012	BRST.HMEC	HMEC Mammary Epithelial Primary Cells							
E120	ENCODE2012	MUS.HSMM	HSMM Skeletal Muscle Myoblasts Cells							
E121	ENCODE2012	MUS.HSMMT	HSMM cell derived Skeletal Muscle Myotubes Cells							
E122	ENCODE2012	VAS.HUVEC	HUVEC Umbilical Vein Endothelial Primary Cells							
E123	ENCODE2012	BLD.K562.CNCR	K562 Leukemia Cells							
E124	ENCODE2012	BLD.CD14.MONO	Monocytes-CD14+ RO01746 Primary Cells							
E125	ENCODE2012	BRN.NHA	NH-A Astrocytes Primary Cells							
E126	ENCODE2012	SKIN.NHDFAD	NHDF-Ad Adult Dermal Fibroblast Primary Cells							
E127	ENCODE2012	SKIN.NHEK	NHEK-Epidermal Keratinocyte Primary Cells							
E128	ENCODE2012	LNG.NHLF	NHLF Lung Fibroblast Primary Cells							
E129	ENCODE2012	BONE.OSTEO	Osteoblast Primary Cells							

GRASP QTL hits

Trait	p-value	PMID
Serum ratio of (DSGEGDFXAEGGGVR*)/(gamma-glutamylisoleucine*)	8.70E-04	21886157

Regulatory motifs altered

Position Weight Matrix ID (Library from Kheradpour and Kellis, 2013)	Strand	Ref	Alt	Match on: Ref: TCCCCACTCAATACAAATTGCTATGACCCCAAAAATACCAATGGTCCAGAAGCAAAGTG Alt: TCCCCACTCAATACAAATTGCTATGACCCAAAAAATACCAATGGTCCAGAAGCAAAGTG
ERalpha-a_known3	-	10.8	13.7	NHMRKKYRHYKTGACCYDH
HMG-IY_2	+	6.4	11.7	HHRRRAAWWTSMMND
INSM1	-	11.7	-0.3	YRMCCCCWKRCM
RORalpha1_3	-	1.4	13.3	TGACCYANYWW
RREB-1_1	+	5.7	5.1	MCCCAMMMMMMMMM
RXRA_known3	-	14.8	8.5	NBNBDTGACCYCDHVHN

Detail view for rs9297283

Link to dbSNP entry

Link to Ensembl Variation entry

Sequence facts

chr	pos (hg19)	chr	pos (hg38)	Reference	Alternate	1000 Genomes Phase 1 Frequencies				Sequence constraint		dbSNP functional annotation
						AFR	AMR	ASN	EUR	by GERP	by SiPhy	
chr8	99166896	chr8	98154668	G	T	0.28	0.23	0.18	0.18	No	No	intronic

Closest annotated gene					
Source	Distance	Direction	ID/Link	Common name	Description
GENCODE	NA	Within gene	ENSG00000104356.6	POP1	processing of precursor 1, ribonuclease P/MRP subunit (S. cerevisiae) [Source:HGNC Symbol;Acc:30129]
RefSeq	NA	Within gene	NM_001145860	POP1	processing of precursor 1, ribonuclease P/MRP subunit (S. cerevisiae) [Source:HGNC Symbol;Acc:30129]

Regulatory chromatin states from DNAse and histone ChIP-Seq (Roadmap Epigenomics Consortium, 2015)

(Black = missing data)

Epigenome ID (EID)	Group	Mnemonic	Description	Chromatin states (Core 15-state model)	Chromatin states (25-state model using 12 imputed marks)	H3K4me1	H3K4me3	H3K27ac	H3K9ac	DNa
E017	IMR90	LNG.IMR90	IMR90 fetal lung fibroblasts Cell Line	6_EnhG		H3K4me1_Enh				
E002	ESC	ESC.WA7	ES-WA7 Cells							
E008	ESC	ESC.H9	H9 Cells						H3K9ac_Pro	
E001	ESC	ESC.I3	ES-I3 Cells							
E015	ESC	ESC.HUES6	HUES6 Cells							
E014	ESC	ESC.HUES48	HUES48 Cells							
E016	ESC	ESC.HUES64	HUES64 Cells							
E003	ESC	ESC.H1	H1 Cells					H3K27ac_Enh		
E024	ESC	ESC.4STAR	ES-UCSF4 Cells							
E020	iPSC	IPSC.20B	iPS-20b Cells							
E019	iPSC	IPSC.18	iPS-18 Cells							
E018	iPSC	IPSC.15b	iPS-15b Cells							
E021	iPSC	IPSC.DF.6.9	iPS DF 6.9 Cells					H3K27ac_Enh		DNa
E022	iPSC	IPSC.DF.19.11	iPS DF 19.11 Cells							
E007	ES-deriv	ESDR.H1.NEUR.PROG	H1 Derived Neuronal Progenitor Cultured Cells	6_EnhG		H3K4me1_Enh			H3K9ac_Pro	
E009	ES-deriv	ESDR.H9.NEUR.PROG	H9 Derived Neuronal Progenitor Cultured Cells							
E010	ES-deriv	ESDR.H9.NEUR	H9 Derived Neuron Cultured Cells							
E013	ES-deriv	ESDR.CD56.MESO	hESC Derived CD56+ Mesoderm Cultured Cells			H3K4me1_Enh		H3K27ac_Enh		
E012	ES-deriv	ESDR.CD56.ECTO	hESC Derived CD56+ Ectoderm Cultured Cells			H3K4me1_Enh		H3K27ac_Enh		
E011	ES-deriv	ESDR.CD184.ENDO	hESC Derived CD184+ Endoderm Cultured Cells			H3K4me1_Enh		H3K27ac_Enh		
E004	ES-deriv	ESDR.H1.BMP4.MESO	H1 BMP4 Derived Mesendoderm Cultured Cells							
E005	ES-deriv	ESDR.H1.BMP4.TROP	H1 BMP4 Derived Trophoblast Cultured Cells							
E006	ES-deriv	ESDR.H1.MSC	H1 Derived Mesenchymal Stem Cells	6_EnhG		H3K4me1_Enh				
E062	Blood & T-cell	BLD.PER.MONUC.PC	Primary mononuclear cells from�peripheral�blood							
E034	Blood & T-cell	BLD.CD3.PPC	Primary T cells from�peripheral�blood							
E045	Blood & T-cell	BLD.CD4.CD25I.CD127.TMEMPC	Primary T cells effector/memory enriched from peripheral blood							
E033	Blood & T-cell	BLD.CD3.CPC	Primary T cells from cord blood							
E044	Blood & T-cell	BLD.CD4.CD25.CD127M.TREGPC	Primary T regulatory cells from�peripheral�blood							
E043	Blood & T-cell	BLD.CD4.CD25M.TPC	Primary T helper cells from�peripheral�blood							
E039	Blood & T-cell	BLD.CD4.CD25M.CD45RA.NPC	Primary T helper naive cells from�peripheral�blood							
E041	Blood & T-cell	BLD.CD4.CD25M.IL17M.PL.TPC	Primary T helper cells PMA-I stimulated							
E042	Blood & T-cell	BLD.CD4.CD25M.IL17P.PL.TPC	Primary T helper 17 cells PMA-I stimulated							
E040	Blood & T-cell	BLD.CD4.CD25M.CD45RO.MPC	Primary T helper memory cells from peripheral blood 1							
E037	Blood & T-cell	BLD.CD4.MPC	Primary T helper memory cells from peripheral blood 2							

E048	Blood & T-cell	BLD.CD8.MPC	Primary T CD8+ memory cells from peripheral blood							
E038	Blood & T-cell	BLD.CD4.NPC	Primary T helper naive cells from peripheral blood							
E047	Blood & T-cell	BLD.CD8.NPC	Primary T CD8+ naive cells from peripheral blood							
E029	HSC & B-cell	BLD.CD14.PC	Primary monocytes from�peripheral�blood							
E031	HSC & B-cell	BLD.CD19.CPC	Primary B cells from cord blood							
E035	HSC & B-cell	BLD.CD34.PC	Primary hematopoietic stem cells							
E051	HSC & B-cell	BLD.MOB.CD34.PC.M	Primary hematopoietic stem cells G-CSF-mobilized Male							
E050	HSC & B-cell	BLD.MOB.CD34.PC.F	Primary hematopoietic stem cells G-CSF-mobilized Female							
E036	HSC & B-cell	BLD.CD34.CC	Primary hematopoietic stem cells short term culture							
E032	HSC & B-cell	BLD.CD19.PPC	Primary B cells from peripheral blood							
E046	HSC & B-cell	BLD.CD56.PC	Primary Natural Killer cells from�peripheral�blood							
E030	HSC & B-cell	BLD.CD15.PC	Primary neutrophils from�peripheral�blood							
E026	Mesench	STRM.MRW.MSC	Bone Marrow Derived Cultured Mesenchymal Stem Cells	7_Enh	10_TxEnh5	H3K4me1_Enh				
E049	Mesench	STRM.CHON.MRW.DR.MSC	Mesenchymal Stem Cell Derived Chondrocyte Cultured Cells	6_EnhG	10_TxEnh5	H3K4me1_Enh		H3K27ac_Enh	H3K9ac_Pro	
E025	Mesench	FAT.ADIP.DR.MSC	Adipose Derived Mesenchymal Stem Cell Cultured Cells	6_EnhG	10_TxEnh5	H3K4me1_Enh			H3K9ac_Pro	
E023	Mesench	FAT.MSC.DR.ADIP	Mesenchymal Stem Cell Derived Adipocyte Cultured Cells		12_TxEnhW	H3K4me1_Enh				
E052	Myosat	MUS.SAT	Muscle Satellite Cultured Cells	6_EnhG	10_TxEnh5	H3K4me1_Enh				
E055	Epithelial	SKIN.PEN.FRSK.FIB.01	Foreskin Fibroblast Primary Cells skin01	6_EnhG	10_TxEnh5	H3K4me1_Enh	H3K4me3_Pro	H3K27ac_Enh		
E056	Epithelial	SKIN.PEN.FRSK.FIB.02	Foreskin Fibroblast Primary Cells skin02	6_EnhG	10_TxEnh5	H3K4me1_Enh	H3K4me3_Pro	H3K27ac_Enh		
E059	Epithelial	SKIN.PEN.FRSK.MEL.01	Foreskin Melanocyte Primary Cells skin01							
E061	Epithelial	SKIN.PEN.FRSK.MEL.03	Foreskin Melanocyte Primary Cells skin03			H3K4me1_Enh				
E057	Epithelial	SKIN.PEN.FRSK.KER.02	Foreskin Keratinocyte Primary Cells skin02							
E058	Epithelial	SKIN.PEN.FRSK.KER.03	Foreskin Keratinocyte Primary Cells skin03							
E028	Epithelial	BRST.HMEC.35	Breast variant Human Mammary Epithelial Cells (vHMEC)							
E027	Epithelial	BRST.MYO	Breast Myoepithelial Primary Cells							
E054	Neurosph	BRN.GANGEM.DR.NRSPHR	Ganglion Eminence derived primary cultured neurospheres							
E053	Neurosph	BRN.CRTX.DR.NRSPHR	Cortex derived primary cultured neurospheres							
E112	Thymus	THYM	Thymus							
E093	Thymus	THYM.FET	Fetal Thymus							
E071	Brain	BRN.HIPP.MID	Brain Hippocampus Middle	7_Enh	10_TxEnh5	H3K4me1_Enh		H3K27ac_Enh		
E074	Brain	BRN.SUB.NIG	Brain Substantia Nigra	7_Enh	10_TxEnh5	H3K4me1_Enh		H3K27ac_Enh	H3K9ac_Pro	
E068	Brain	BRN.ANT.CAUD	Brain Anterior Caudate	2_TssAFlnk	10_TxEnh5	H3K4me1_Enh	H3K4me3_Pro	H3K27ac_Enh		
E069	Brain	BRN.CING.GYR	Brain Cingulate Gyrus	7_Enh	10_TxEnh5	H3K4me1_Enh		H3K27ac_Enh	H3K9ac_Pro	
E072	Brain	BRN.INF.TMP	Brain Inferior Temporal Lobe	7_Enh	9_TxReg	H3K4me1_Enh		H3K27ac_Enh	H3K9ac_Pro	
E067	Brain	BRN.ANG.GYR	Brain Angular Gyrus	7_Enh	10_TxEnh5	H3K4me1_Enh	H3K4me3_Pro	H3K27ac_Enh	H3K9ac_Pro	
E073	Brain	BRN.DL.PRFRNTL.CRTX	Brain_Dorsolateral_Prefrontal_Cortex	7_Enh	10_TxEnh5	H3K4me1_Enh		H3K27ac_Enh	H3K9ac_Pro	
E070	Brain	BRN.GRM.MTRX	Brain Germinal Matrix							
E082	Brain	BRN.FET.F	Fetal Brain Female							
E081	Brain	BRN.FET.M	Fetal Brain Male							
E063	Adipose	FAT.ADIP.NUC	Adipose Nuclei							
E100	Muscle	MUS.PSOAS	Psoas Muscle							
E108	Muscle	MUS.SKLT.F	Skeletal Muscle Female							
E107	Muscle	MUS.SKLT.M	Skeletal Muscle Male						H3K9ac_Pro	
E089	Muscle	MUS.TRNK.FET	Fetal Muscle Trunk							
E090	Muscle	MUS.LEG.FET	Fetal Muscle Leg							
E083	Heart	HRT.FET	Fetal Heart							
E104	Heart	HRT.ATR.R	Right Atrium							
E095	Heart	HRT.VENT.L	Left Ventricle							
E105	Heart	HRT.VNT.R	Right Ventricle			H3K4me1_Enh				
E065	Heart	VAS.AOR	Aorta							
E078	Sm. Muscle	GI.DUO.SM.MUS	Duodenum Smooth Muscle							
E076	Sm. Muscle	GI.CLN.SM.MUS	Colon Smooth Muscle					H3K27ac_Enh		
E103	Sm. Muscle	GI.RECT.SM.MUS	Rectal Smooth Muscle							
E111	Sm. Muscle	GI.STMC.MUS	Stomach Smooth Muscle					H3K27ac_Enh		
E092	Digestive	GI.STMC.FET	Fetal Stomach							
E085	Digestive	GI.S.INT.FET	Fetal Intestine Small							
E084	Digestive	GI.L.INT.FET	Fetal Intestine Large							
E109	Digestive	GI.S.INT	Small Intestine							
E106	Digestive	GI.CLN.SIG	Sigmoid Colon							

E075	Digestive	GI.CLN.MUC	Colonic Mucosa							
E101	Digestive	GI.RECT.MUC.29	Rectal Mucosa Donor 29							
E102	Digestive	GI.RECT.MUC.31	Rectal Mucosa Donor 31							
E110	Digestive	GI.STMC.MUC	Stomach Mucosa							
E077	Digestive	GI.DUO.MUC	Duodenum Mucosa							
E079	Digestive	GI.ESO	Esophagus							
E094	Digestive	GI.STMC.GAST	Gastric							
E099	Other	PLCNT.AMN	Placenta Amnion							
E086	Other	KID.FET	Fetal Kidney							
E088	Other	LNG.FET	Fetal Lung						H3K9ac_Pro	
E097	Other	OVRY	Ovary							
E087	Other	PANC.ISLT	Pancreatic Islets							
E080	Other	ADRL.GLND.FET	Fetal Adrenal Gland	6_EnhG		H3K4me1_Enh		H3K27ac_Enh		
E091	Other	PLCNT.FET	Placenta							
E066	Other	LIV.ADLT	Liver							
E098	Other	PANC	Pancreas	6_EnhG		H3K4me1_Enh				
E096	Other	LNG	Lung							
E113	Other	SPLN	Spleen				H3K4me3_Pro			
E114	ENCODE2012	LNG.A549.ETOH002.CNCR	A549 EtOH 0.02pct Lung Carcinoma Cell Line							
E115	ENCODE2012	BLD.DND41.CNCR	Dnd41 TCell Leukemia Cell Line					H3K27ac_Enh		
E116	ENCODE2012	BLD.GM12878	GM12878 Lymphoblastoid Cells							
E117	ENCODE2012	CRVX.HELAS3.CNCR	HeLa-S3 Cervical Carcinoma Cell Line							
E118	ENCODE2012	LIV.HEPG2.CNCR	HepG2 Hepatocellular Carcinoma Cell Line							
E119	ENCODE2012	BRST.HMEC	HMEC Mammary Epithelial Primary Cells							
E120	ENCODE2012	MUS.HSMM	HSMM Skeletal Muscle Myoblasts Cells	6_EnhG	12_TxEnhW	H3K4me1_Enh		H3K27ac_Enh		
E121	ENCODE2012	MUS.HSMMT	HSMM cell derived Skeletal Muscle Myotubes Cells		10_TxEnh5	H3K4me1_Enh		H3K27ac_Enh		DNa
E122	ENCODE2012	VAS.HUVEC	HUVEC Umbilical Vein Endothelial Primary Cells							
E123	ENCODE2012	BLD.K562.CNCR	K562 Leukemia Cells							
E124	ENCODE2012	BLD.CD14.MONO	Monocytes-CD14+ RO01746 Primary Cells							
E125	ENCODE2012	BRN.NHA	NH-A Astrocytes Primary Cells	6_EnhG	10_TxEnh5	H3K4me1_Enh		H3K27ac_Enh	H3K9ac_Pro	
E126	ENCODE2012	SKIN.NHDFAD	NHDF-Ad Adult Dermal Fibroblast Primary Cells		11_TxEnh3	H3K4me1_Enh		H3K27ac_Enh		
E127	ENCODE2012	SKIN.NHEK	NHEK-Epidermal Keratinocyte Primary Cells							
E128	ENCODE2012	LNG.NHLF	NHLF Lung Fibroblast Primary Cells		11_TxEnh3	H3K4me1_Enh		H3K27ac_Enh		
E129	ENCODE2012	BONE.OSTEO	Osteoblast Primary Cells	6_EnhG	10_TxEnh5	H3K4me1_Enh		H3K27ac_Enh		

Detail view for rs12940715

Link to dbSNP entry

Link to Ensembl Variation entry

Sequence facts

chr	pos (hg19)	chr	pos (hg38)	Reference	Alternate	1000 Genomes Phase 1 Frequencies				Sequence constraint		dbSNP functional annotation
						AFR	AMR	ASN	EUR	by GERP	by SiPhy	
chr17	71477731	chr17	73481592	C	T	0.01	0.13	0.05	0.2	No	No	intronic

Closest annotated gene					
Source	Distance	Direction	ID/Link	Common name	Description
GENCODE	NA	Within gene	ENSG00000069188.12	SDK2	sidekick cell adhesion molecule 2 [Source:HGNC Symbol;Acc:19308]
RefSeq	NA	Within gene	NM_001144952	SDK2	sidekick cell adhesion molecule 2 [Source:HGNC Symbol;Acc:19308]

Regulatory chromatin states from DNAse and histone ChIP-Seq (Roadmap Epigenomics Consortium, 2015)

(Black = missing data)

Epigenome ID (EID)	Group	Mnemonic	Description	Chromatin states (Core 15-state model)	Chromatin states (25-state model using 12 imputed marks)	H3K4me1	H3K4me3	H3K27ac	H3K9ac	DNas
E017	IMR90	LNG.IMR90	IMR90 fetal lung fibroblasts Cell Line							
E002	ESC	ESC.WA7	ES-WA7 Cells						H3K9ac_Pro	
E008	ESC	ESC.H9	H9 Cells							
E001	ESC	ESC.I3	ES-I3 Cells	7_Enh		H3K4me1_Enh				
E015	ESC	ESC.HUES6	HUES6 Cells							
E014	ESC	ESC.HUES48	HUES48 Cells							
E016	ESC	ESC.HUES64	HUES64 Cells							
E003	ESC	ESC.H1	H1 Cells							
E024	ESC	ESC.4STAR	ES-UCSF4 Cells			H3K4me1_Enh				
E020	iPSC	IPSC.20B	iPS-20b Cells							
E019	iPSC	IPSC.18	iPS-18 Cells							
E018	iPSC	IPSC.15b	iPS-15b Cells							
E021	iPSC	IPSC.DF.6.9	iPS DF 6.9 Cells							
E022	iPSC	IPSC.DF.19.11	iPS DF 19.11 Cells			H3K4me1_Enh				
E007	ES-deriv	ESDR.H1.NEUR.PROG	H1 Derived Neuronal Progenitor Cultured Cells							
E009	ES-deriv	ESDR.H9.NEUR.PROG	H9 Derived Neuronal Progenitor Cultured Cells			H3K4me1_Enh				
E010	ES-deriv	ESDR.H9.NEUR	H9 Derived Neuron Cultured Cells							
E013	ES-deriv	ESDR.CD56.MESO	hESC Derived CD56+ Mesoderm Cultured Cells			H3K4me1_Enh				
E012	ES-deriv	ESDR.CD56.ECTO	hESC Derived CD56+ Ectoderm Cultured Cells			H3K4me1_Enh		H3K27ac_Enh		
E011	ES-deriv	ESDR.CD184.ENDO	hESC Derived CD184+ Endoderm Cultured Cells					H3K27ac_Enh		
E004	ES-deriv	ESDR.H1.BMP4.MESO	H1 BMP4 Derived Mesendoderm Cultured Cells							
E005	ES-deriv	ESDR.H1.BMP4.TROP	H1 BMP4 Derived Trophoblast Cultured Cells							
E006	ES-deriv	ESDR.H1.MSC	H1 Derived Mesenchymal Stem Cells	7_Enh		H3K4me1_Enh				
E062	Blood & T-cell	BLD.PER.MONUC.PC	Primary mononuclear cells from�peripheral�blood							
E034	Blood & T-cell	BLD.CD3.PPC	Primary T cells from�peripheral�blood							
E045	Blood & T-cell	BLD.CD4.CD25I.CD127.TMEMPC	Primary T cells effector/memory enriched from peripheral blood							
E033	Blood & T-cell	BLD.CD3.CPC	Primary T cells from cord blood		17_EnhW2					
E044	Blood & T-cell	BLD.CD4.CD25.CD127M.TREGPC	Primary T regulatory cells from�peripheral�blood							
E043	Blood & T-cell	BLD.CD4.CD25M.TPC	Primary T helper cells from�peripheral�blood							
E039	Blood & T-cell	BLD.CD4.CD25M.CD45RA.NPC	Primary T helper naive cells from�peripheral�blood							
E041	Blood & T-cell	BLD.CD4.CD25M.IL17M.PL.TPC	Primary T helper cells PMA-I stimulated							
E042	Blood & T-cell	BLD.CD4.CD25M.IL17P.PL.TPC	Primary T helper 17 cells PMA-I stimulated							
E040	Blood & T-cell	BLD.CD4.CD25M.CD45RO.MPC	Primary T helper memory cells from peripheral blood 1							
E037	Blood & T-cell	BLD.CD4.MPC	Primary T helper memory cells from peripheral blood 2							

E048	Blood & T-cell	BLD.CD8.MPC	Primary T CD8+ memory cells from peripheral blood							
E038	Blood & T-cell	BLD.CD4.NPC	Primary T helper naive cells from peripheral blood							
E047	Blood & T-cell	BLD.CD8.NPC	Primary T CD8+ naive cells from peripheral blood							
E029	HSC & B-cell	BLD.CD14.PC	Primary monocytes from�peripheral�blood			H3K4me1_Enh				
E031	HSC & B-cell	BLD.CD19.CPC	Primary B cells from cord blood		17_EnhW2					
E035	HSC & B-cell	BLD.CD34.PC	Primary hematopoietic stem cells	7_Enh	17_EnhW2	H3K4me1_Enh				
E051	HSC & B-cell	BLD.MOB.CD34.PC.M	Primary hematopoietic stem cells G-CSF-mobilized Male	7_Enh	13_EnhA1	H3K4me1_Enh				
E050	HSC & B-cell	BLD.MOB.CD34.PC.F	Primary hematopoietic stem cells G-CSF-mobilized Female	7_Enh	13_EnhA1	H3K4me1_Enh		H3K27ac_Enh		
E036	HSC & B-cell	BLD.CD34.CC	Primary hematopoietic stem cells short term culture		17_EnhW2	H3K4me1_Enh				
E032	HSC & B-cell	BLD.CD19.PPC	Primary B cells from peripheral blood			H3K4me1_Enh				
E046	HSC & B-cell	BLD.CD56.PC	Primary Natural Killer cells from�peripheral�blood			H3K4me1_Enh				
E030	HSC & B-cell	BLD.CD15.PC	Primary neutrophils from�peripheral�blood		17_EnhW2					
E026	Mesench	STRM.MRW.MSC	Bone Marrow Derived Cultured Mesenchymal Stem Cells							
E049	Mesench	STRM.CHON.MRW.DR.MSC	Mesenchymal Stem Cell Derived Chondrocyte Cultured Cells							
E025	Mesench	FAT.ADIP.DR.MSC	Adipose Derived Mesenchymal Stem Cell Cultured Cells							
E023	Mesench	FAT.MSC.DR.ADIP	Mesenchymal Stem Cell Derived Adipocyte Cultured Cells							
E052	Myosat	MUS.SAT	Muscle Satellite Cultured Cells							
E055	Epithelial	SKIN.PEN.FRSK.FIB.01	Foreskin Fibroblast Primary Cells skin01							
E056	Epithelial	SKIN.PEN.FRSK.FIB.02	Foreskin Fibroblast Primary Cells skin02							
E059	Epithelial	SKIN.PEN.FRSK.MEL.01	Foreskin Melanocyte Primary Cells skin01							
E061	Epithelial	SKIN.PEN.FRSK.MEL.03	Foreskin Melanocyte Primary Cells skin03							
E057	Epithelial	SKIN.PEN.FRSK.KER.02	Foreskin Keratinocyte Primary Cells skin02							
E058	Epithelial	SKIN.PEN.FRSK.KER.03	Foreskin Keratinocyte Primary Cells skin03			H3K4me1_Enh				
E028	Epithelial	BRST.HMEC.35	Breast variant Human Mammary Epithelial Cells (vHMEC)							
E027	Epithelial	BRST.MYO	Breast Myoepithelial Primary Cells	6_EnhG	17_EnhW2	H3K4me1_Enh			H3K9ac_Pro	
E054	Neurosph	BRN.GANGEM.DR.NRSPHR	Ganglion Eminence derived primary cultured neurospheres		17_EnhW2					
E053	Neurosph	BRN.CRTX.DR.NRSPHR	Cortex derived primary cultured neurospheres		17_EnhW2					
E112	Thymus	THYM	Thymus							
E093	Thymus	THYM.FET	Fetal Thymus							
E071	Brain	BRN.HIPP.MID	Brain Hippocampus Middle			H3K4me1_Enh		H3K27ac_Enh		
E074	Brain	BRN.SUB.NIG	Brain Substantia Nigra							
E068	Brain	BRN.ANT.CAUD	Brain Anterior Caudate			H3K4me1_Enh				
E069	Brain	BRN.CING.GYR	Brain Cingulate Gyrus		17_EnhW2	H3K4me1_Enh			H3K9ac_Pro	
E072	Brain	BRN.INF.TMP	Brain Inferior Temporal Lobe	7_Enh	23_PromBiv	H3K4me1_Enh		H3K27ac_Enh	H3K9ac_Pro	
E067	Brain	BRN.ANG.GYR	Brain Angular Gyrus		17_EnhW2	H3K4me1_Enh		H3K27ac_Enh	H3K9ac_Pro	
E073	Brain	BRN.DL.PRFRNTL.CRTX	Brain_Dorsolateral_Prefrontal_Cortex	7_Enh	23_PromBiv	H3K4me1_Enh		H3K27ac_Enh	H3K9ac_Pro	
E070	Brain	BRN.GRM.MTRX	Brain Germinal Matrix		17_EnhW2	H3K4me1_Enh				
E082	Brain	BRN.FET.F	Fetal Brain Female	7_Enh	17_EnhW2	H3K4me1_Enh				
E081	Brain	BRN.FET.M	Fetal Brain Male	7_Enh	18_EnhAc	H3K4me1_Enh				
E063	Adipose	FAT.ADIP.NUC	Adipose Nuclei							
E100	Muscle	MUS.PSOAS	Psoas Muscle							
E108	Muscle	MUS.SKLT.F	Skeletal Muscle Female							
E107	Muscle	MUS.SKLT.M	Skeletal Muscle Male							
E089	Muscle	MUS.TRNK.FET	Fetal Muscle Trunk			H3K4me1_Enh				
E090	Muscle	MUS.LEG.FET	Fetal Muscle Leg	7_Enh		H3K4me1_Enh				
E083	Heart	HRT.FET	Fetal Heart	7_Enh	14_EnhA2	H3K4me1_Enh	H3K4me3_Pro		H3K9ac_Pro	
E104	Heart	HRT.ATR.R	Right Atrium					H3K27ac_Enh		
E095	Heart	HRT.VENT.L	Left Ventricle	7_Enh	17_EnhW2	H3K4me1_Enh		H3K27ac_Enh		
E105	Heart	HRT.VNT.R	Right Ventricle	7_Enh	17_EnhW2	H3K4me1_Enh		H3K27ac_Enh		
E065	Heart	VAS.AOR	Aorta							
E078	Sm. Muscle	GI.DUO.SM.MUS	Duodenum Smooth Muscle							
E076	Sm. Muscle	GI.CLN.SM.MUS	Colon Smooth Muscle							
E103	Sm. Muscle	GI.RECT.SM.MUS	Rectal Smooth Muscle							
E111	Sm. Muscle	GI.STMC.MUS	Stomach Smooth Muscle							
E092	Digestive	GI.STMC.FET	Fetal Stomach			H3K4me1_Enh				
E085	Digestive	GI.S.INT.FET	Fetal Intestine Small							
E084	Digestive	GI.L.INT.FET	Fetal Intestine Large							
E109	Digestive	GI.S.INT	Small Intestine							
E106	Digestive	GI.CLN.SIG	Sigmoid Colon							

E075	Digestive	GI.CLN.MUC	Colonic Mucosa							
E101	Digestive	GI.RECT.MUC.29	Rectal Mucosa Donor 29							
E102	Digestive	GI.RECT.MUC.31	Rectal Mucosa Donor 31							
E110	Digestive	GI.STMC.MUC	Stomach Mucosa							
E077	Digestive	GI.DUO.MUC	Duodenum Mucosa							
E079	Digestive	GI.ESO	Esophagus							
E094	Digestive	GI.STMC.GAST	Gastric							
E099	Other	PLCNT.AMN	Placenta Amnion							
E086	Other	KID.FET	Fetal Kidney							
E088	Other	LNG.FET	Fetal Lung							
E097	Other	OVRY	Ovary			H3K4me1_Enh				
E087	Other	PANC.ISLT	Pancreatic Islets							
E080	Other	ADRL.GLND.FET	Fetal Adrenal Gland							
E091	Other	PLCNT.FET	Placenta							
E066	Other	LIV.ADLT	Liver							
E098	Other	PANC	Pancreas							
E096	Other	LNG	Lung							
E113	Other	SPLN	Spleen			H3K4me1_Enh	H3K4me3_Pro			
E114	ENCODE2012	LNG.A549.ETOH002.CNCR	A549 EtOH 0.02pct Lung Carcinoma Cell Line							
E115	ENCODE2012	BLD.DND41.CNCR	Dnd41 TCell Leukemia Cell Line		17_EnhW2			H3K27ac_Enh		
E116	ENCODE2012	BLD.GM12878	GM12878 Lymphoblastoid Cells							
E117	ENCODE2012	CRVX.HELAS3.CNCR	HeLa-S3 Cervical Carcinoma Cell Line							
E118	ENCODE2012	LIV.HEPG2.CNCR	HepG2 Hepatocellular Carcinoma Cell Line							
E119	ENCODE2012	BRST.HMEC	HMEC Mammary Epithelial Primary Cells							
E120	ENCODE2012	MUS.HSMM	HSMM Skeletal Muscle Myoblasts Cells							
E121	ENCODE2012	MUS.HSMMT	HSMM cell derived Skeletal Muscle Myotubes Cells			H3K4me1_Enh		H3K27ac_Enh		
E122	ENCODE2012	VAS.HUVEC	HUVEC Umbilical Vein Endothelial Primary Cells							
E123	ENCODE2012	BLD.K562.CNCR	K562 Leukemia Cells							
E124	ENCODE2012	BLD.CD14.MONO	Monocytes-CD14+ RO01746 Primary Cells		17_EnhW2	H3K4me1_Enh			H3K9ac_Pro	
E125	ENCODE2012	BRN.NHA	NH-A Astrocytes Primary Cells			H3K4me1_Enh				
E126	ENCODE2012	SKIN.NHDFAD	NHDF-Ad Adult Dermal Fibroblast Primary Cells							
E127	ENCODE2012	SKIN.NHEK	NHEK-Epidermal Keratinocyte Primary Cells							
E128	ENCODE2012	LNG.NHLF	NHLF Lung Fibroblast Primary Cells							
E129	ENCODE2012	BONE.OSTEO	Osteoblast Primary Cells							

Regulatory motifs altered

**Position Weight Matrix ID (Library from Kheradpour and Kellis, 2013)**	**Strand**	**Ref**	**Alt**	**Match on:** Ref: GAGACAGAGTGGTGAAAGCCCTCTCCCCTCGCAGGACGTAGCCGGATCCCTCACCAACG Alt: GAGACAGAGTGGTGAAAGCCCTCTCCCCTTGCAGGACGTAGCCGGATCCCTCACCAACG
BDP1_disc1	-	-12.4	-0.5	CCTCCTCCAGGMAGYCYKCCNKG
BDP1_disc3	-	12.4	12.2	SYCHSWSCYHNNNS
ERalpha-a_disc4	-	9.9	10.7	SHBSNSNSNSCHNS
GR_disc6	-	10.9	10	SHBNSHSBBCHBNS
RXRA_known6	-	12.3	11.9	TSMCCTYTSHCCYRS
ZBTB33_known1	-	12.4	10	HTVGCAGGAD

Detail view for rs1805453

Link to dbSNP entry

Link to Ensembl Variation entry

Sequence facts

chr	pos (hg19)	chr	pos (hg38)	Reference	Alternate	1000 Genomes Phase 1 Frequencies				Sequence constraint		dbSNP functional annotation
						AFR	AMR	ASN	EUR	by GERP	by SiPhy	
chr17	5385398	chr17	5482078	C	A	0.55	0.13	0.3	0.19	No	No	intronic

Closest annotated gene					
Source	Distance	Direction	ID/Link	Common name	Description
GENCODE	NA	Within gene	ENSG00000072849.6	DERL2	derlin 2 [Source:HGNC Symbol;Acc:17943]
RefSeq	NA	Within gene	NM_016041	DERL2	derlin 2 [Source:HGNC Symbol;Acc:17943]

Regulatory chromatin states from DNAse and histone ChIP-Seq (Roadmap Epigenomics Consortium, 2015)

(Black = missing data)

Epigenome ID (EID)	Group	Mnemonic	Description	Chromatin states (Core 15-state model)	Chromatin states (25-state model using 12 imputed marks)	H3K4me1	H3K4me3	H3K27ac	H3K9ac	DNas
E017	IMR90	LNG.IMR90	IMR90 fetal lung fibroblasts Cell Line							
E002	ESC	ESC.WA7	ES-WA7 Cells							
E008	ESC	ESC.H9	H9 Cells							
E001	ESC	ESC.I3	ES-I3 Cells							
E015	ESC	ESC.HUES6	HUES6 Cells							
E014	ESC	ESC.HUES48	HUES48 Cells							
E016	ESC	ESC.HUES64	HUES64 Cells							
E003	ESC	ESC.H1	H1 Cells							
E024	ESC	ESC.4STAR	ES-UCSF4 Cells							
E020	iPSC	IPSC.20B	iPS-20b Cells						H3K9ac_Pro	
E019	iPSC	IPSC.18	iPS-18 Cells							
E018	iPSC	IPSC.15b	iPS-15b Cells							
E021	iPSC	IPSC.DF.6.9	iPS DF 6.9 Cells							
E022	iPSC	IPSC.DF.19.11	iPS DF 19.11 Cells							
E007	ES-deriv	ESDR.H1.NEUR.PROG	H1 Derived Neuronal Progenitor Cultured Cells							
E009	ES-deriv	ESDR.H9.NEUR.PROG	H9 Derived Neuronal Progenitor Cultured Cells							
E010	ES-deriv	ESDR.H9.NEUR	H9 Derived Neuron Cultured Cells							
E013	ES-deriv	ESDR.CD56.MESO	hESC Derived CD56+ Mesoderm Cultured Cells			H3K4me1_Enh				
E012	ES-deriv	ESDR.CD56.ECTO	hESC Derived CD56+ Ectoderm Cultured Cells					H3K27ac_Enh		
E011	ES-deriv	ESDR.CD184.ENDO	hESC Derived CD184+ Endoderm Cultured Cells							
E004	ES-deriv	ESDR.H1.BMP4.MESO	H1 BMP4 Derived Mesendoderm Cultured Cells							
E005	ES-deriv	ESDR.H1.BMP4.TROP	H1 BMP4 Derived Trophoblast Cultured Cells							
E006	ES-deriv	ESDR.H1.MSC	H1 Derived Mesenchymal Stem Cells							
E062	Blood & T-cell	BLD.PER.MONUC.PC	Primary mononuclear cells from�peripheral�blood						H3K9ac_Pro	
E034	Blood & T-cell	BLD.CD3.PPC	Primary T cells from�peripheral�blood							
E045	Blood & T-cell	BLD.CD4.CD25I.CD127.TMEMPC	Primary T cells effector/memory enriched from peripheral blood							
E033	Blood & T-cell	BLD.CD3.CPC	Primary T cells from cord blood							
E044	Blood & T-cell	BLD.CD4.CD25.CD127M.TREGPC	Primary T regulatory cells from�peripheral�blood			H3K4me1_Enh		H3K27ac_Enh		
E043	Blood & T-cell	BLD.CD4.CD25M.TPC	Primary T helper cells from�peripheral�blood			H3K4me1_Enh		H3K27ac_Enh		
E039	Blood & T-cell	BLD.CD4.CD25M.CD45RA.NPC	Primary T helper naive cells from�peripheral�blood			H3K4me1_Enh		H3K27ac_Enh		
E041	Blood & T-cell	BLD.CD4.CD25M.IL17M.PL.TPC	Primary T helper cells PMA-I stimulated			H3K4me1_Enh		H3K27ac_Enh		
E042	Blood & T-cell	BLD.CD4.CD25M.IL17P.PL.TPC	Primary T helper 17 cells PMA-I stimulated			H3K4me1_Enh		H3K27ac_Enh		
E040	Blood & T-cell	BLD.CD4.CD25M.CD45RO.MPC	Primary T helper memory cells from peripheral blood 1					H3K27ac_Enh		
E037	Blood & T-cell	BLD.CD4.MPC	Primary T helper memory cells from peripheral blood 2			H3K4me1_Enh		H3K27ac_Enh		

E048	Blood & T-cell	BLD.CD8.MPC	Primary T CD8+ memory cells from peripheral blood							
E038	Blood & T-cell	BLD.CD4.NPC	Primary T helper naive cells from peripheral blood			H3K4me1_Enh			H3K9ac_Pro	
E047	Blood & T-cell	BLD.CD8.NPC	Primary T CD8+ naive cells from peripheral blood			H3K4me1_Enh				
E029	HSC & B-cell	BLD.CD14.PC	Primary monocytes from�peripheral�blood							
E031	HSC & B-cell	BLD.CD19.CPC	Primary B cells from cord blood							
E035	HSC & B-cell	BLD.CD34.PC	Primary hematopoietic stem cells							
E051	HSC & B-cell	BLD.MOB.CD34.PC.M	Primary hematopoietic stem cells G-CSF-mobilized Male							
E050	HSC & B-cell	BLD.MOB.CD34.PC.F	Primary hematopoietic stem cells G-CSF-mobilized Female					H3K27ac_Enh		
E036	HSC & B-cell	BLD.CD34.CC	Primary hematopoietic stem cells short term culture							
E032	HSC & B-cell	BLD.CD19.PPC	Primary B cells from peripheral blood							
E046	HSC & B-cell	BLD.CD56.PC	Primary Natural Killer cells from�peripheral�blood							
E030	HSC & B-cell	BLD.CD15.PC	Primary neutrophils from�peripheral�blood							
E026	Mesench	STRM.MRW.MSC	Bone Marrow Derived Cultured Mesenchymal Stem Cells							
E049	Mesench	STRM.CHON.MRW.DR.MSC	Mesenchymal Stem Cell Derived Chondrocyte Cultured Cells						H3K9ac_Pro	
E025	Mesench	FAT.ADIP.DR.MSC	Adipose Derived Mesenchymal Stem Cell Cultured Cells							
E023	Mesench	FAT.MSC.DR.ADIP	Mesenchymal Stem Cell Derived Adipocyte Cultured Cells							
E052	Myosat	MUS.SAT	Muscle Satellite Cultured Cells							
E055	Epithelial	SKIN.PEN.FRSK.FIB.01	Foreskin Fibroblast Primary Cells skin01			H3K4me1_Enh		H3K27ac_Enh		
E056	Epithelial	SKIN.PEN.FRSK.FIB.02	Foreskin Fibroblast Primary Cells skin02			H3K4me1_Enh				
E059	Epithelial	SKIN.PEN.FRSK.MEL.01	Foreskin Melanocyte Primary Cells skin01							
E061	Epithelial	SKIN.PEN.FRSK.MEL.03	Foreskin Melanocyte Primary Cells skin03					H3K27ac_Enh		
E057	Epithelial	SKIN.PEN.FRSK.KER.02	Foreskin Keratinocyte Primary Cells skin02							
E058	Epithelial	SKIN.PEN.FRSK.KER.03	Foreskin Keratinocyte Primary Cells skin03	6_EnhG		H3K4me1_Enh		H3K27ac_Enh		
E028	Epithelial	BRST.HMEC.35	Breast variant Human Mammary Epithelial Cells (vHMEC)			H3K4me1_Enh				
E027	Epithelial	BRST.MYO	Breast Myoepithelial Primary Cells			H3K4me1_Enh				
E054	Neurosph	BRN.GANGEM.DR.NRSPHR	Ganglion Eminence derived primary cultured neurospheres							
E053	Neurosph	BRN.CRTX.DR.NRSPHR	Cortex derived primary cultured neurospheres							
E112	Thymus	THYM	Thymus							
E093	Thymus	THYM.FET	Fetal Thymus							
E071	Brain	BRN.HIPP.MID	Brain Hippocampus Middle							
E074	Brain	BRN.SUB.NIG	Brain Substantia Nigra					H3K27ac_Enh		
E068	Brain	BRN.ANT.CAUD	Brain Anterior Caudate							
E069	Brain	BRN.CING.GYR	Brain Cingulate Gyrus							
E072	Brain	BRN.INF.TMP	Brain Inferior Temporal Lobe							
E067	Brain	BRN.ANG.GYR	Brain Angular Gyrus			H3K4me1_Enh			H3K9ac_Pro	
E073	Brain	BRN.DL.PRFRNTL.CRTX	Brain_Dorsolateral_Prefrontal_Cortex			H3K4me1_Enh				
E070	Brain	BRN.GRM.MTRX	Brain Germinal Matrix							
E082	Brain	BRN.FET.F	Fetal Brain Female							
E081	Brain	BRN.FET.M	Fetal Brain Male							
E063	Adipose	FAT.ADIP.NUC	Adipose Nuclei						H3K9ac_Pro	
E100	Muscle	MUS.PSOAS	Psoas Muscle							
E108	Muscle	MUS.SKLT.F	Skeletal Muscle Female							
E107	Muscle	MUS.SKLT.M	Skeletal Muscle Male							
E089	Muscle	MUS.TRNK.FET	Fetal Muscle Trunk							
E090	Muscle	MUS.LEG.FET	Fetal Muscle Leg							
E083	Heart	HRT.FET	Fetal Heart							
E104	Heart	HRT.ATR.R	Right Atrium							
E095	Heart	HRT.VENT.L	Left Ventricle							
E105	Heart	HRT.VNT.R	Right Ventricle							
E065	Heart	VAS.AOR	Aorta							
E078	Sm. Muscle	GI.DUO.SM.MUS	Duodenum Smooth Muscle			H3K4me1_Enh				
E076	Sm. Muscle	GI.CLN.SM.MUS	Colon Smooth Muscle							
E103	Sm. Muscle	GI.RECT.SM.MUS	Rectal Smooth Muscle							
E111	Sm. Muscle	GI.STMC.MUS	Stomach Smooth Muscle						H3K9ac_Pro	
E092	Digestive	GI.STMC.FET	Fetal Stomach							
E085	Digestive	GI.S.INT.FET	Fetal Intestine Small							
E084	Digestive	GI.L.INT.FET	Fetal Intestine Large							
E109	Digestive	GI.S.INT	Small Intestine							
E106	Digestive	GI.CLN.SIG	Sigmoid Colon							
E075	Digestive	GI.CLN.MUC	Colonic Mucosa							

E101	Digestive	GI.RECT.MUC.29	Rectal Mucosa Donor 29							
E102	Digestive	GI.RECT.MUC.31	Rectal Mucosa Donor 31							
E110	Digestive	GI.STMC.MUC	Stomach Mucosa							
E077	Digestive	GI.DUO.MUC	Duodenum Mucosa			H3K4me1_Enh				
E079	Digestive	GI.ESO	Esophagus							
E094	Digestive	GI.STMC.GAST	Gastric							
E099	Other	PLCNT.AMN	Placenta Amnion			H3K4me1_Enh				
E086	Other	KID.FET	Fetal Kidney							
E088	Other	LNG.FET	Fetal Lung							
E097	Other	OVRY	Ovary							
E087	Other	PANC.ISLT	Pancreatic Islets			H3K4me1_Enh		H3K27ac_Enh		
E080	Other	ADRL.GLND.FET	Fetal Adrenal Gland							
E091	Other	PLCNT.FET	Placenta							
E066	Other	LIV.ADLT	Liver							
E098	Other	PANC	Pancreas	6_EnhG		H3K4me1_Enh				
E096	Other	LNG	Lung							
E113	Other	SPLN	Spleen				H3K4me3_Pro			
E114	ENCODE2012	LNG.A549.ETOH002.CNCR	A549 EtOH 0.02pct Lung Carcinoma Cell Line							
E115	ENCODE2012	BLD.DND41.CNCR	Dnd41 TCell Leukemia Cell Line			H3K4me1_Enh				
E116	ENCODE2012	BLD.GM12878	GM12878 Lymphoblastoid Cells							
E117	ENCODE2012	CRVX.HELAS3.CNCR	HeLa-S3 Cervical Carcinoma Cell Line			H3K4me1_Enh				
E118	ENCODE2012	LIV.HEPG2.CNCR	HepG2 Hepatocellular Carcinoma Cell Line							
E119	ENCODE2012	BRST.HMEC	HMEC Mammary Epithelial Primary Cells							
E120	ENCODE2012	MUS.HSMM	HSMM Skeletal Muscle Myoblasts Cells							
E121	ENCODE2012	MUS.HSMMT	HSMM cell derived Skeletal Muscle Myotubes Cells							
E122	ENCODE2012	VAS.HUVEC	HUVEC Umbilical Vein Endothelial Primary Cells							
E123	ENCODE2012	BLD.K562.CNCR	K562 Leukemia Cells							
E124	ENCODE2012	BLD.CD14.MONO	Monocytes-CD14+ RO01746 Primary Cells					H3K27ac_Enh		
E125	ENCODE2012	BRN.NHA	NH-A Astrocytes Primary Cells							
E126	ENCODE2012	SKIN.NHDFAD	NHDF-Ad Adult Dermal Fibroblast Primary Cells							
E127	ENCODE2012	SKIN.NHEK	NHEK-Epidermal Keratinocyte Primary Cells						H3K9ac_Pro	
E128	ENCODE2012	LNG.NHLF	NHLF Lung Fibroblast Primary Cells							
E129	ENCODE2012	BONE.OSTEO	Osteoblast Primary Cells							

GRASP QTL hits

Trait	p-value	PMID
Differential exon level expression of MIS12 [probe 3707765] in brain cortex	8.03E-06	19222302
Differential exon level expression of MIS12 [probe 3707765] in peripheral blood mononuclear cells	1.43E-07	19222302
Gene expression of MIS12 [probe 3707759] in peripheral blood mononuclear cells	5.11E-05	19222302
Gene expression of MIS12 in blood	9.5E-05	21829388
Gene expression of NUP88 in blood	4.7E-07	21829388
Gene expression of MIS12 in normal prepouch ileum	1.10647E-05	23474282

Hits from selected eQTL studies

Study ID	Paper Title	PMID	Tissue	Correlated gene	p-value
Westra2013	Systematic identification of trans eQTLs as putative drivers of known disease associations	24013639	Whole_Blood	-	2.1513481094611317E-23
Westra2013	Systematic identification of trans eQTLs as putative drivers of known disease associations	24013639	Whole_Blood	MIS12	5.478876000038845E-15
Westra2013	Systematic identification of trans eQTLs as putative drivers of known disease associations	24013639	Whole_Blood	NUP88	7.488701797939842E-5

Regulatory motifs altered

Position Weight Matrix ID (Library from Kheradpour and Kellis, 2013)	Strand	Ref	Alt	Match on:<br>Ref: GAAATCCGCAGCAAGAAGCCTTGTGTGCTCAACTACTTTTCCAGCCTGGCAGGTTGAGC<br>Alt: GAAATCCGCAGCAAGAAGCCTTGTGTGCTAAACTACTTTTCCAGCCTGGCAGGTTGAGC
CEBPB_known4	+	7.8	10.1	DVWKTGSWDWNNN
SIX5_disc3	+	-10.3	1.6	AAACTACATTTCCCA
TCF12_disc2	-	12.6	11	TRTKKRCWBW

Detail view for rs11213916

Link to dbSNP entry

Link to Ensembl Variation entry

Sequence facts

chr	pos (hg19)	chr	pos (hg38)	Reference	Alternate	1000 Genomes Phase 1 Frequencies				Sequence constraint		dbSNP functional annotation
						AFR	AMR	ASN	EUR	by GERP	by SiPhy	
chr11	111338979	chr11	111468254	C	T	0.26	0.43	0.26	0.34	No	No	intronic

Closest annotated gene					
Source	Distance	Direction	ID/Link	Common name	Description
GENCODE	NA	Within gene	ENSG00000137707.9	BTG4	B-cell translocation gene 4 [Source:HGNC Symbol;Acc:13862]
RefSeq	NA	Within gene	NM_017589	BTG4	B-cell translocation gene 4 [Source:HGNC Symbol;Acc:13862]

Regulatory chromatin states from DNAse and histone ChIP-Seq (Roadmap Epigenomics Consortium, 2015)

(Black = missing data)

Epigenome ID (EID)	Group	Mnemonic	Description	Chromatin states (Core 15-state model)	Chromatin states (25-state model using 12 imputed marks)	H3K4me1	H3K4me3	H3K27ac	H3K9ac	DNas[illegible]
E017	IMR90	LNG.IMR90	IMR90 fetal lung fibroblasts Cell Line							
E002	ESC	ESC.WA7	ES-WA7 Cells							
E008	ESC	ESC.H9	H9 Cells							
E001	ESC	ESC.I3	ES-I3 Cells							
E015	ESC	ESC.HUES6	HUES6 Cells							
E014	ESC	ESC.HUES48	HUES48 Cells							
E016	ESC	ESC.HUES64	HUES64 Cells			H3K4me1_Enh				
E003	ESC	ESC.H1	H1 Cells							
E024	ESC	ESC.4STAR	ES-UCSF4 Cells							
E020	iPSC	IPSC.20B	iPS-20b Cells							
E019	iPSC	IPSC.18	iPS-18 Cells							
E018	iPSC	IPSC.15b	iPS-15b Cells							
E021	iPSC	IPSC.DF.6.9	iPS DF 6.9 Cells							
E022	iPSC	IPSC.DF.19.11	iPS DF 19.11 Cells							
E007	ES-deriv	ESDR.H1.NEUR.PROG	H1 Derived Neuronal Progenitor Cultured Cells							
E009	ES-deriv	ESDR.H9.NEUR.PROG	H9 Derived Neuronal Progenitor Cultured Cells							
E010	ES-deriv	ESDR.H9.NEUR	H9 Derived Neuron Cultured Cells							
E013	ES-deriv	ESDR.CD56.MESO	hESC Derived CD56+ Mesoderm Cultured Cells							
E012	ES-deriv	ESDR.CD56.ECTO	hESC Derived CD56+ Ectoderm Cultured Cells							
E011	ES-deriv	ESDR.CD184.ENDO	hESC Derived CD184+ Endoderm Cultured Cells							
E004	ES-deriv	ESDR.H1.BMP4.MESO	H1 BMP4 Derived Mesendoderm Cultured Cells							
E005	ES-deriv	ESDR.H1.BMP4.TROP	H1 BMP4 Derived Trophoblast Cultured Cells							
E006	ES-deriv	ESDR.H1.MSC	H1 Derived Mesenchymal Stem Cells							
E062	Blood & T-cell	BLD.PER.MONUC.PC	Primary mononuclear cells from�peripheral�blood							
E034	Blood & T-cell	BLD.CD3.PPC	Primary T cells from�peripheral�blood							
E045	Blood & T-cell	BLD.CD4.CD25I.CD127.TMEMPC	Primary T cells effector/memory enriched from peripheral blood							
E033	Blood & T-cell	BLD.CD3.CPC	Primary T cells from cord blood							
E044	Blood & T-cell	BLD.CD4.CD25.CD127M.TREGPC	Primary T regulatory cells from�peripheral�blood							
E043	Blood & T-cell	BLD.CD4.CD25M.TPC	Primary T helper cells from�peripheral�blood							
E039	Blood & T-cell	BLD.CD4.CD25M.CD45RA.NPC	Primary T helper naive cells from�peripheral�blood							
E041	Blood & T-cell	BLD.CD4.CD25M.IL17M.PL.TPC	Primary T helper cells PMA-I stimulated							
E042	Blood & T-cell	BLD.CD4.CD25M.IL17P.PL.TPC	Primary T helper 17 cells PMA-I stimulated							
E040	Blood & T-cell	BLD.CD4.CD25M.CD45RO.MPC	Primary T helper memory cells from peripheral blood 1							
E037	Blood & T-cell	BLD.CD4.MPC	Primary T helper memory cells from peripheral blood 2							

E048	Blood & T-cell	BLD.CD8.MPC	Primary T CD8+ memory cells from peripheral blood							
E038	Blood & T-cell	BLD.CD4.NPC	Primary T helper naive cells from peripheral blood							
E047	Blood & T-cell	BLD.CD8.NPC	Primary T CD8+ naive cells from peripheral blood							
E029	HSC & B-cell	BLD.CD14.PC	Primary monocytes from�peripheral�blood							
E031	HSC & B-cell	BLD.CD19.CPC	Primary B cells from cord blood							
E035	HSC & B-cell	BLD.CD34.PC	Primary hematopoietic stem cells							
E051	HSC & B-cell	BLD.MOB.CD34.PC.M	Primary hematopoietic stem cells G-CSF-mobilized Male							
E050	HSC & B-cell	BLD.MOB.CD34.PC.F	Primary hematopoietic stem cells G-CSF-mobilized Female							
E036	HSC & B-cell	BLD.CD34.CC	Primary hematopoietic stem cells short term culture							
E032	HSC & B-cell	BLD.CD19.PPC	Primary B cells from peripheral blood							
E046	HSC & B-cell	BLD.CD56.PC	Primary Natural Killer cells from�peripheral�blood							
E030	HSC & B-cell	BLD.CD15.PC	Primary neutrophils from�peripheral�blood							
E026	Mesench	STRM.MRW.MSC	Bone Marrow Derived Cultured Mesenchymal Stem Cells							
E049	Mesench	STRM.CHON.MRW.DR.MSC	Mesenchymal Stem Cell Derived Chondrocyte Cultured Cells							
E025	Mesench	FAT.ADIP.DR.MSC	Adipose Derived Mesenchymal Stem Cell Cultured Cells							
E023	Mesench	FAT.MSC.DR.ADIP	Mesenchymal Stem Cell Derived Adipocyte Cultured Cells							
E052	Myosat	MUS.SAT	Muscle Satellite Cultured Cells							
E055	Epithelial	SKIN.PEN.FRSK.FIB.01	Foreskin Fibroblast Primary Cells skin01							
E056	Epithelial	SKIN.PEN.FRSK.FIB.02	Foreskin Fibroblast Primary Cells skin02							
E059	Epithelial	SKIN.PEN.FRSK.MEL.01	Foreskin Melanocyte Primary Cells skin01							
E061	Epithelial	SKIN.PEN.FRSK.MEL.03	Foreskin Melanocyte Primary Cells skin03							
E057	Epithelial	SKIN.PEN.FRSK.KER.02	Foreskin Keratinocyte Primary Cells skin02							
E058	Epithelial	SKIN.PEN.FRSK.KER.03	Foreskin Keratinocyte Primary Cells skin03							
E028	Epithelial	BRST.HMEC.35	Breast variant Human Mammary Epithelial Cells (vHMEC)							
E027	Epithelial	BRST.MYO	Breast Myoepithelial Primary Cells							
E054	Neurosph	BRN.GANGEM.DR.NRSPHR	Ganglion Eminence derived primary cultured neurospheres							
E053	Neurosph	BRN.CRTX.DR.NRSPHR	Cortex derived primary cultured neurospheres							
E112	Thymus	THYM	Thymus							
E093	Thymus	THYM.FET	Fetal Thymus							
E071	Brain	BRN.HIPP.MID	Brain Hippocampus Middle							
E074	Brain	BRN.SUB.NIG	Brain Substantia Nigra							
E068	Brain	BRN.ANT.CAUD	Brain Anterior Caudate			H3K4me1_Enh		H3K27ac_Enh	H3K9ac_Pro	
E069	Brain	BRN.CING.GYR	Brain Cingulate Gyrus					H3K27ac_Enh		
E072	Brain	BRN.INF.TMP	Brain Inferior Temporal Lobe							
E067	Brain	BRN.ANG.GYR	Brain Angular Gyrus							
E073	Brain	BRN.DL.PRFRNTL.CRTX	Brain_Dorsolateral_Prefrontal_Cortex							
E070	Brain	BRN.GRM.MTRX	Brain Germinal Matrix							
E082	Brain	BRN.FET.F	Fetal Brain Female							
E081	Brain	BRN.FET.M	Fetal Brain Male							
E063	Adipose	FAT.ADIP.NUC	Adipose Nuclei							
E100	Muscle	MUS.PSOAS	Psoas Muscle			H3K4me1_Enh				
E108	Muscle	MUS.SKLT.F	Skeletal Muscle Female							
E107	Muscle	MUS.SKLT.M	Skeletal Muscle Male							
E089	Muscle	MUS.TRNK.FET	Fetal Muscle Trunk							
E090	Muscle	MUS.LEG.FET	Fetal Muscle Leg							
E083	Heart	HRT.FET	Fetal Heart							
E104	Heart	HRT.ATR.R	Right Atrium			H3K4me1_Enh				
E095	Heart	HRT.VENT.L	Left Ventricle							
E105	Heart	HRT.VNT.R	Right Ventricle							
E065	Heart	VAS.AOR	Aorta							
E078	Sm. Muscle	GI.DUO.SM.MUS	Duodenum Smooth Muscle							
E076	Sm. Muscle	GI.CLN.SM.MUS	Colon Smooth Muscle							
E103	Sm. Muscle	GI.RECT.SM.MUS	Rectal Smooth Muscle							
E111	Sm. Muscle	GI.STMC.MUS	Stomach Smooth Muscle					H3K27ac_Enh		
E092	Digestive	GI.STMC.FET	Fetal Stomach							
E085	Digestive	GI.S.INT.FET	Fetal Intestine Small							
E084	Digestive	GI.L.INT.FET	Fetal Intestine Large							
E109	Digestive	GI.S.INT	Small Intestine				H3K4me3_Pro			
E106	Digestive	GI.CLN.SIG	Sigmoid Colon							

E075	Digestive	GI.CLN.MUC	Colonic Mucosa							
E101	Digestive	GI.RECT.MUC.29	Rectal Mucosa Donor 29							
E102	Digestive	GI.RECT.MUC.31	Rectal Mucosa Donor 31							
E110	Digestive	GI.STMC.MUC	Stomach Mucosa							
E077	Digestive	GI.DUO.MUC	Duodenum Mucosa							
E079	Digestive	GI.ESO	Esophagus							
E094	Digestive	GI.STMC.GAST	Gastric							
E099	Other	PLCNT.AMN	Placenta Amnion							
E086	Other	KID.FET	Fetal Kidney							
E088	Other	LNG.FET	Fetal Lung							
E097	Other	OVRY	Ovary							
E087	Other	PANC.ISLT	Pancreatic Islets							
E080	Other	ADRL.GLND.FET	Fetal Adrenal Gland							
E091	Other	PLCNT.FET	Placenta							
E066	Other	LIV.ADLT	Liver							
E098	Other	PANC	Pancreas							
E096	Other	LNG	Lung							
E113	Other	SPLN	Spleen							
E114	ENCODE2012	LNG.A549.ETOH002.CNCR	A549 EtOH 0.02pct Lung Carcinoma Cell Line							
E115	ENCODE2012	BLD.DND41.CNCR	Dnd41 TCell Leukemia Cell Line							
E116	ENCODE2012	BLD.GM12878	GM12878 Lymphoblastoid Cells							
E117	ENCODE2012	CRVX.HELAS3.CNCR	HeLa-S3 Cervical Carcinoma Cell Line							
E118	ENCODE2012	LIV.HEPG2.CNCR	HepG2 Hepatocellular Carcinoma Cell Line							
E119	ENCODE2012	BRST.HMEC	HMEC Mammary Epithelial Primary Cells							
E120	ENCODE2012	MUS.HSMM	HSMM Skeletal Muscle Myoblasts Cells							
E121	ENCODE2012	MUS.HSMMT	HSMM cell derived Skeletal Muscle Myotubes Cells							
E122	ENCODE2012	VAS.HUVEC	HUVEC Umbilical Vein Endothelial Primary Cells							
E123	ENCODE2012	BLD.K562.CNCR	K562 Leukemia Cells							
E124	ENCODE2012	BLD.CD14.MONO	Monocytes-CD14+ RO01746 Primary Cells							
E125	ENCODE2012	BRN.NHA	NH-A Astrocytes Primary Cells							
E126	ENCODE2012	SKIN.NHDFAD	NHDF-Ad Adult Dermal Fibroblast Primary Cells							
E127	ENCODE2012	SKIN.NHEK	NHEK-Epidermal Keratinocyte Primary Cells							
E128	ENCODE2012	LNG.NHLF	NHLF Lung Fibroblast Primary Cells							
E129	ENCODE2012	BONE.OSTEO	Osteoblast Primary Cells			H3K4me1_Enh				

Detail view for rs1037791

Link to dbSNP entry

Link to Ensembl Variation entry

Sequence facts

chr	pos (hg19)	chr	pos (hg38)	Reference	Alternate	1000 Genomes Phase 1 Frequencies				Sequence constraint		dbSNP functional annotation
						AFR	AMR	ASN	EUR	by GERP	by SiPhy	
chr7	16824662	chr7	16785037	G	A	0.63	0.77	0.72	0.74	No	No	none

Closest annotated gene					
Source	Distance	Direction	ID/Link	Common name	Description
GENCODE	3'	500	ENSG00000106537.7	TSPAN13	tetraspanin 13 [Source:HGNC Symbol;Acc:21643]
RefSeq	3'	500	NM_014399	TSPAN13	tetraspanin 13 [Source:HGNC Symbol;Acc:21643]

Regulatory chromatin states from DNAse and histone ChIP-Seq (Roadmap Epigenomics Consortium, 2015)

(Black = missing data)

Epigenome ID (EID)	Group	Mnemonic	Description	Chromatin states (Core 15-state model)	Chromatin states (25-state model using 12 imputed marks)	H3K4me1	H3K4me3	H3K27ac	H3K9ac	DNas
E017	IMR90	LNG.IMR90	IMR90 fetal lung fibroblasts Cell Line							
E002	ESC	ESC.WA7	ES-WA7 Cells							
E008	ESC	ESC.H9	H9 Cells							
E001	ESC	ESC.I3	ES-I3 Cells							
E015	ESC	ESC.HUES6	HUES6 Cells							
E014	ESC	ESC.HUES48	HUES48 Cells							
E016	ESC	ESC.HUES64	HUES64 Cells							
E003	ESC	ESC.H1	H1 Cells							
E024	ESC	ESC.4STAR	ES-UCSF4 Cells							
E020	iPSC	IPSC.20B	iPS-20b Cells							
E019	iPSC	IPSC.18	iPS-18 Cells							
E018	iPSC	IPSC.15b	iPS-15b Cells							
E021	iPSC	IPSC.DF.6.9	iPS DF 6.9 Cells				H3K4me3_Pro			
E022	iPSC	IPSC.DF.19.11	iPS DF 19.11 Cells							
E007	ES-deriv	ESDR.H1.NEUR.PROG	H1 Derived Neuronal Progenitor Cultured Cells							
E009	ES-deriv	ESDR.H9.NEUR.PROG	H9 Derived Neuronal Progenitor Cultured Cells							
E010	ES-deriv	ESDR.H9.NEUR	H9 Derived Neuron Cultured Cells			H3K4me1_Enh				
E013	ES-deriv	ESDR.CD56.MESO	hESC Derived CD56+ Mesoderm Cultured Cells			H3K4me1_Enh				
E012	ES-deriv	ESDR.CD56.ECTO	hESC Derived CD56+ Ectoderm Cultured Cells							
E011	ES-deriv	ESDR.CD184.ENDO	hESC Derived CD184+ Endoderm Cultured Cells			H3K4me1_Enh				
E004	ES-deriv	ESDR.H1.BMP4.MESO	H1 BMP4 Derived Mesendoderm Cultured Cells							
E005	ES-deriv	ESDR.H1.BMP4.TROP	H1 BMP4 Derived Trophoblast Cultured Cells							
E006	ES-deriv	ESDR.H1.MSC	H1 Derived Mesenchymal Stem Cells							
E062	Blood & T-cell	BLD.PER.MONUC.PC	Primary mononuclear cells from�peripheral�blood							
E034	Blood & T-cell	BLD.CD3.PPC	Primary T cells from�peripheral�blood							
E045	Blood & T-cell	BLD.CD4.CD25I.CD127.TMEMPC	Primary T cells effector/memory enriched from peripheral blood							
E033	Blood & T-cell	BLD.CD3.CPC	Primary T cells from cord blood							
E044	Blood & T-cell	BLD.CD4.CD25.CD127M.TREGPC	Primary T regulatory cells from�peripheral�blood							
E043	Blood & T-cell	BLD.CD4.CD25M.TPC	Primary T helper cells from�peripheral�blood							
E039	Blood & T-cell	BLD.CD4.CD25M.CD45RA.NPC	Primary T helper naive cells from�peripheral�blood							
E041	Blood & T-cell	BLD.CD4.CD25M.IL17M.PL.TPC	Primary T helper cells PMA-I stimulated							
E042	Blood & T-cell	BLD.CD4.CD25M.IL17P.PL.TPC	Primary T helper 17 cells PMA-I stimulated							
E040	Blood & T-cell	BLD.CD4.CD25M.CD45RO.MPC	Primary T helper memory cells from peripheral blood 1							
E037	Blood & T-cell	BLD.CD4.MPC	Primary T helper memory cells from peripheral blood 2							

E048	Blood & T-cell	BLD.CD8.MPC	Primary T CD8+ memory cells from peripheral blood							
E038	Blood & T-cell	BLD.CD4.NPC	Primary T helper naive cells from peripheral blood							
E047	Blood & T-cell	BLD.CD8.NPC	Primary T CD8+ naive cells from peripheral blood							
E029	HSC & B-cell	BLD.CD14.PC	Primary monocytes from�peripheral�blood							
E031	HSC & B-cell	BLD.CD19.CPC	Primary B cells from cord blood							
E035	HSC & B-cell	BLD.CD34.PC	Primary hematopoietic stem cells							
E051	HSC & B-cell	BLD.MOB.CD34.PC.M	Primary hematopoietic stem cells G-CSF-mobilized Male							
E050	HSC & B-cell	BLD.MOB.CD34.PC.F	Primary hematopoietic stem cells G-CSF-mobilized Female							
E036	HSC & B-cell	BLD.CD34.CC	Primary hematopoietic stem cells short term culture							
E032	HSC & B-cell	BLD.CD19.PPC	Primary B cells from peripheral blood							
E046	HSC & B-cell	BLD.CD56.PC	Primary Natural Killer cells from�peripheral�blood							
E030	HSC & B-cell	BLD.CD15.PC	Primary neutrophils from�peripheral�blood							
E026	Mesench	STRM.MRW.MSC	Bone Marrow Derived Cultured Mesenchymal Stem Cells							
E049	Mesench	STRM.CHON.MRW.DR.MSC	Mesenchymal Stem Cell Derived Chondrocyte Cultured Cells			H3K4me1_Enh		H3K27ac_Enh		
E025	Mesench	FAT.ADIP.DR.MSC	Adipose Derived Mesenchymal Stem Cell Cultured Cells							
E023	Mesench	FAT.MSC.DR.ADIP	Mesenchymal Stem Cell Derived Adipocyte Cultured Cells							
E052	Myosat	MUS.SAT	Muscle Satellite Cultured Cells							
E055	Epithelial	SKIN.PEN.FRSK.FIB.01	Foreskin Fibroblast Primary Cells skin01							
E056	Epithelial	SKIN.PEN.FRSK.FIB.02	Foreskin Fibroblast Primary Cells skin02							
E059	Epithelial	SKIN.PEN.FRSK.MEL.01	Foreskin Melanocyte Primary Cells skin01							
E061	Epithelial	SKIN.PEN.FRSK.MEL.03	Foreskin Melanocyte Primary Cells skin03					H3K27ac_Enh		
E057	Epithelial	SKIN.PEN.FRSK.KER.02	Foreskin Keratinocyte Primary Cells skin02							
E058	Epithelial	SKIN.PEN.FRSK.KER.03	Foreskin Keratinocyte Primary Cells skin03							
E028	Epithelial	BRST.HMEC.35	Breast variant Human Mammary Epithelial Cells (vHMEC)							
E027	Epithelial	BRST.MYO	Breast Myoepithelial Primary Cells							
E054	Neurosph	BRN.GANGEM.DR.NRSPHR	Ganglion Eminence derived primary cultured neurospheres							
E053	Neurosph	BRN.CRTX.DR.NRSPHR	Cortex derived primary cultured neurospheres							
E112	Thymus	THYM	Thymus							
E093	Thymus	THYM.FET	Fetal Thymus							
E071	Brain	BRN.HIPP.MID	Brain Hippocampus Middle							
E074	Brain	BRN.SUB.NIG	Brain Substantia Nigra							
E068	Brain	BRN.ANT.CAUD	Brain Anterior Caudate							
E069	Brain	BRN.CING.GYR	Brain Cingulate Gyrus							
E072	Brain	BRN.INF.TMP	Brain Inferior Temporal Lobe					H3K27ac_Enh		
E067	Brain	BRN.ANG.GYR	Brain Angular Gyrus			H3K4me1_Enh		H3K27ac_Enh		
E073	Brain	BRN.DL.PRFRNTL.CRTX	Brain_Dorsolateral_Prefrontal_Cortex							
E070	Brain	BRN.GRM.MTRX	Brain Germinal Matrix							
E082	Brain	BRN.FET.F	Fetal Brain Female							
E081	Brain	BRN.FET.M	Fetal Brain Male							
E063	Adipose	FAT.ADIP.NUC	Adipose Nuclei							
E100	Muscle	MUS.PSOAS	Psoas Muscle							
E108	Muscle	MUS.SKLT.F	Skeletal Muscle Female							
E107	Muscle	MUS.SKLT.M	Skeletal Muscle Male							
E089	Muscle	MUS.TRNK.FET	Fetal Muscle Trunk					H3K27ac_Enh		
E090	Muscle	MUS.LEG.FET	Fetal Muscle Leg			H3K4me1_Enh				
E083	Heart	HRT.FET	Fetal Heart							
E104	Heart	HRT.ATR.R	Right Atrium							
E095	Heart	HRT.VENT.L	Left Ventricle							
E105	Heart	HRT.VNT.R	Right Ventricle							
E065	Heart	VAS.AOR	Aorta							
E078	Sm. Muscle	GI.DUO.SM.MUS	Duodenum Smooth Muscle							
E076	Sm. Muscle	GI.CLN.SM.MUS	Colon Smooth Muscle							
E103	Sm. Muscle	GI.RECT.SM.MUS	Rectal Smooth Muscle							
E111	Sm. Muscle	GI.STMC.MUS	Stomach Smooth Muscle							
E092	Digestive	GI.STMC.FET	Fetal Stomach							
E085	Digestive	GI.S.INT.FET	Fetal Intestine Small			H3K4me1_Enh				
E084	Digestive	GI.L.INT.FET	Fetal Intestine Large							
E109	Digestive	GI.S.INT	Small Intestine							
E106	Digestive	GI.CLN.SIG	Sigmoid Colon							
E075	Digestive	GI.CLN.MUC	Colonic Mucosa							

E101	Digestive	GI.RECT.MUC.29	Rectal Mucosa Donor 29							
E102	Digestive	GI.RECT.MUC.31	Rectal Mucosa Donor 31			H3K4me1_Enh				
E110	Digestive	GI.STMC.MUC	Stomach Mucosa			H3K4me1_Enh			H3K9ac_Pro	
E077	Digestive	GI.DUO.MUC	Duodenum Mucosa							
E079	Digestive	GI.ESO	Esophagus							
E094	Digestive	GI.STMC.GAST	Gastric					H3K27ac_Enh		
E099	Other	PLCNT.AMN	Placenta Amnion			H3K4me1_Enh		H3K27ac_Enh		
E086	Other	KID.FET	Fetal Kidney							
E088	Other	LNG.FET	Fetal Lung						H3K9ac_Pro	
E097	Other	OVRY	Ovary							
E087	Other	PANC.ISLT	Pancreatic Islets			H3K4me1_Enh				
E080	Other	ADRL.GLND.FET	Fetal Adrenal Gland					H3K27ac_Enh		
E091	Other	PLCNT.FET	Placenta							
E066	Other	LIV.ADLT	Liver							
E098	Other	PANC	Pancreas					H3K27ac_Enh		
E096	Other	LNG	Lung							
E113	Other	SPLN	Spleen							
E114	ENCODE2012	LNG.A549.ETOH002.CNCR	A549 EtOH 0.02pct Lung Carcinoma Cell Line							
E115	ENCODE2012	BLD.DND41.CNCR	Dnd41 TCell Leukemia Cell Line							
E116	ENCODE2012	BLD.GM12878	GM12878 Lymphoblastoid Cells							
E117	ENCODE2012	CRVX.HELAS3.CNCR	HeLa-S3 Cervical Carcinoma Cell Line							
E118	ENCODE2012	LIV.HEPG2.CNCR	HepG2 Hepatocellular Carcinoma Cell Line							
E119	ENCODE2012	BRST.HMEC	HMEC Mammary Epithelial Primary Cells							
E120	ENCODE2012	MUS.HSMM	HSMM Skeletal Muscle Myoblasts Cells							
E121	ENCODE2012	MUS.HSMMT	HSMM cell derived Skeletal Muscle Myotubes Cells					H3K27ac_Enh		
E122	ENCODE2012	VAS.HUVEC	HUVEC Umbilical Vein Endothelial Primary Cells							
E123	ENCODE2012	BLD.K562.CNCR	K562 Leukemia Cells							
E124	ENCODE2012	BLD.CD14.MONO	Monocytes-CD14+ RO01746 Primary Cells							
E125	ENCODE2012	BRN.NHA	NH-A Astrocytes Primary Cells							
E126	ENCODE2012	SKIN.NHDFAD	NHDF-Ad Adult Dermal Fibroblast Primary Cells							
E127	ENCODE2012	SKIN.NHEK	NHEK-Epidermal Keratinocyte Primary Cells							
E128	ENCODE2012	LNG.NHLF	NHLF Lung Fibroblast Primary Cells							
E129	ENCODE2012	BONE.OSTEO	Osteoblast Primary Cells							

Regulatory motifs altered

**Position Weight Matrix ID (Library from Kheradpour and Kellis, 2013)**	**Strand**	**Ref**	**Alt**	**Match on:** Ref: AACAGTGTCCCCACTTATACCATAAGATGGGATTATAAGTGACATATGAAGGGAGATTT Alt: AACAGTGTCCCCACTTATACCATAAGATGAGATTATAAGTGACATATGAAGGGAGATTT
AIRE_1	-	9.4	11.2	WWDHVWCCDWWHNWWCCWDDWHHWDW
CTCF_disc1	-	11.8	9	DCCASTAGRKGGYRSYVKWDNH
Crx_1	+	11.6	11.3	VKVRGATTARDVR
Evi-1_3	+	-7.5	4.4	AGATAAGATAA
Obox6	+	12.7	8.1	MNAWDSGGATTAWHB
