# Supplementary figures and images for: DRD2 co-expression network and a related polygenic index predict imaging, behavioral and clinical phenotypes linked to schizophrenia
Source: Transl Psychiatry. 2017 Jan 17;7(1):e1006–. doi: 10.1038/tp.2016.253 (PMC5545721; doi:10.1038/tp.2016.253)

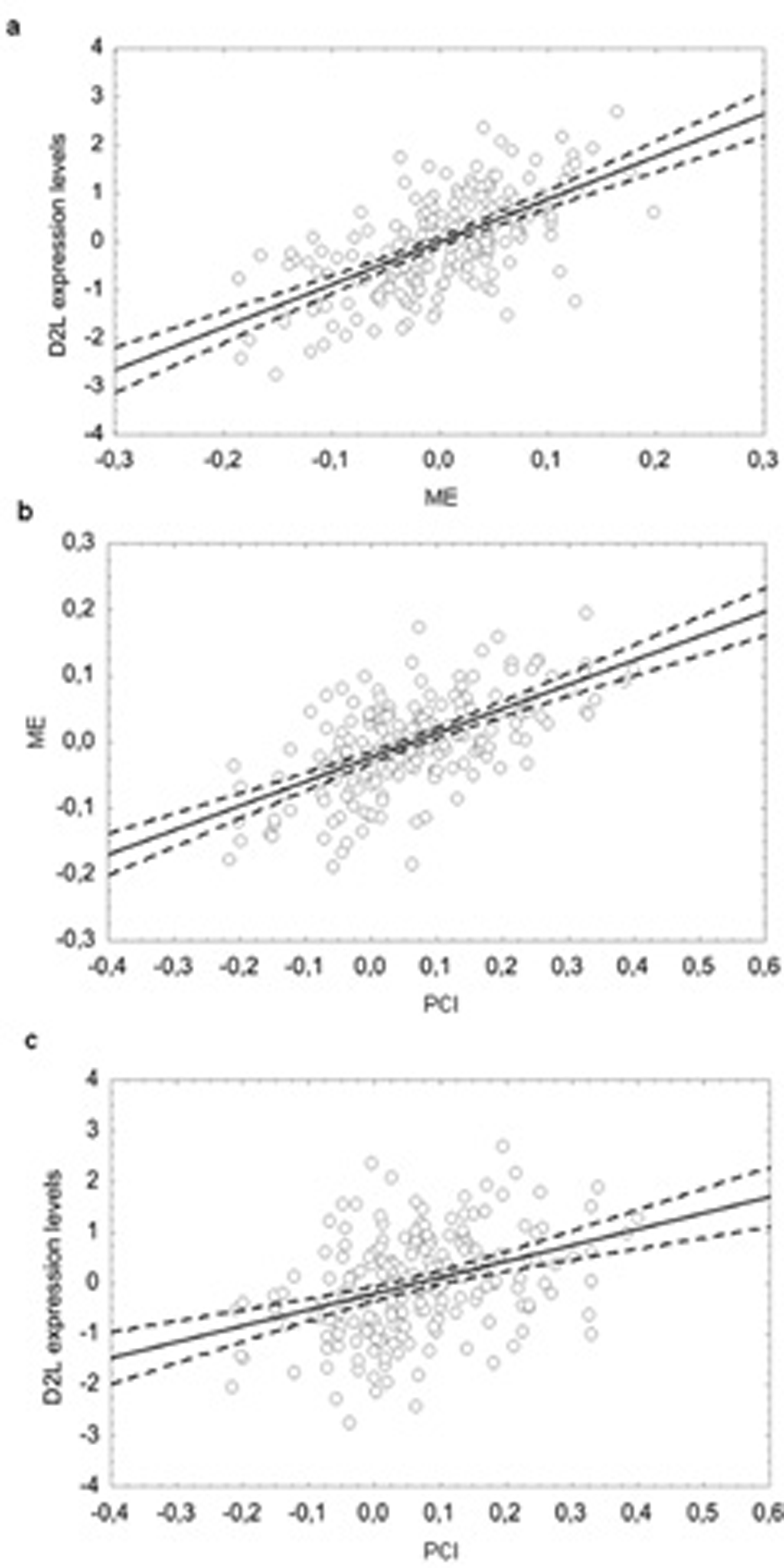

Supplement: Supplementary Figure 1 [file tp2016253x3.tif]

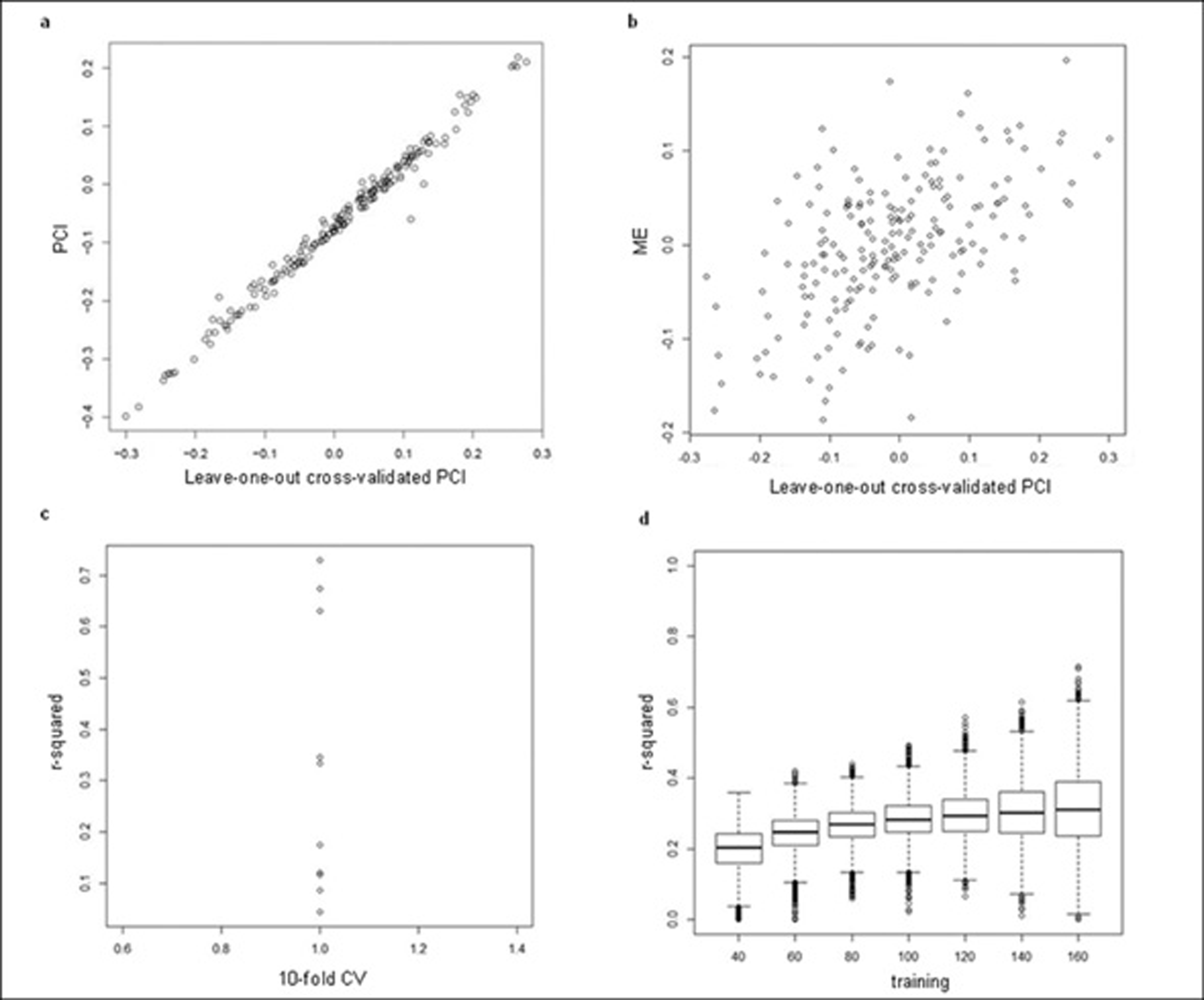

Supplement: Supplementary Figure 2 [file tp2016253x4.tif]

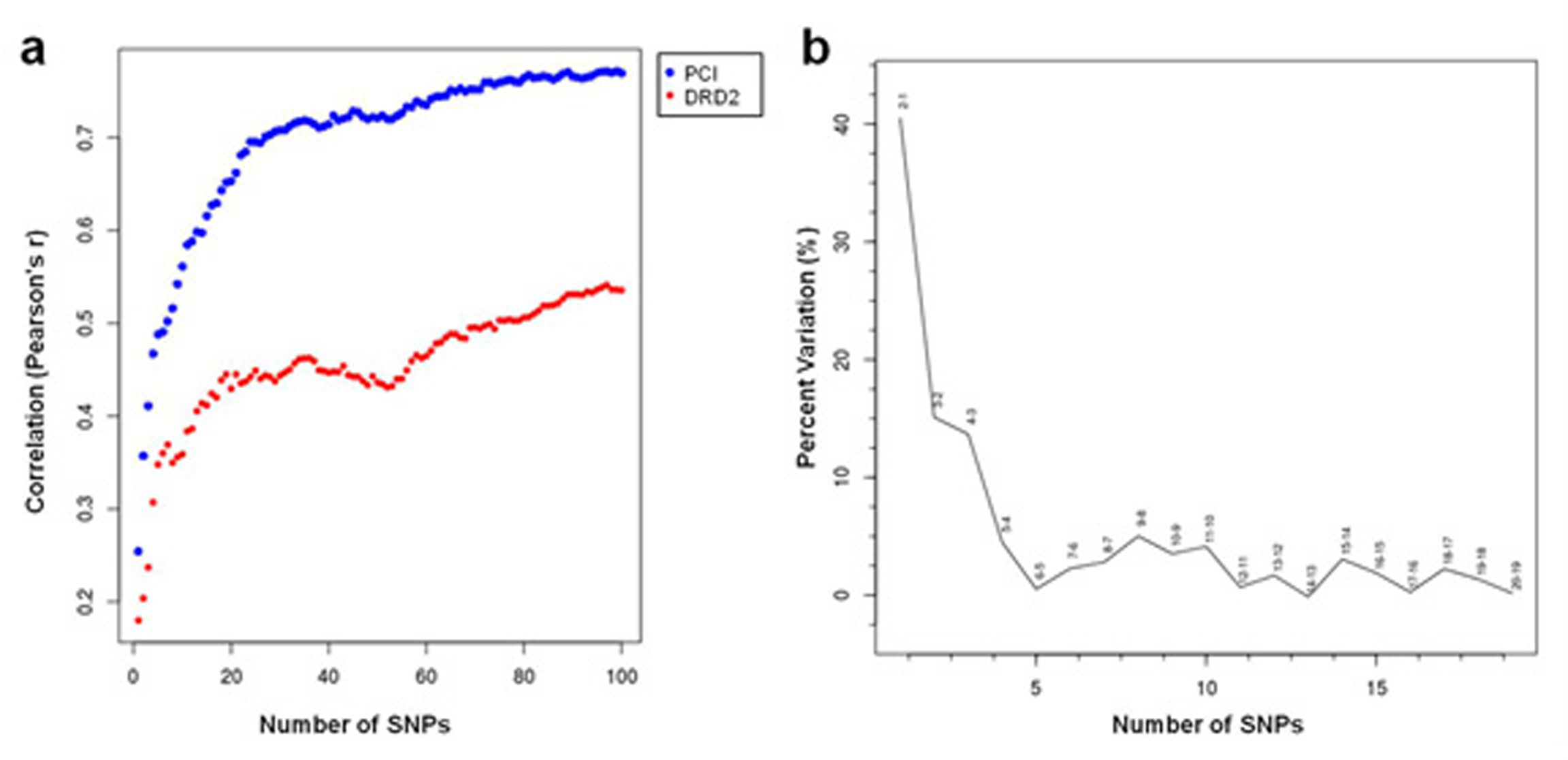

Supplement: Supplementary Figure 3 [file tp2016253x5.tif]

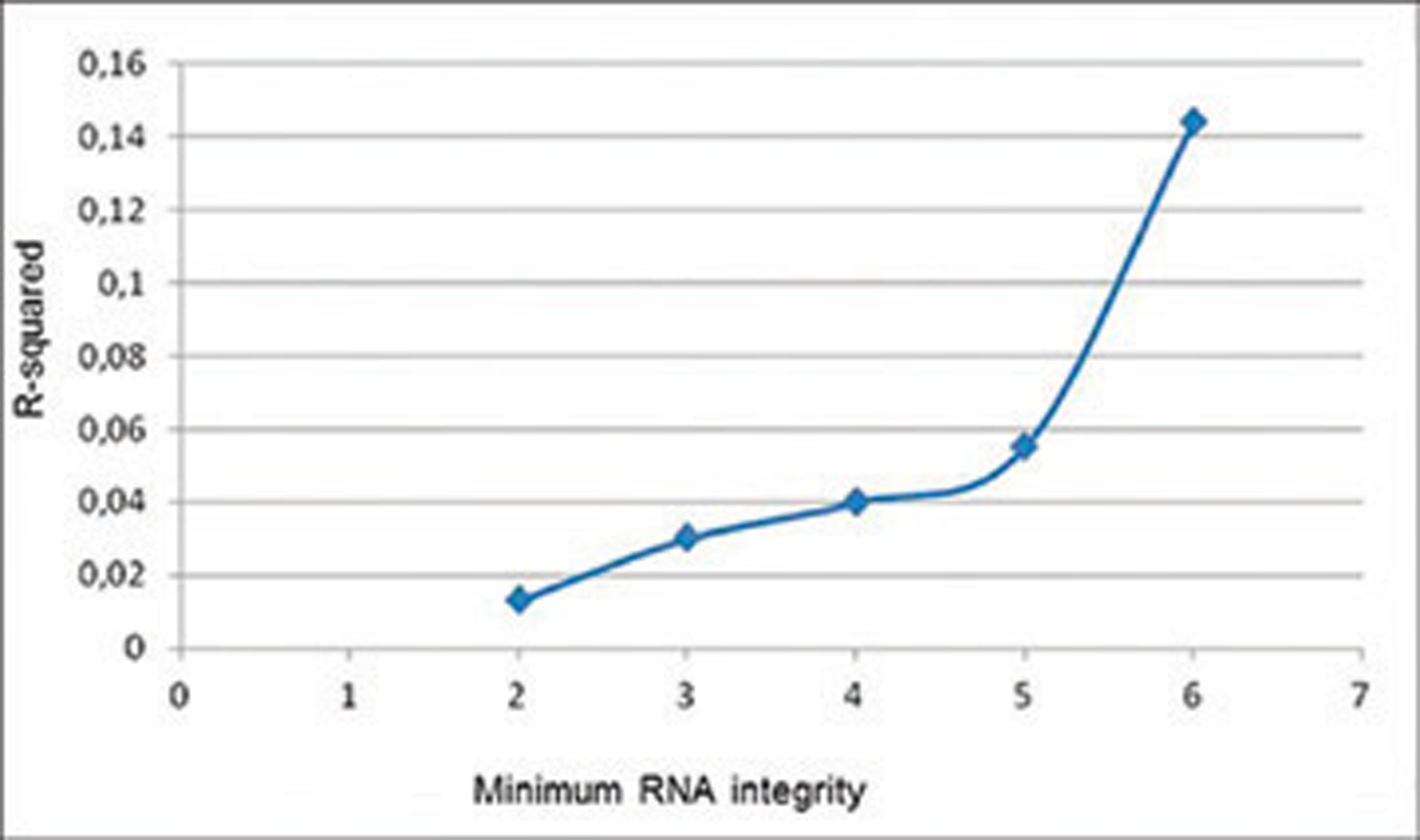

Supplement: Supplementary Figure 4 [file tp2016253x6.tif]

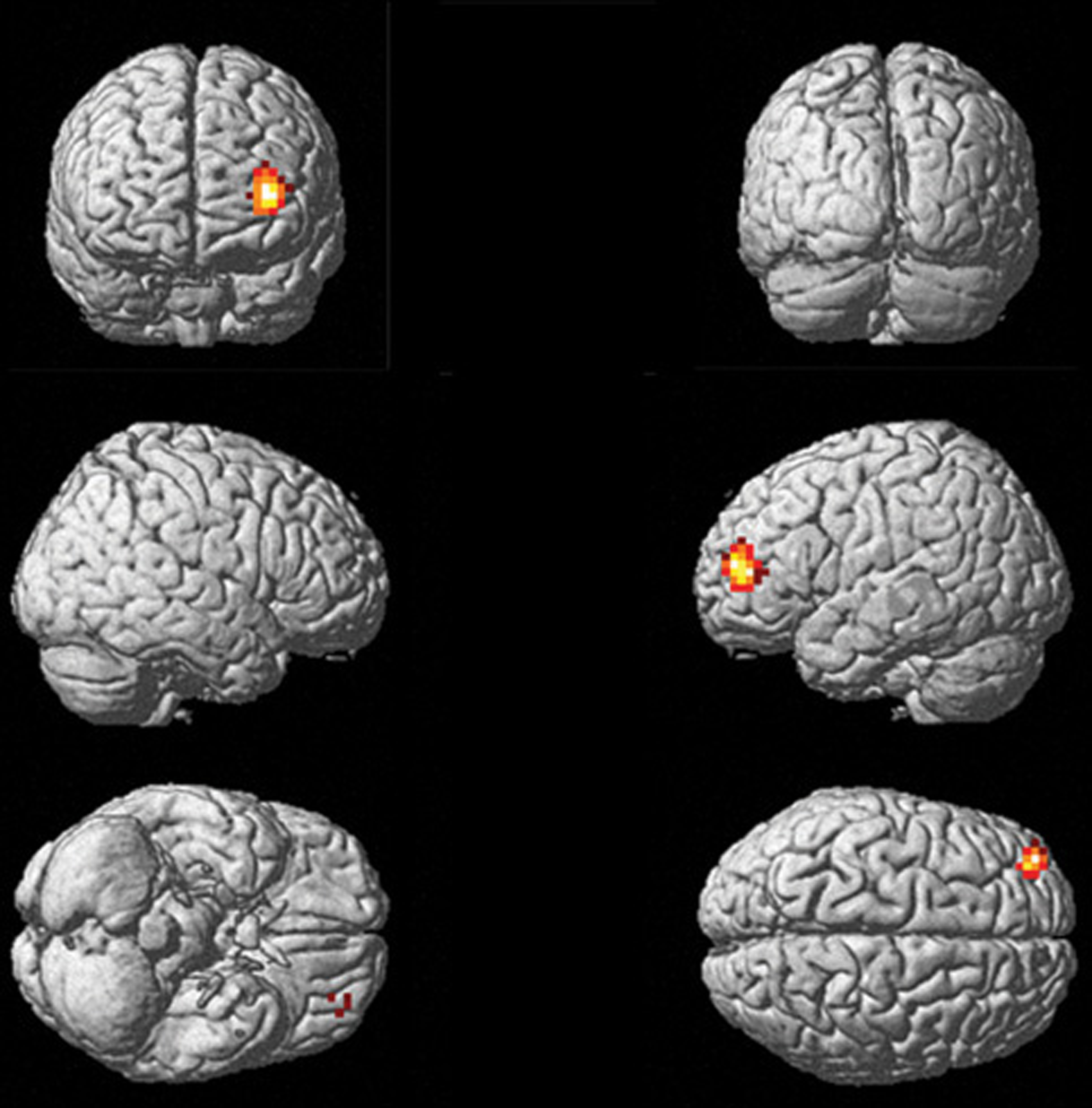

Supplement: Supplementary Figure 5 [file tp2016253x7.tif]

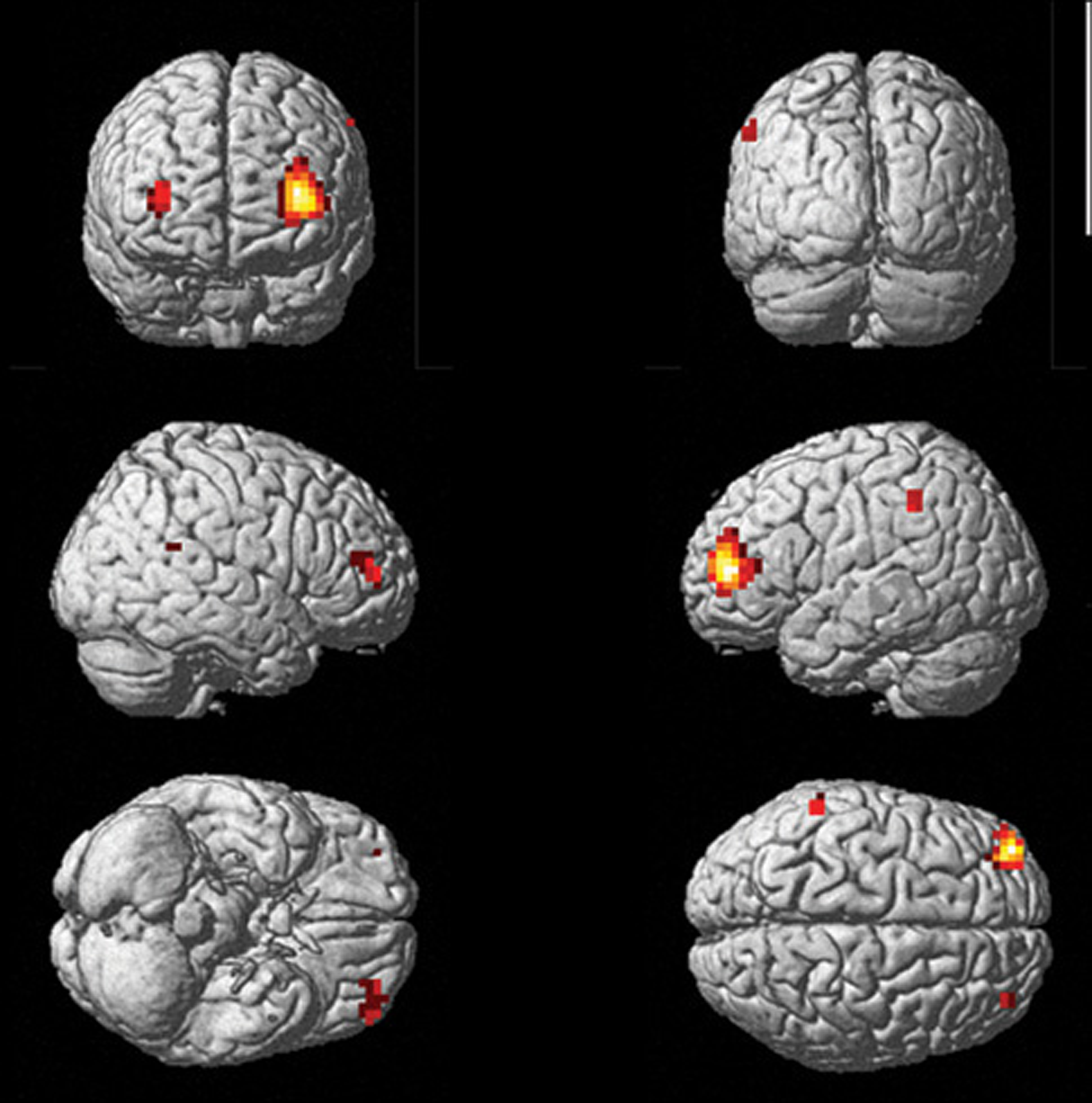

Supplement: Supplementary Figure 6 [file tp2016253x8.tif]

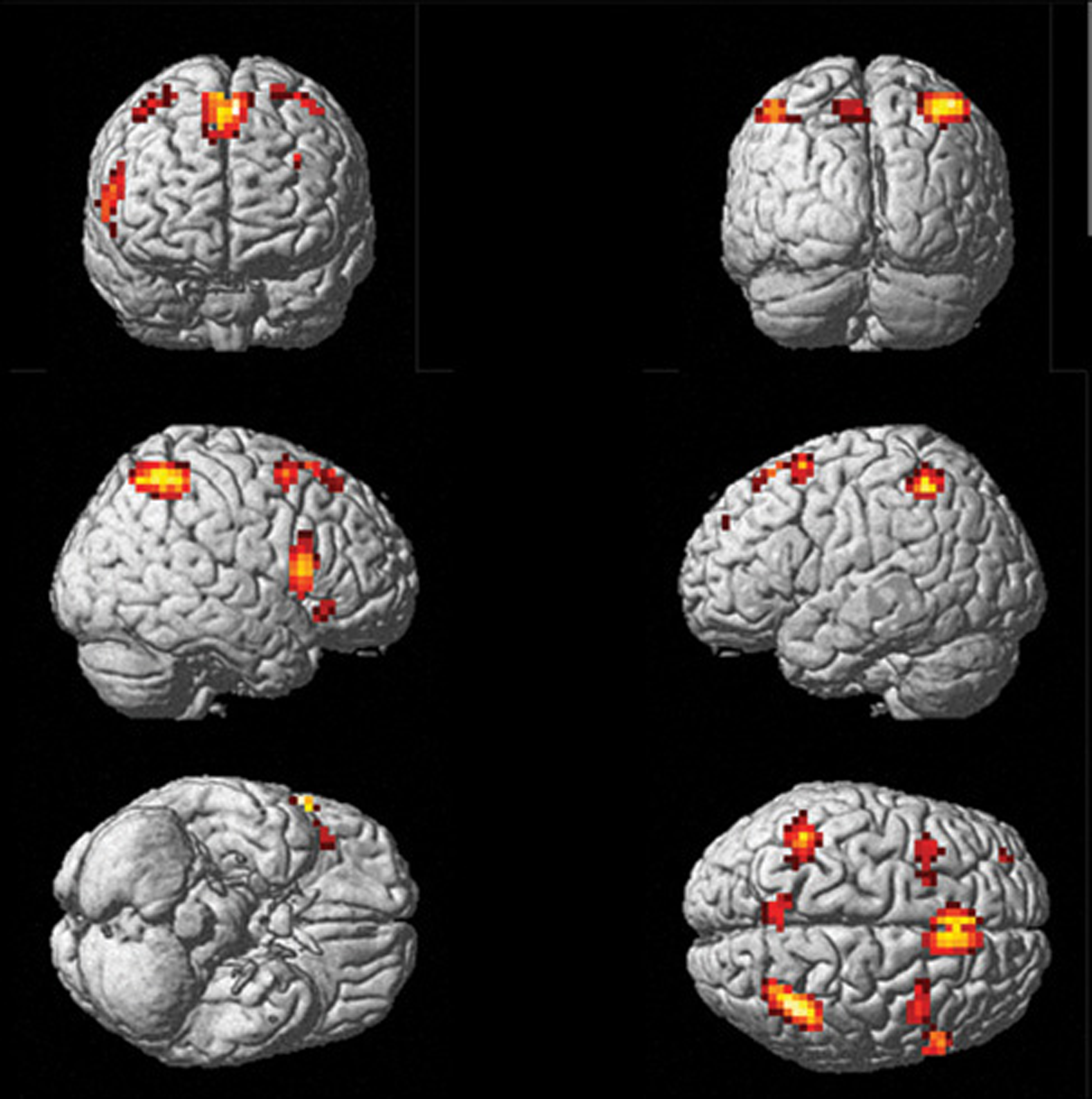

Supplement: Supplementary Figure 7 [file tp2016253x9.tif]

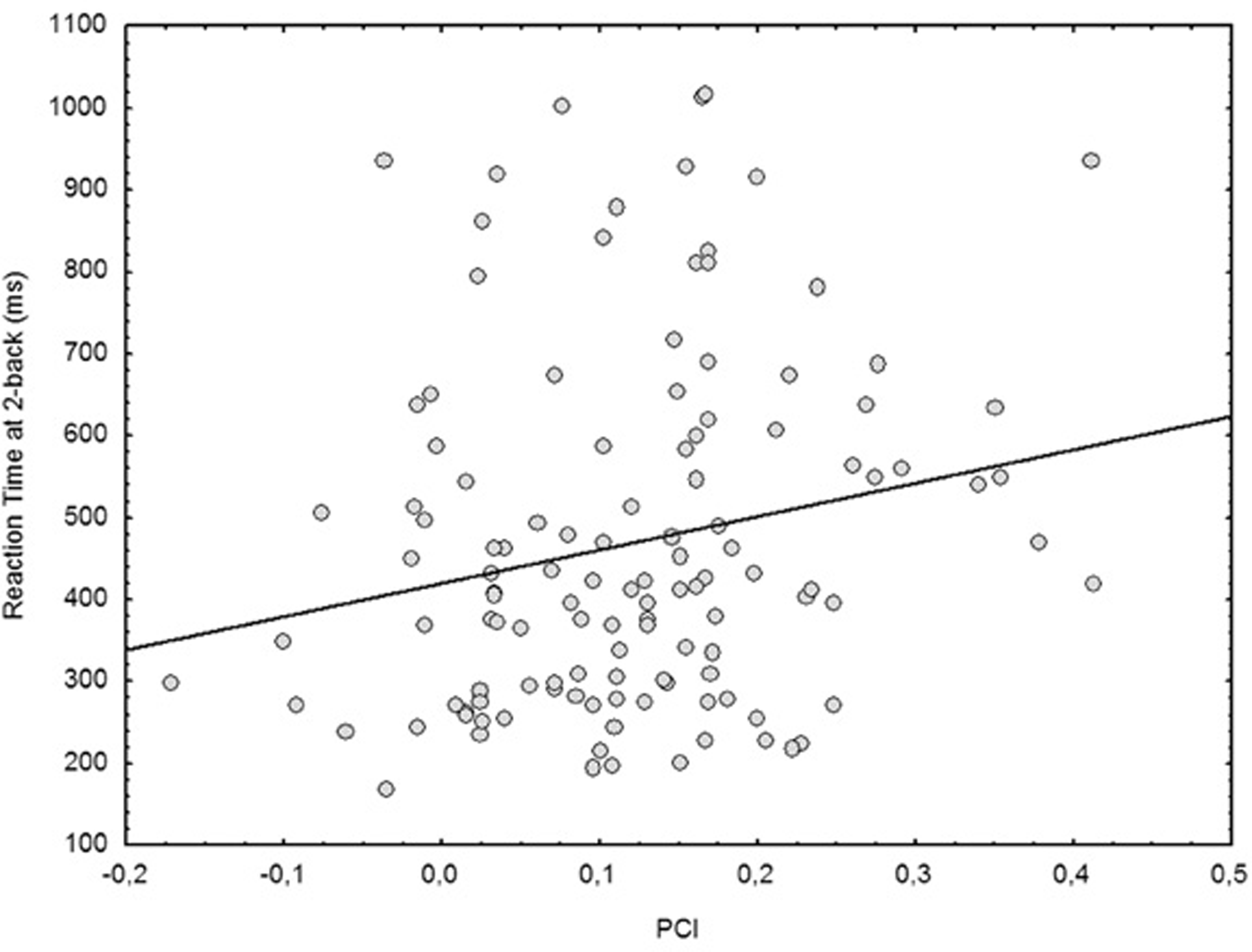

Supplement: Supplementary Figure 8 [file tp2016253x10.tif]

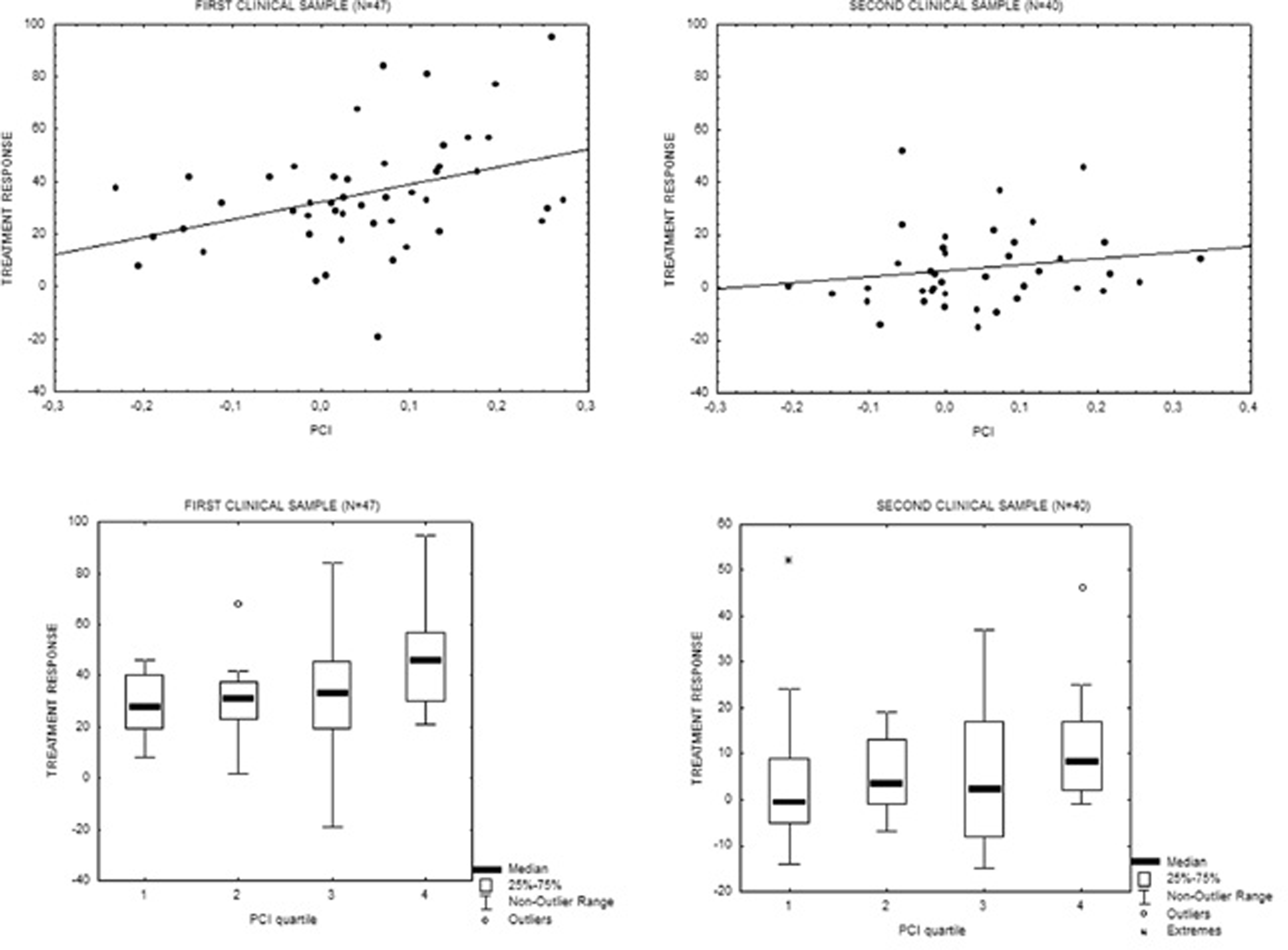

Supplement: Supplementary Figure 9 [file tp2016253x11.tif]

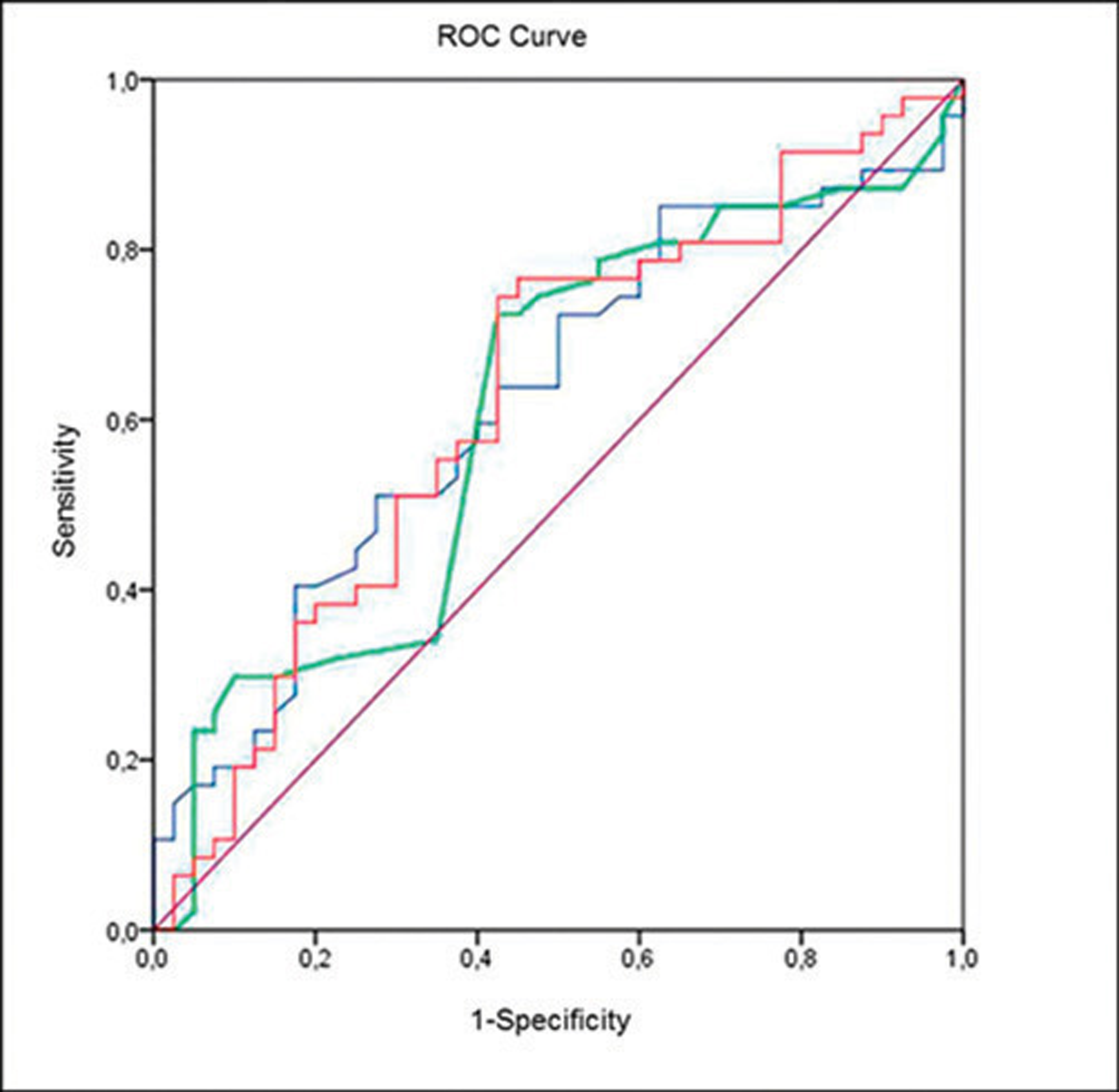

Supplement: Supplementary Figure 10 [file tp2016253x12.tif]
